# Chemoenzymatic Synthesis and Biological Evaluation for Bioactive Molecules Derived from Bacterial Benzoyl Coenzyme A Ligase and Plant Type III Polyketide Synthase

**DOI:** 10.3390/biom10050738

**Published:** 2020-05-09

**Authors:** Kamal Adhikari, I-Wen Lo, Chun-Liang Chen, Yung-Lin Wang, Kuan-Hung Lin, Saeid Malek Zadeh, Rajesh Rattinam, Yi-Shan Li, Chang-Jer Wu, Tsung-Lin Li

**Affiliations:** 1 Genomics Research Center, Academia Sinica, Taipei 11529, Taiwan; arsonkamal@yahoo.com (K.A.); iwenlo99@gmail.com (I-W.L.); muzhikmuzhik@gmail.com (C.-L.C.); hcvns3@hotmail.com (Y.-L.W.); cardboy19821022@hotmail.com (K.-H.L.); saeid.malekzadeh@gmail.com (S.M.Z.); rajeshbuibt@gmail.com (R.R.); leuisa@gate.sinica.edu.tw (Y.-S.L.); 2 Molecular and Biological Agricultural Sciences Program, Taiwan International Graduate Program, Academia Sinica and National Chung Hsing University, Taipei 11529, Taiwan; 3 Graduate Institute of Biotechnology, National Chung Hsing University, Taichung 40227, Taiwan; 4 Chemical Biology and Molecular Biophysics Program, Taiwan International Graduate Program, Taipei 11529, Taiwan; 5 Institute of Bioinformatics and Structural Biology, National Tsing Hua University, Hsinchu 30013, Taiwan; 6 Department of Food Science, National Taiwan Ocean University, Keelung 20224, Taiwan; cjwu@mail.ntou.edu.tw; 7 Biotechnology Center, National Chung Hsing University, Taichung 40227, Taiwan

**Keywords:** polyketide synthase, benzoate coenzyme A ligase, protein engineering, apoptosis, cytotoxicity

## Abstract

Plant type III polyketide synthases produce diverse bioactive molecules with a great medicinal significance to human diseases. Here, we demonstrated versatility of a stilbene synthase (STS) from *Pinus Sylvestris*, which can accept various non-physiological substrates to form unnatural polyketide products. Three enzymes (4-coumarate CoA ligase, malonyl-CoA synthetase and engineered benzoate CoA ligase) along with synthetic chemistry was practiced to synthesize starter and extender substrates for STS. Of these, the crystal structures of benzoate CoA ligase (BadA) from *Rhodopseudomonas palustris* in an *apo* form or in complex with a 2-chloro-1,3-thiazole-5-carboxyl-AMP or 2-methylthiazole-5-carboxyl-AMP intermediate were determined at resolutions of 1.57 Å, 1.7 Å, and 2.13 Å, respectively, which reinforces its capacity in production of unusual CoA starters. STS exhibits broad substrate promiscuity effectively affording structurally diverse polyketide products. Seven novel products showed desired cytotoxicity against a panel of cancer cell lines (A549, HCT116, Cal27). With the treatment of two selected compounds, the cancer cells underwent cell apoptosis in a dose-dependent manner. The precursor-directed biosynthesis alongside structure-guided enzyme engineering greatly expands the pharmaceutical repertoire of lead compounds with promising/enhanced biological activities.

## 1. Introduction

More than 7000 polyketides, a group of secondary metabolites that are produced from plants, bacteria, and fungi, have been identified [1]. Among these compounds over 20 chemical entities have been commercially developed as medicines, making this class of natural products an attractive source to discover drugs such as antibiotics, anti-cancer, anti-cholesterol agents, and many others [1,2]. In nature these molecules are biosynthesized through decarboxylative condensation of carboxylic acids by polyketide synthases (PKSs). On the basis of chemical structures, gene/polypeptide sequence similarity and formation mechanisms PKSs are divided into three groups: PKS I, PKS II, and PKS III. Type III PKSs are structurally and mechanistically different from the modular PKS I and dissociated PKS II. Unlike others type III PKSs usually utilize free CoA thioesters as substrates (type I and II use substrates linked to the 4′-phosphopantetheine residue of acyl carrier protein (ACP)) without the involvement of ACP and are able to complete a series of decarboxylative condensation, cyclization and aromatization reactions in a single active site. This type of PKSs III produces a wide array of polyketides products. The diversification of product formation depends on the choice of starter (aliphatic or aromatic acyl-CoAs) and extender units (malonyl-CoA and/or its derivatives), and different in intramolecular cyclization (Claisen condensation, aldol condensation, or heterocyclic lactonization) of the linear polyketide intermediate. The polyketide products from some plant type III PKSs show many desired biological properties such as anti-oxidant, antibacterial, anticancer, antiviral, antifungal, immune-suppressing, anti-cholesterol, and anti-inflammatory activities [3,4,5].

**Scheme 1:**
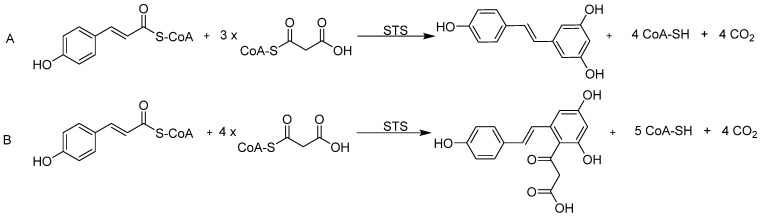
Enzymatic formation of (**A**) tetraketide and (**B**) 2-malonylresveratrol stilbene products by the stilbene synthase (STS) from *P. sylvestris* with 4-coumaroyl-CoA as a starter unit and malonyl-CoA as extender units.

Stilbene synthase (STS) is a plant type III polyketide synthase that catalyzes the first committed reaction in stilbenoids biosynthesis. STS is the key enzyme involved in the biosynthesis of the widely known natural product resveratrol with one molecule of coumaroyl-CoA and three molecules of malonyl-CoA (Scheme 1A). STS from Scots pine *(Pinus sylvestris*) also generates the previously unknown pentaketide, 2-malonylresveratrol, from four molecules of malonyl-CoA and one molecule of coumaroyl-CoA (Scheme 1B) [6]. STS also accepts various non-physiological substrates (mostly starter units) and efficiently generates unnatural novel products [7,8]. Resveratrol (trans-3,5, 4-trihydroxystilbene) is a phytoalexin produced by a number of plants. It was found in the skin of grapes, peanuts, blueberries, and knotweed, and also in some pines such as scots pine (*P. sylvestris*) and eastern white pine (*P. strobus*) [9,10]. Moreover, four novel derivatives of stilbenes have been isolated from the root bark of *Ekebergia benguelensis* (African species) with biological functions favorably against various panels of human cancer cell lines [11]. There have been great interests in the biological properties of resveratrol since it was demonstrated to interfere with all three stages of carcinogenesis (initiation, promotion, and progression) [12,13]. It was further reported that resveratrol can extend the survival rate and longevity of the yeast *Saccharomyces cerevisiae* by activating sirtuins, which adds an extra merit to its and related molecules’ growing list of biological properties [14]. Besides naturally occurring polyketides various intuitive and rational design of enzymes have been exploited to alter their enzymatic reactions, thereby expanding the diversity of products [15,16,17]. Some of novel unnatural polyketides products formed by structure-guided mutagenesis or obtained from the enzymatic conversion of non-physiological substrates have exhibited a promising perspective in drug discovery [18,19,20].

The very first STS (*Pinus sylyestris)* crystal structure was solved by the group of Joe Noel [21]. It is made up of an αβαβ core motif that is conserved among all type III polyketide synthases as well as enzymes containing a thiolase fold, such as ketoacyl thiolase. STS (and other PKSs-III) bears a striking resemblance, both in sequence and structure, to thiolase enzymes such as ketoacyl synthases and tholases, which provides evidence for the hypothesis that STS evolved from an enzyme of fatty acid biosynthesis [22]. In this study, the structure-guided engineering of benzoate-CoA ligase was practiced to generate a starter substrates library. The capability and capacity of the engineered benzoate-CoA ligase are manifested by crystal structures in complex with a 2-chloro-1,3-thiazole-5-carboxyl-AMP or 2-methlthiazole-5-carboxyl-AMP intermediate. We then took advantage of the substrate promiscuity of STS from *P. sylvestris*, which generated seven unnatural polyketide products. The biological effects of these synthesized compounds were evaluated against three different cancer cell lines (A549, Cal27, and HCT116), in which some demonstrated favorable cytotoxicity and apoptosis activities. 

## 2. Materials and Methods

### 2.1. Chemicals and Reagents

Malonic acid, 2-^13^C-malonic acid, 4-coumaric acid, 2-methylthiazole-5-carboxylic acid, resveratrol, *p*-coumaric acid, 7-phenyl heptanoic acid, 6-phenyl hexanoic acid, 5-(4-flurophenyl)valeric acid, 4-biphenylacetic acid, *N*-acetyl-L-cysteine, benzoic acid adenosine triphosphate (ATP), coenzyme A trilithium salt (CoA), magnesium chloride, dimethyl sulfoxide (DMSO) hexahydrate, phospho(enol) pyruvate potassium salt, PEG 4000, NaCl, potassium and sodium phosphate, IPTG, kanamycin, penicillin, streptomycin, were purchased from Sigma-Aldrich. 2-Chloro-1,3-thiazole-5-carboxylic acid and 4-methylthiazole-5-carboxylic acid were obtained from Matrix Scientific.

### 2.2. Bacterial Strains

*Escherichia coli* strains DH5α and BL21 (DE3) were used for DNA construction and heterologous protein expression, respectively. Pyruvate kinase (PykF) and myokinase (ADK) were heterologously expressed in *E. coli* (strain *K12*).

### 2.3. Site-Directed Mutagenesis

The benzoate-CoA ligase (BadA) double mutant (H333A/I334A) was constructed using the Quikchange Site-directed mutagenesis Kit (Stratagene) following the manufacturer’s protocol. First, point mutant was created on position H333A using the BadA wild type plasmid as a template. Second, the mutant (H333A) plasmid was used as a template to create a double mutant (H333A/I334A). All the mutants were confirmed by DNA sequencing. A pair of primers used to create the mutant are listed below: 

H333A,

sense 5′-GGTTCGACAGAAAGATGGCCAGCATCTCGGTCGAGC-3′

anti-sense 5′-GCTCGACCGAGATGCTGGCCATCTTTCTGTCGAACC-3′

I334A,

sense 5′-GCAGGTTCGACAGAAAGGCGTGCAGCATCTCGGTCG-3′

anti-sense 5′-CGACCGAGATGCTGCACGCCTTTCTGTCGAACCTGC-3′

### 2.4. Protein Expression and Purification

Plasmids of stilbene synthase (STS) were transformed into *E. coli* BL21 (DE3) and cultured in 1 L LB medium containing 50 mg/mL kanamycin at 37 °C until the A_600_ reached 0.6 and at which point cultures were cooled on ice for 30 min. Next, 0.2 mM isopropyl-d-thiogalactopyranoside (IPTG) was added to induce protein expression, and the culture was incubated at 16 °C for 16 h. The cells were harvested by centrifugation, resuspended in 40 mL binding buffer (50 mM potassium phosphate pH 7.4, 500 mM NaCl, 10 mM imidazole, 10% glycerol), and ruptured by sonication followed by centrifugation (20,000 rev min^−1^, 30 min, 4 °C) to remove the cell debris. Supernatants were loaded onto a Ni^2+^-NTA resin column that was pre-equilibrated with binding buffer. After washed with 50 mM potassium phosphate buffer, pH 7.5, 500 mM NaCl and 20 mM imidazole, the recombinant proteins were eluted with 50 mM potassium phosphate buffer, pH 7.5, 500 mM NaCl and 300 mM imidazole. All eluted proteins were further purified by size-exclusion chromatography on an ÄKTA FPLC system equipped with a HiLoad 16/60 Superdex 200 column under an isocratic condition (20 mM HEPES pH 8.0, 100 mM NaCl). Protein purity was examined by SDS-PAGE. Protein concentrations were estimated by the Bradford assay using BSA as a standard.

Malonyl-CoA synthetase from *Rhizobium triofli* (*Rt*MCS), benzoate CoA ligase from *Rhodopseudomonas palustris* (BadA), 4-coumarate-CoA ligase from *Populus tomentosa* (4CL) were individually purified using 100 mM sodium phosphate, 300 mM NaCl, 10% glycerol, 2.0 mM EDTA, 2 mM DTT, pH 7.5, and finally eluted using 300 mM imidazole. All the expression and purification procedures were the same as described above unless otherwise stated.

### 2.5. Enzyme Reaction

For stilbene synthase, the standard reaction mixture contained 30 nmol of CoA and 90 nmol of malonyl CoA and 300 pmol of the purified recombinant enzyme in a final volume of 200 µL of 100 mM potassium phosphate buffer pH 7.3 containing 1 mM EDTA. After incubation at 30 °C for 2 h, the reactions were stopped by addition of 20 µL of 6 N HCl. The reaction products were then extracted twice with 400 µL of ethyl acetate and were dried under SpeedVac (Thermo Scientific^TM^). The samples were re-dissolved in methanol (100 µL) and analyzed by HPLC and LC-ESI-MS with a Phenomenex Prodigy RP-18 column (250 mm × 4.6 mm, 5 µm). The mobile phase was set as: H_2_O with 1% trifluoroacetic acid in solvent A and MeCN with 1% trifluoroacetic acid in solvent B, a linear gradient from 2% to 60% of solvent B in solvent A at a flow rate of 1.0 mL min^−1^ over 31 mins, followed by 70% of solvent B for another 4 mins. The analytes were monitored under a UV wavelength at 280 nm using both positive and negative modes for mass detection. High-resolution mass spectra were analyzed with a Micromass Q-TOF instrument equipped with an ESI source. 

For large-scale enzyme reaction, 5 mmol of the purified enzyme was incubated with 20 mmol of starter-CoA and 60 mmol of malonyl-CoA in a 100 mL buffer solution (100 mM potassium phosphate, pH 7.3 containing 1 mM EDTA) at 30 °C for 10 h. The reaction was quenched by the addition of 6 N HCl and extracted with ethyl acetate (3 × 100 mL). The enzyme reaction products were purified and collected by the HPLC with a Phenomenex Prodigy HPLC column before the NMR analysis.

### 2.6. Synthesis of Starter-CoA Derivatives

All the starter-CoA (4-coumaroyl-CoA, 2-methylthiazole-5-carboxyl-CoA, 2-chloro-1,3-thiazole-5-carboxyl-CoA and 4-methylthiazole-5-carboxyl-CoA) derivatives used under this project were synthesized chemo-enzymatically. 4-Coumaroyl-CoA is synthesized as described earlier using 4-coumarate-CoA ligase [23]. Briefly, 880 µL of 100 mM Tris-HCl buffer pH 7.5 containing 5 mM Mg^2+^, 5 mM ATP, 1.5 mM CoA, 0.5 mM 4-coumaric acid, 400 pmol of purified enzymes and the reaction was incubated at room temperature for 12 h. The reaction was terminated by adding 100 µL of 6 N HCl and the reaction product was analyzed by HPLC and LC-ESI-MS installed with a Phenomenex Prodigy RP-18-column (250 mm × 4.6 mm, 5µm) with a flow rate of 1 mL/min.

Similarly, thiazole-CoA derivatives (2-methylthiazole-5-carboxyl-CoA, 2-chloro-1,3-thiazole-5-carboxyl-CoA and 4-methylthiazole-5-carboxyl-CoA) were enzymatically synthesized using benzoate-CoA ligase (BadA) double mutant (BadA-H333A/I334A). The reaction mixture contained 5 mM Mg^2+^, 5 mM ATP, 1.5 mM CoA, 0.5 mM substrate (2-methylthiazole-5-carboxylic acid, 4-methylthiazole-5-carboxylic acid, 2-chloro-1,3-thiazole-5-carboxylic acid), and 500 pmol of purified enzymes in a final volume of 1 mL was incubated at room temperature for 12 h. The reaction was quenched by the addition of 10% 6 N HCl and analyzed by HPLC and LC-ESIMS. As a representative, 4-methylthiazole-5-carboxyl-CoA structure was further illustrated by NMR (Table S1, Figures S5, S7–S11).

For large scale collection, the reaction was scaled up to 20 mL keeping all the reaction parameters constant as in starter-CoA derivatives (4-coumaroyl-CoA and for 2-methylthiazole-5-carboxyl-CoA, 4-methylthiazole-5-carboxyl-CoA, and 2-chloro-1,3-thiazole-5-carboxyl-CoA). The enzyme reaction products were purified by reverse-phase HPLC with a mobile phase set as: H_2_O with 1% trifluoroacetic acid in solvent A and MeCN with 1% trifluoroacetic acid in solvent B, a linear gradient from 10% to 20% of solvent B in solvent A at a flow rate of 1.0 mL min^−1^ for 5 mins, increased to 40% of solvent B in 24 mins and followed by 98% of solvent B for another 4 mins, then back to the initial condition. All the collected products were freeze-dried and stored at −80 °C for further use.

### 2.7. Synthesis of Acyl-NAC and Coenzyme-A Derivatives

For the synthesis of compounds **4**–**7**, acyl-NAC was first synthesized. Acyl-NAC was synthesized either by combining the acyl chloride directly with *N*-acetylcysteamine (NAC) (1.0 mmol) and trimethylamine (TEA) (2.0 mmol) in dimethylformamide (DMF) or adding the free acid diphenylphosphoryl azide and NAC in DMF/TEA. The reaction was quenched by adding aqueous NH_4_Cl and extracting twice with 5 mL ethyl acetate. The organic layer was dried and evaporated to give a white solid. The white residues were purified by silica gel chromatography (EA:hexane = 20:80) to yield pure acyl-NAC [24].

### 2.8. Synthesis of Malonyl-CoA and [2-^13^C]-malonyl-CoA

Malonyl-CoA was chemo-enzymatically prepared from malonate, CoA, and ATP using *Rt*MCS. In order to regenerate ATP, myokinase/pyruvate kinase/PEP system (Figure S1A,B) was used to avoid the high concentration of adenosine monophosphate (AMP) that might inhibit MCS [25]. The reaction mixture (20 mL) contained 100 mM sodium phosphate, pH 7.5, 5 mM Mg^2+^, 5 mM ATP, 2 mM CoA, 2 mM malonic acid, 5 mM phosphoenolpyruvate, 1 µM myokinase, 1.5 µM pyruvate kinase, and 6 µM MCS. The reaction was incubated at room temperature overnight. The reaction was stopped by the addition of 10% HCl (6 N) and the product was analyzed on HPLC and LC-ESIMS. Pure malonyl-CoA was collected using the same gradient as that of starter-CoA, and the products were stored at −80 °C after being freeze-dried for further use.

### 2.9. Kinetics Assay

Steady-state kinetic parameter of STS for the formation of seven new products were determined using malonyl-CoA (60 μM) as a substrate. The experiments were carried out in triplicate using five concentrations (ranging 1.5–20 μM) of seven starter CoA derivatives in a final volume of 200 μL of 100 mM potassium phosphate, (pH 7.4) 5 μg of the purified enzyme, 1 mM EDTA. The reactions were incubated at 30 °C for 2 h. 10% 6 N HCl was used to quench the reaction mixture at 0, 15, 30, 60, 90, and 120 min and the products were extracted using two volumes of ethyl-acetate and analyzed using HPLC (a Prodigy analytical C18 column was used with a flow rate of 1 mL min^−1^ and a linear gradient solvent system of increasing acetonitrile (0.1% FA) versus water (0.1% FA) over 30 min; UV was set at 230–310 nm). Prism version 7.04 (GraphPad Software) was used for data analysis and curve fitting.

### 2.10. NMR Analysis

NMR analysis was performed on the Bruker Avance 600 spectrometer equipped with CryoProbeTM. Compounds **1**–**7** were dissolved in deuterated dimethyl sulfoxide (DMSO-*d*6), of which the spectra were recorded at room temperature.

### 2.11. Crystallization and Data Collection

BadA mutant (H333A/I334A) at 10 mg/mL (in Tris-HCl, pH 7.5) was crystallized using the hanging drop vapor diffusion method in 1:1 ratio of protein and mother liquor (0.1 M NaCl, 0.2 M MES pH 6.5, PEG 4000) at 16 °C. Similarly, in order to obtain ligand-bound structures co-crystallization was performed with different components; ATP, CoA, 2-methylthiazole-4-carboxylic acid, 2-chloro-1,3-thiazole-5-carboxylic acid, and 4-methylthiazole-5-carboxylic acid were added separately into the H333A/I333A mutant protein solution. Each co-crystallization mixture was incubated for 30 min at 4 °C and followed by crystallization using mother liquor (0.1 M NaCl, 0.2 M MES pH 6.5, PEG 4000). Crystals were observed within 3-5 days. Crystals were soaked in cryoprotectant (20% glycerol and the same mother liquor) and flash-cooled in liquid nitrogen prior to X-ray data collection. All diffraction data were collected using ADSC Quantum 315 or MX300-HE CCD detectors at an operating temperature of 100 K on beamlines 13B1, 13C1, 15A1, and 05A at the National Synchrotron Radiation Research Center (NSRRC), Taiwan and on beamline 44XU at SPring-8, Japan. Data were indexed and scaled with the HKL-2000 package [26]. The H333A/I334A mutant crystals belonged to space group P12_1_1, with unit-cell parameters a = 59.1, b = 95.3, c = 94.9 Å and α = 90°, β = 105°, γ = 90°. The statistics of data collection are shown in Table 1.

### 2.12. Structure Determination and Refinement

BadA mutant (H333A/I334A) *apo* structures were solved by the molecular replacement method using wild type-BadA structure as the search model (4EAT) (Phenix was used for MR). The redundancy independent merging R factor (R_rim_) and the precision indicating merging R factor (R_pim_) were calculated using the program RMERGE. The contents of asymmetric units were estimated from the Matthews coefficient [27]. The Molprobity analysis was carried out by MolProbit [28]. Detailed refinement statistics are given in Table 1. The working model was further refined with Phenix. Figures were generate using PyMOL (http://www.pymol.org) molecular graphic system.

### 2.13. Cell Culture

All cell culture procedures were carried out aseptically in a First-Class microbiological safety cabinet with a laminar flow system. The safety cabinet was disinfected with 70% IMS in H_2_O prior to conduction of every experiment. Cell lines (A549, HCT116, Cal27) were routinely cultured in cell culture dishes (100 mm × 20 mm) in Dulbecco’s Modified Eagle Medium ((DMEM), Life Technology) culture media supplemented with 10% fetal bovine serum (FBS) (Invitrogen) and 1% penicillin/streptomycin (Invitrogen) under an atmosphere of 95% air and 5% CO_2_ at 37 °C. 

### 2.14. Cell Viability Assay

The Alamar Blue (AB) assay (Bio-Rad Laboratories), which includes a redox indicator that changes color or emits fluorescence in response to metabolic activity, was used to assess in vitro mammalian cell cytotoxicity (Carcinoma human alveolar basal epithelial cell line (A549), Human colorectal carcinoma cell line (HCT116), and Human tongue squamous cell carcinoma cell line (Cal27)) upon the compound treatment. Cells at a density of 5000 cells/well were seeded in 96-well plates overnight at 37 °C in a humidified incubator (5% CO_2_ atmosphere) followed by exposure to a series of concentrations of resveratrol or compounds **1**–**7**, (25, 50, 100, and 200 µM). Medium and 1% DMSO, which were used to dissolve compounds, served as a control. Cell viability was triplicated and assessed at 48 h for each testing compound, on which the measurements followed the manufacturer’s protocol.

### 2.15. Flow Cytometry Analysis

The Annexin V-FITC apoptosis detection kit (Strong Biotech Corporation) was used to detect cell apoptosis. First, A549 cells were seeded in six-well plates and treated with resveratrol or compound **7** with two different concentrations (50 μM or 100 μM). 1% DMSO was used as a negative control. After 48 h, cells were harvested and washed twice using precooled PBS buffer. Cells were then stained with Annexin V and propidium iodide (PI) according to manufacturer’s protocol.

## 3. Results

### 3.1. Structure-Based Engineering of Benzoate-Coenzyme A Ligase (BadA)

**Figure 1:**
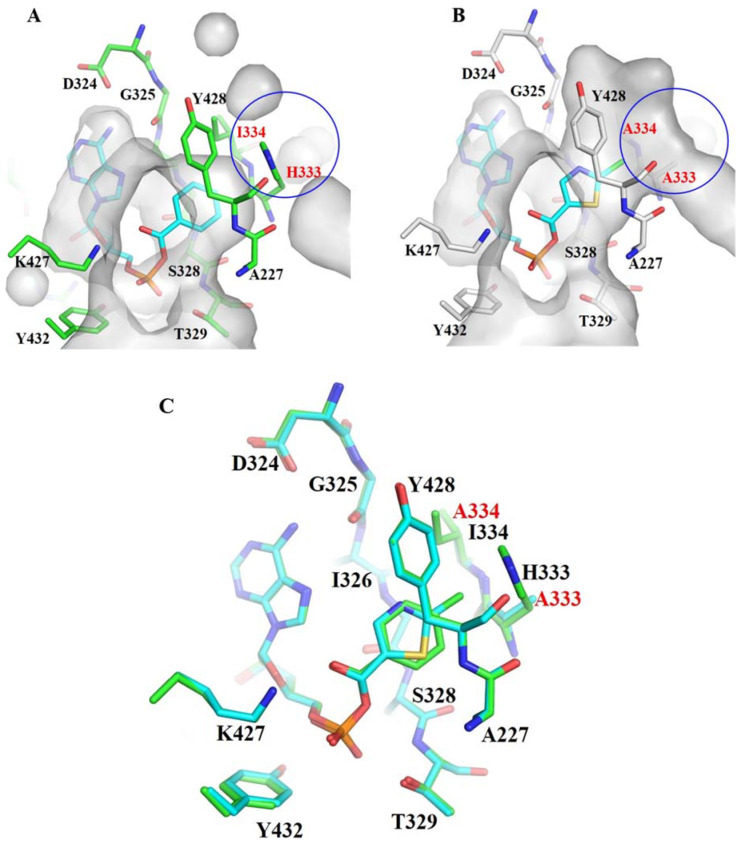
Surface representation of the active-site geography for BadA wild-type and mutant. (**A**) Wild-type BadA in complex with benzoyl-AMP, where two amino-acid residues His333 and Ile334 define the volume of the benzoate binding site. (**B**) The volume of the benzoate binding site is considerably increased when the two residues are changed to alanine (H333A and I333A in red inside the blue circle). (**C**) There is no significant conformational difference except the mutation residues H333A/I334A when wild-type BadA (green) was superposed on top of the mutant (cyan).

**Figure 2:**
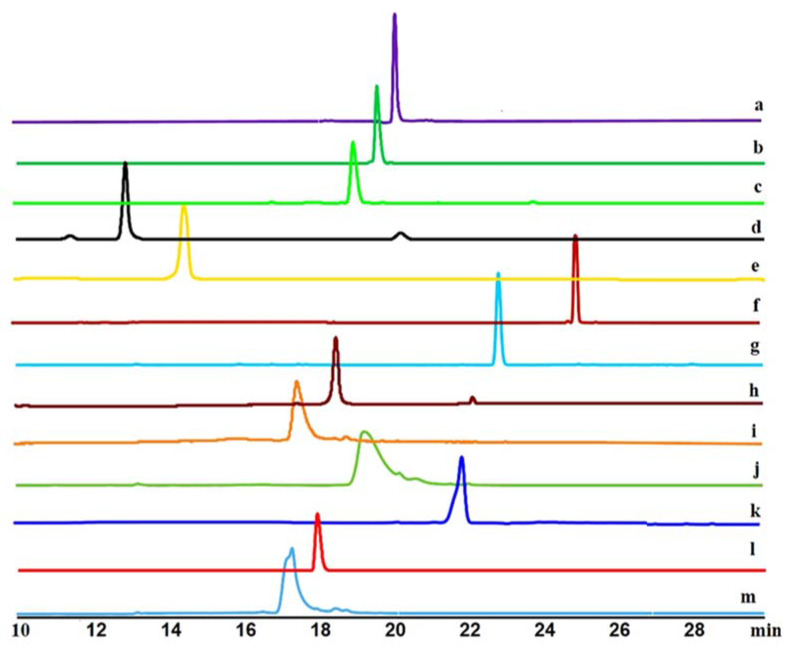
HPLC chromatograms of 13 different starter units synthesized by the BadA mutant. (a) p-coumaroyl-CoA, (b) dihydrocinnamoyl-CoA, (c) 3-(p-tolyl)propionyl-CoA, (d) 2-aminothiazole-4-carboxyl-CoA, (e) 2-aminothiazole-5-carboxyl-CoA, (f) 2-bromo-4-methylthiazole-5-carboxyl-CoA, (g) 2-bromo-5-methylthiazole-4-carboxyl-CoA, (h) 4-methylthiazole-5-carboxyl-CoA (i) 4-methylthiazole-2-carboxyl-CoA, (j) 2-chloro-1,3-thiazole-4-carboxyl-CoA, (k) 2-chloro-1,3-thiazole-5-carboxyl-CoA, (l) 2-methylthiazole-5-carboxyl-CoA, (m) 2-methylthiazole-4-carboxyl-CoA.

BadA catalyzes the conversion of benzoate to benzoyl-CoA in the metabolism of aromatic carboxylic acids. Its catalytic mechanism is shown as follows: benzoate firstly docks to the active site, where it orientates to allow the carboxylic group access to adenosine triphosphate (ATP) for the formation of an adenosine monophosphate (AMP) intermediate; coenzyme A (CoA) subsequently comes in to substitute the AMP-activated intermediate resulting in the formation of benzoyl-CoA. With the aim of generating a library of unusual starter substrates, structure-guided mutagenesis of BadA was pursued. During this effort, the BadA structures were independently determined by another group [29]. The solved structures show that the benzoate binding cavity is rather small and narrow, constituted by several bulky amino acid residues including Y228, G327, S328, T329, H333, and I334. With the purpose to allow acceptance of various bulkier substrates into the active site, a double mutant H333A and I334A was created (Figure 1). The mutant was examined against a series of carboxylic acids. To our surprise, most of the tested carboxylic acids can be taken by the mutant to form corresponding CoA products (Table 2, Figure 2, Figures S3 and S4) as opposed to the wild type that cannot accept them.

### 3.2. Crystal Structures of BadA-Mutant (H333A/I333A)

**Figure 3:**
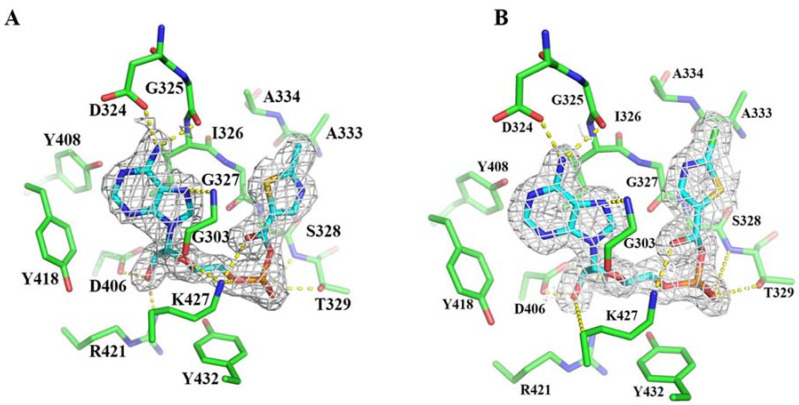
The binding site of BadA double mutant (H333A/I334A) in complex with ligands. (**A**) The crystal structure of the BadA double mutant in complex with 2-methylthiazole-5-carboxyl-AMP (PDB entry 6M2T). The electron density map for the ligand is shown in 2Fo–Fc weighted maps (gray). (**B**) The crystal structure of the BadA double mutant in complex with 2-chloro-1,3-thiazole-5-carboxyl-AMP (PDB entry 6M2U). The electron density map for the ligand is shown in 2Fo–Fc weighted maps (gray). The residues that interact with the ligand through hydrogen bonds are shown in yellow dotted lines. The electron density maps were countered at 1 σ.

To validate the above finding, we pursued X-ray crystallography. Three crystal structures of the double mutant H333A/I334A in *apo* (PDB entry 6M2O) or in complex with a 2-chloro-1,3-thiazole-5-carboxyl-AMP (2Cl5C-AMP) (PDB entry 6M2U) or 2-methylthiazole-5-carboxyl-AMP (2M5C-AMP) (PDB entry 6M2T) intermediate were obtained at resolutions of 1.70 Å, 1.57 Å, and 2.13 Å, respectively, for which the data collection and refinement statistics are summarized in Table 1. 2Cl5C-CoA, 2M5C-CoA, 4-methylthiazole-5-carboxyl-CoA (4M5C-CoA) and 2-bromo-4-methylthiazol-5-caroxyl-CoA were also co-crystallized with the mutant enzyme in a hope to obtain CoA bound structures, while we were unable to obtain these complexed structures. There is no significant conformational change between the wild type and the mutant as the root-mean-square deviation for the 394 Cα is about 0.3 Å. As revealed from the determined complexes, there are considerable hydrophobic contacts between substrates and the polypeptide backbone of the BadA active site. Superposition of these residues and ligands for the wild type and the mutant BadA reveals that the overall architecture is almost identical except that the volume of the substrate-binding pocket is increased by 96.7 Å^3^ in the mutant (Figure 1A–C respectively). The volume was calculated by using CASTp and visualized by Chimera [30,31]. In the ATP-dependent aryl CoA ligase family, most crystal structures in complex with their natural carboxylate substrates adopt the adenylation conformation, while few adopt CoA-bound thiolation conformation. For example, the crystal structure of BCLM (a benzoate CoA ligase in complex with benzoate; PDB entry 2V7B), which shares a 61% sequence identity with BadA, adopts the adenylation conformation [29,32,33]. In contrast, the wild type BadA structure (PDB entry 4EAT, solved by the group of Kevin Walker), as well as the mutant BadA structures (*apo*, 2M5C-AMP, and 2Cl5C-AMP), all adopt the thiolation conformation in the absence of CoA. In AMP binding site, Lys427 plays an important role in coordination with the carboxylate group of the ligand by its positive charge and in stabilization with the AMP group during the thioesterification (Figure 3A,B). Several residues in the AMP binding site, including Arg421, Thr329, Ser328, Gly303, Asp406, Asp324, Asp325, with polar contacts with the AMP-linked intermediate together stabilize the intermediate for subsequent thioesterification to proceed (Figure 3A,B).

### 3.3. Precursor-Directed Biosynthesis of Novel Polyketide Products

STS catalyzes the formation of resveratrol, which is constituted from one molecule of coumaroyl-CoA and three units of malonyl-CoA; the same STS also generates 2-malonylresveratrol, which is made of one molecule of coumaroyl-CoA and four units of malonyl-CoA (Scheme 1B). This fact, however, revealed that the enzyme does not faithfully control the number of condensations, thereby often giving rise to new products [6]. 

**Figure 4:**
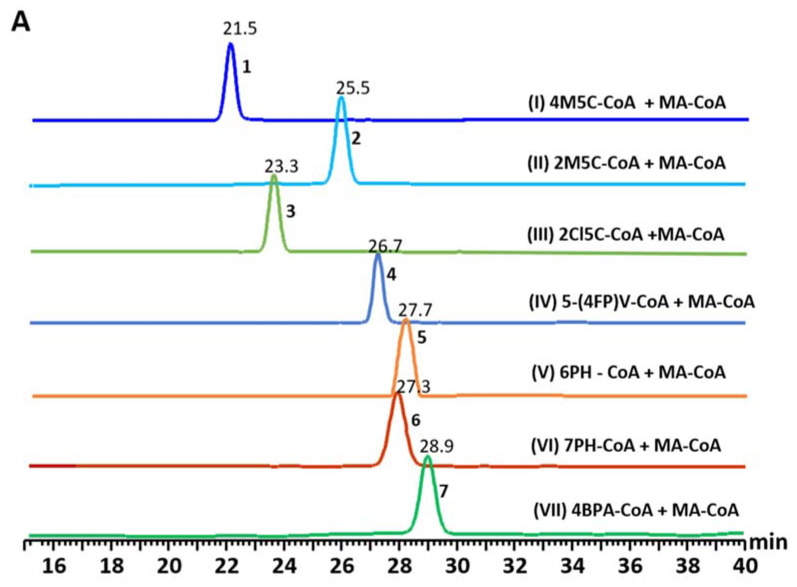

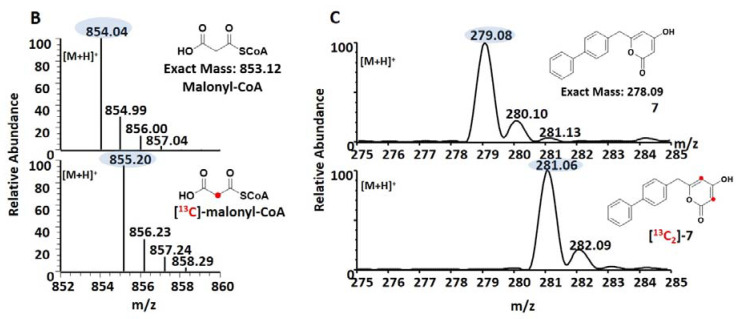
HPLC chromatographs and mass spectra. (**A**) LC traces of enzymatic reactions catalyzed by STS in the presence of unnatural starters along with malonyl-CoA. Trace (I): a product peak (**1**) emerged at 21.5 min when the reaction added with STS in the presence of 4-methylthiazole-5-carboxyl-CoA (4M5C-CoA) and malonyl-CoA (MA-CoA), trace (II): a product peak (**2**) emerged at 25.5 min when the reaction added with STS in the presence of 2-methylthiazole-5-carboxyl-CoA (2M5C-CoA) and MA-CoA, trace (III): a products peak (**3**) emerged at 23.3 min when the reaction added with STS in the presence of 2-chloro-1,3-thiazole-5-carboxyl-CoA (2Cl5C-CoA) and MA-CoA, trace (IV): a product peak (**4**) eluted at 26.7 when 5-(4-fluorophenyl)valeriyol-CoA [5(4FP)V-CoA] and MA-CoA incubated together with STS, trace (V): a product peak (**5**) emerged at 27.7 min when 6-phenylhexanoyl-CoA (6PH-CoA) and MA-CoA reaction mixture added together with STS, trace (VI): a product peak (**6**) generated at 27.3 min when 7-phenylheptanoyl-CoA (7PH-CoA) and MA-CoA incubated together with STS, trace (VII): a product peak (**7**) emerged at 28.9 min when 4-biphenylacetyol-CoA (4BPA-CoA) and MA-CoA was incubated together with STS. (**B**) Mass spectra of malonyl-CoA and ^13^C-malonyl-CoA (enzymatically synthesized by *Rt*MCS), (**C**) Mass spectra of compound **7** and ^13^C_2_-compound **7** isotope labeling. Likewise, all compounds were confirmed using ^13^C-malonyl-CoA.

To further explore/expand the substrate promiscuity of *P. sylvestris* STS, five different thaizole CoA derivatives 4-methylthiazole-5-carboxyl-CoA, 2-methylthiazole-5-carboxyl-CoA, 2-chloro-1,3-thiazole-5-carboxyl-CoA, 2-bromothiazole-4-methyl-5-carboxyl-CoA (2Br4M5C-CoA), 2-bromothiazole-5-methyl-4-carboxyl-CoA (2Br5M4C-CoA) were enzymatically synthesized using the engineered BadA, which were then subjected to biochemical examination. Each of these thiazole CoAs and malonyl-CoA were incubated under the same enzymatic reaction condition, in which the reaction added with 4-methylthiazole-5-carboxyl-CoA, 2-methylthiazole-5-carboxyl-CoA, or 2-chloro-1,3-thiazole-5-carboxyl-CoA yielded a new peak (Figure 4A, **1**, **2**, **3**). These products all gave a maximum UV absorbance (λ_max_) at 310 nm with a specific mass unit [M + H]^+^ at 210.0225, 210.0218, and 229.9681 *m/z*, respectively (Table 3), suggesting a triketide nature of two sequential condensation events with two molecules of malonyl-CoA attached to a given starter unit. All these three products were confirmed by NMR (Supporting Information SI, Tables S2 and S3, Figures S12–S43). The enzyme kinetics showed the apparent *K*_m_: 3.82 µm, *k*_cat_: 0.16 min^−1^, *K*_m_: 3.87 µm, *k*_cat_: 0.016 min^−1^, and *K*_m_: 12.77 µm *k*_cat_: 0.19 min^−1^, respectively, for the formation of products **1**–**3** (Table 3). In contrast, 2Br4M5C-CoA and 2Br5M4C-CoA did not yield any new peaks when the reactions added with STS in the presence of malonyl-CoA. All these newly emerged peaks were double confirmed by using 2-^13^C-malonyl-CoA (Figure 4B,C). Stable isotope labeling using ^13^C-malonyl-CoA is conducive to the determination of the number of extender units incorporated into the final compound and/or cyclized to form a specific aromatic structure. The mass spectra of compound **7** and ^13^C_2_-compound **7** are shown in Figure 4C as a representative example.

**Figure 5:**
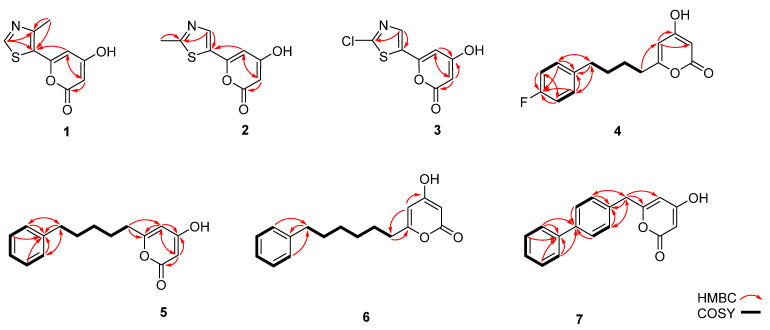
Chemical structures and 2D-NMR (HMBC and COSY) correlations of compounds **1**–**7**. COSY correlations were presented in bold lines; HMBC correlations were showed in red arrows.

In addition to enzymatic synthesis, compounds **4**–**7** were chemoenzymatically prepared. To make them, acyl-NAC was first synthesized and substituted by in situ transthioesterification with CoA to form acyl-CoA (details in methods and materials section). The CoA products were purified to homogeneity and then subjected to STS in vitro biosynthesis. When 5-(4-flurophenyl)valeriyl-CoA was examined, a new peak was found in HPLC trace and determined to be a fluorinated pyrone derivative (**4**) (Figure 4A, IV, Table 3). Compound **4** gave a maximum UV absorbance (λ_max_) at 280 nm with a unique mass unit [M + H]^+^ at *m/z* 263.1084. Kinetics analysis rendered the apparent *K*_m_ 4.15 µM and *k*_cat_ 0.49 min^−1^ for compound **4**. Similarly, *P. sylvestris* STS also accepts 6-phenylhexanoyl-CoA, 7-phenylheptanoyl-CoA, or 4-biphenylacetyol-CoA to produce corresponding polyketide. The LC-HRMS analysis of **5** and **6** gave parent ion mass units [M + H]^+^ at 259.1340 and 273.1475 *m/z*, respectively (Figure 4A, V and VI, Table 3). In a similar manner, given 4-biphenlacetyol-CoA a new peak (**7**) emerged with a parent ion peak [M + H]^+^ at 279.1012 *m/z* and a maximum UV absorbance (λ_max_) at 254 nm. Kinetics analysis rendered the apparent *K*_m_ 23.24 µm and *k*_cat_ 0.21 min^−1^ for compound **7**. The chemical structures of compounds **1**–**7** were determined spectroscopically by both 1D and 2D NMR (Figure 5, SI Figures S6, S12–S43)

### 3.4. Cell Viability

**Figure 6:**
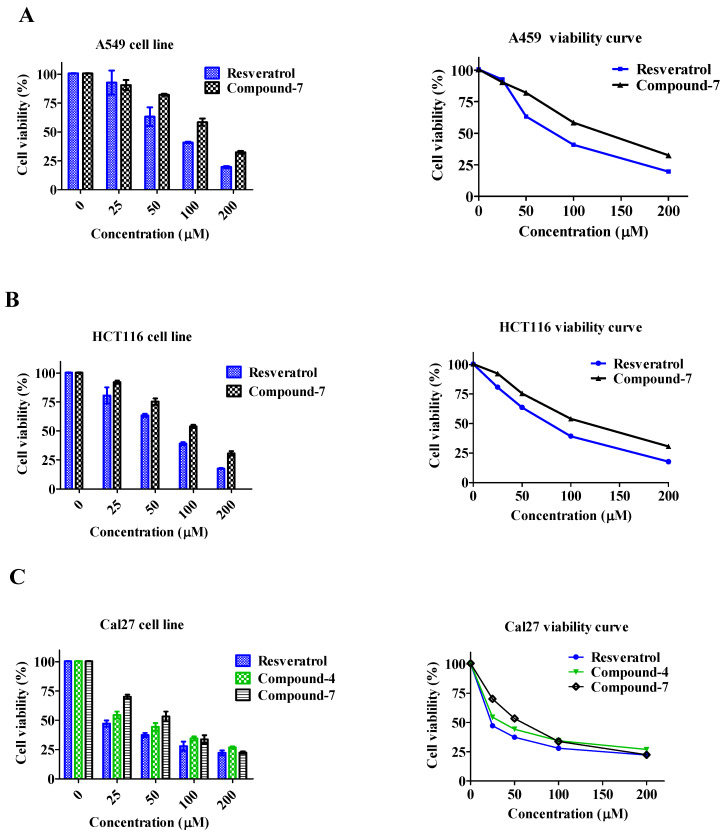
Effects of compounds on cell viability. Cell viability of A549 (**A**), HCT116 (**B**), Cal27 (**C**) cell lines after treated with different doses of compounds (resveratrol) or compound **4** or compound **7** for 48 h. The dose-dependent viability curves are shown for each cell line. Errors bars are mean with standard deviations.

It has been well documented that resveratrol and some of its derivatives exhibit such remarkable properties as anti-inflammatory, antiaging, antibacterial and/or antitumor activities [3,4,12]. Recently, four novel stilbene products isolated from the rootbark of *Ekebergia benguelensis* once again underscored their cytotoxicity effect favorably against various panels of cancer cell lines [11]. To know whether the newly synthesized compounds also possess similar or better activity, we examined their effects on cell viability and apoptosis. First, compounds **1**–**7** were individually assayed for their anticancer activity against cancer cell lines A549, Cal27, and HCT116 (Figure 6), in which resveratrol and 1% DMSO respectively serve as positive and negative control. As a result, compounds **1**–**3** and **5**–**6** show no significant cytotoxicity against cancer cell lines A549, HCT116, and Cal27 (data not shown); compounds **4** and **7**, however, exhibited a relatively strong cytotoxic effect. For A549 cells, although compound **7** shows no significant effect at a low concentration (25 μM) at 48 h, the cell inhibitory effect (IC_50_ = 65.45 μM at 48 h) becomes significant in reference to resveratrol (IC_50_ = 52.34 μM) when the concentration was elevated above 50 μM (Figure 6A). For HCT116 cells, the IC_50_ of compound **7** was estimated to be 61.87 μM at 48 h on the basis of the dose-dependent viability curve, which again is commensurate with resveratrol (IC_50_ 49.56 μM) (Figure 6B). For Cal27 cells, compounds **4** and **7** likewise showed a significant cell-growth inhibition effect (Figure 6C). The cell viability decreases with increase of the dose, where the cell viabilities were estimated around 30%, 40%, and 40% for the groups treated with resveratrol (IC_50_ = 14.45 μM), compound **4** (IC_50_ = 20.57), and compound **7** (IC_50_ = 32.02 μM) at 100 μM, respectively.

### 3.5. Cell Apoptosis

Apoptosis is characterized by specific morphological and biochemical changes, such as cell shrinkage, chromatin degradation, DNA fragmentation, and cell surface changes, for which flow cytometry analysis in couple with Annexin V-FITC and propidium iodide (PI) staining was often used to measure the extent of apoptoic cell death. Loss of plasma membrane is one of the earliest features in apoptosis. The fact is that in the presence of Ca^2+^ ions Annexin V binds to negatively charged phospholipids, phosphatidylserine (PS), on the membranes of cells with high affinity when cells undergoing apoptosis. The PI, on the other hand, can differentiate between early and late apoptotic cells because necrotic cells that have lost cell membrane integrity will permit PI entry. Therefore, viable cells are Annexin V negative/PI negative, early apoptotic cells are Annexin V positive/PI negative, late apoptotic or necrotic cells are Annexin V positive/PI positive, and early necrotic cells are Annexin V negative/PI positive.

**Figure 7:**
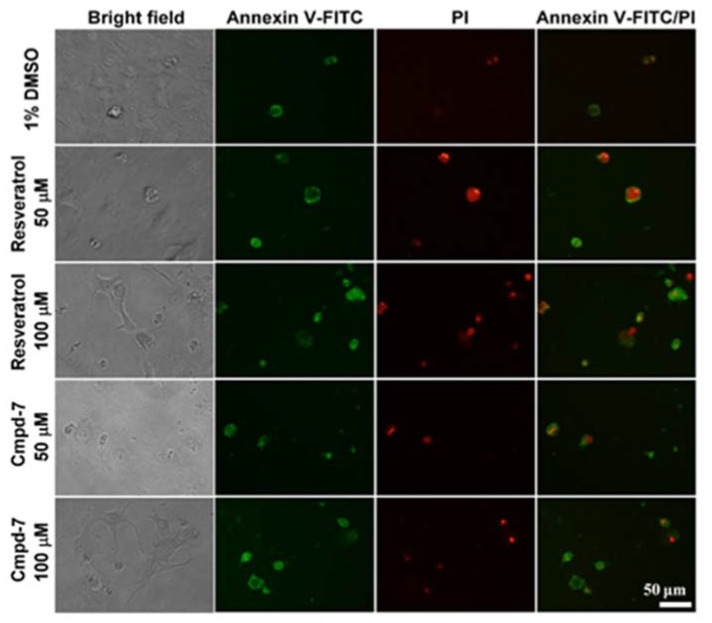
Effects of testing compounds on morphology of A549 cells. Cells were treated with resveratrol (50 or 100 μM) or compound **7** (Cmpd-***7***) (50 or 100 μM) for 48 h. Cell morphological changes were recorded under a bright field microscope. Cell viability was stained with Annexin V-FITC/ propidium iodide (PI) and observed under the fluorescent microscope. Green spots represent Annexin V-FITC positive cells, and red spots stand for PI positive cells. Magnification: 200×.

**Figure 8:**
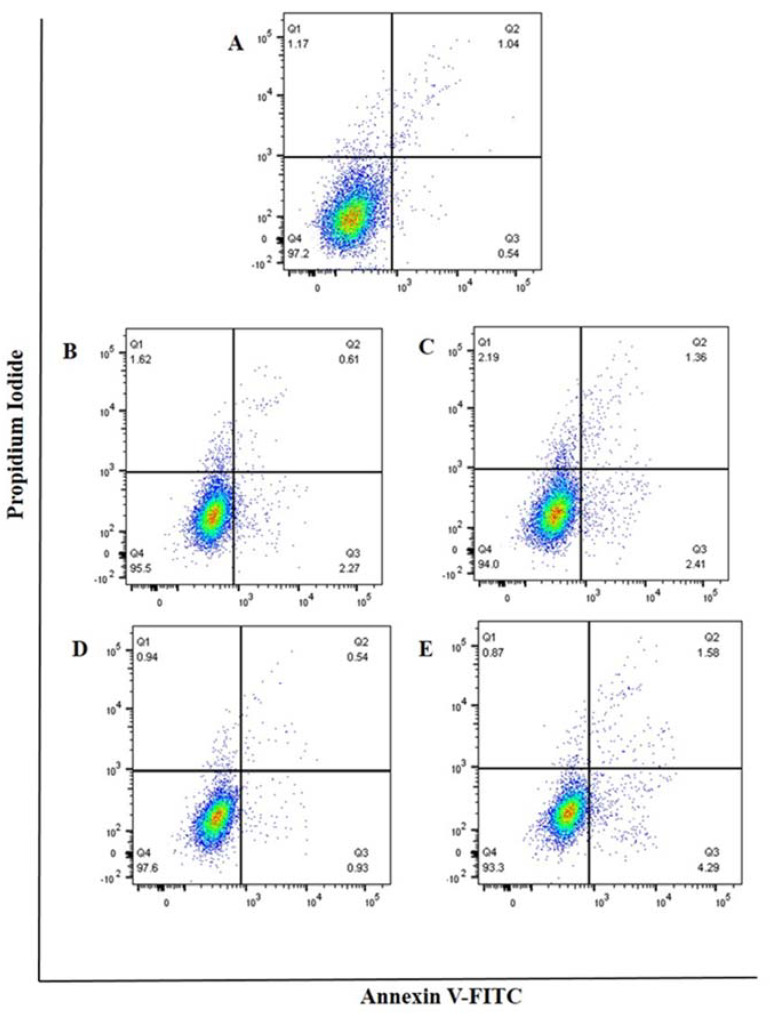
The extent of apoptosis of A549 cells with or without treatment was analyzed by flow cytometry. (**A**–**E**) Apoptosis-stage diagram of A549 cells treated with different concentrations of a given compound (50 or 100 μM) at 48 h. (**A**) 1% DMSO, (**B**,**C**) 50 or 100 μM resveratrol, respectively. (**D**,**E**) 50 or 100 μM compound 7, respectively. Cells in Q1, Q2, Q3, and Q4 quadrants represent necrosis, late apoptosis, early apoptosis, and normal cells, respectively.

With the treatment of resveratrol or compound **7**, the morphology of A549 was changed under the field fluorescent microscope (Figure 7). The cell becomes swollen and shows blurred edges, disrupted membranes, and decreased density; the contact between cells becomes smaller. In comparison with control (without drugs treatment), the inhibition of adherent growth can be observed, and cells were unable to proliferate, thus promoting the death of A549 cells (Figure 7, bright field). When A549 cells seeded in six-well plates were treated with two different doses (50 or 100 μM) of resveratrol or compound **7**, the cells stained with Annexin V-FITC or PI only or Annexin V-FITC/PI were examined under a fluorescent microscope (Figure 7, Annexin V-FITC and PI, Green spots represent Annexin V-FITC positive cells, and red spots stand for PI positive cells) or subjected to flow cytometry analysis. The four-quadrant diagram from the flow cytometry analysis was drawn to distinguish necrotic cells (Q1), late apoptosis (Q2), early apoptosis (Q3), and normal cells (Q4). In general, the ratio of normal cells decreased gradually, and the proportion of early and late apoptotic cells increased in the groups treated with resveratrol or compound **7** when compared to that of the control group (1% DMSO and cell). In the section of 50 μM treatment, most cells in the group receiving resveratrol show both early apoptosis and necrosis with the latter relatively dominant, while compound **7** shows apoptosis/necrosis in an equal ratio. This phenomenon is further contrasted in the cells treated with a high dose of resveratrol and compound **7**. Namely, when the cells received 100 μM of resveratrol or compound **7**, the early apoptosis and late apoptosis were increased by 4.29% and 1.58%, respectively, in the group treated with compound **7** (Figure 8D,E) as opposed to that treated with resveratrol, where late apoptosis and necrosis cell were increased by 1.36% and 2.19% (Figure 8B,C). The percentage of early apoptosis is higher for the cells treated with compound **7** than that treated with resveratrol, which relatively favors cells toward necrosis. This result accounts for compound **7** that gives rise to cancer cell cytotoxicity is due to apoptotic cell death.

## 4. Discussion

BadA that underwent structure-guided protein engineering has clearly exhibited an additional dimension on substrate tolerance in light of 13 novel carboxyl CoA derivatives synthesized herein (Table 2, Figure 2, Figures S3 and S4). The fact is that the benzoate binding site was small and limited not allowing acceptance of bulkier subtracts other than benzoate. The crystal structures of the BadA mutant (H333A/I334A) in complex with two different intermediates validated the objectivity and rationality of this modification. The new capability was validated by production of several non-physiological aryl CoA thioesters for advanced utilizations, for example, as building units in drug discovery. By the same token, we took advantage of *P. sylvestris* STS, which has been shown with broad substrate tolerance to some non-physiological substrates. STS, as expected, is capable of assimilating these newly synthesized CoA thioesters along with malonyl-CoA to form a group of unnatural polyketide scaffolds. STS, however, did not yield the stilbene-type product but instead a pyrone-type product. All reactions were terminated after two rounds of condensation reactions ending up as a tri-ketide derivative. This is likely due to a different spatial orientation for a non-physiological starter in reference to that for the natural one in the active site, thereby limiting the number of elongation reactions. On the other hand, fluorinated drug has recently attracted a great attention due to its peculiar pharmacological characteristics in drug development. For example, incorporation of fluorine in drugs can increase bioavailability, decrease clearance, enhance metabolic activity and stability, alter pKs and lipophilicity, or increase potency [34,35,36,37,38]. So far, there are only a few examples demonstrated in type I and type II PKS systems [25,39,40,41]. PKSs III, in particular, have not been thoroughly examined for their capacity to render novel halogenated bioactive compounds. 

Previous studies reported that resveratrol is in a position to inhibit proliferation of A549, Cal27, or HCT116 cancer cells as well as the growth of some types of tumors in vivo [42,43,44]. In view of the biological activity, compounds **4** and **7** effect medium cytotoxicity on selected cancer cell lines. Though the compounds are not better than resveratrol in terms of the overall cancer-cell killing effect, they do however display a distinctive favorable perspective in addition to being new chemical entities. Namely, the cancer-cell cytotoxicity of compound **7** overtakes resveratrol in terms of programmed cell death. We thus believe that further modifications of the compounds created herein are positioned to better exert their biological efficacy.

## 5. Conclusions

In conclusion, the engineered BadA has considerably expanded its product capacity in light of a series of unusual CoA derivatives synthesized herein. Three protein crystal structures in an *apo* form and in complex with a 2-methylthiazole-5-carboxyl-AMP or 2-choloro-1,3-thiazole-5-carboxyl-AMP intermediate validated the engineering rationality and synthetic practicality of the modified enzyme. Following the precursor-directed biosynthesis, seven structurally different polyketides were synthesized using the type III PKS stilbene synthase, of which the chemical structures were spectroscopically proven to be novel. Of them, compounds **4** and **7** exhibit appropriate cytotoxicity capable of driving a panel of testing cancer cells (A549, HCT116, Cal27) toward cell apoptosis. These results corroborate the approach used here, whereby the novel polyketides underscore promising biomedical features, thus greeting a chance for advanced development.

## Figures and Tables

**Table 1:** Data collection and refinement statistics for the crystal structures of H333A/I334A-*apo*, H333A/I334A-2Cl5C-AMP, and H333A/I334A-2M5C-AMP.

	H333A/I334A-Apo	H333A/I334A-2Cl5C-AMP	H333A/I334A-2M5C-AMP
**PDB code**	6M2O	6M2U	6M2T
**Wave length (Å)**	1.0	1.0	1.0
**Space group**	P 1 2_1_ 1	P 1 2_1_ 1	P 1 2_1_ 1
***a*,*b*, *c* (Å)**	59.1,95.3, 94.9	58.5, 94.6, 94.3	98, 95.1, 119.5
**α, β, γ (°)**	90, 105, 90	90, 104.9, 90	90, 110.5, 90
**Resolution range (Å) ^a^**	29.52–1.57(1.63–1.57)	29.8–1.70 (1.77–1.70)	27.7–2.13 (2.22–2.13)
***R*_merge_ (%) ^a,b^**	2.8 (36.5)	6.8 (73.2)	5.1 (20.1)
**〈*I*/σ(*I*)〉 ^a^**	31.6(3.1)	33 (2.8)	25.3 (6.6)
**Completeness (%) ^a^**	99.1 (98.3)	99.9(99.9)	100 (100.0)
**Redundancy**	3.8(3.8)	7.4 (6.7)	3.8 (3.8)
**Refinement**			
**Resolution range (Å) ^a^**	29.52–1.57 (1.57–1.52)	29.8–1.70 (1.77–1.70)	27.7–2.13 (2.22–2.13)
**R_work_/R_free_ (%)**	18.7/21.1	17/20.2	15.9/19.7
**R.m.s. deviations**			
**Bond lengths (Å)**	0.187	0.171	0.160
**Bond angles (°)**	1.43	1.89	1.77
**No. reflections**	140471	105468	113090
**No. atoms**			
**Protein**	1036	1036	2072
**Ligand/ion**		62	124
**Water**	1180	1307	1421
**B-factors**			
**Protein**	27.8	19.15	30.38
**Ligand/ion**		22.4	26.28
**Water**	27.9	29.3	30.7

^a^ Values in parentheses are for the highest resolution shell. ^b^ R_merge_ = Σ|I_i_-<I_i_>|ΣI_i_, I_i_ is the average intensity value of the equivalent reflections. 2Cl5C-AMP: 2-chloro-1,3-thiazole-5-carboxyl-AMP, 2M5C: 2-methylthiazole-5-carboxyl-AMP.

**Table 2:** Summary of selected carboxylates examined in the presence of the benzoate coenzyme A ligase (BadA) double mutant or the wild type. (+) sign indicates the mutant can recognize the acid to form corresponding carboxyl-CoA derivatives, while (-) indicates that the wild-type cannot utilize the acids.

Compounds	BadA Mutant (H333A/I334A)	Wild-Type BadA
4-methylthiazole-2-carboxylic acid	**+**	**-**
4-methylthiazole-5-carboxylic acid	**+**	**-**
2-methylthiazole-4-carboxylic acid	**+**	**-**
2-methylthiazole-5-carboxylic acid	**+**	**-**
2-chloro-1,3-thiazole-5-carboxylic acid	**+**	**-**
2-chloro-1,3-thiazole-4-carboxylic acid	**+**	**-**
2-bromo-4-methylthiazole-5-carboxylic acid	**+**	**-**
2-bromo-5-methylthiazole-4-carboxylic acid	**+**	**-**
2-aminothiazole-4-carboxylic acid	**+**	**-**
2-aminothiazole-5-carboxylic acid	**+**	**-**
3-(*p*-tolyl)propionic acid	**+**	**-**
dihydrocinnamic acid	**+**	**-**
*p*-coumaric acid	**+**	**-**

**Table 3:** Chemo-physical properties for compounds **1**–**7** synthesized by STS *P. Sylvestris*. The Mass and UV maxima absorbance (nm) were determined by HPLC-PDA-ESI-HRQTOF. Prism version 7.04 (GraphPad Software) was used for data analysis and curve fitting to derive kinetic parameters (apparent *K*_m_ and *k*_cat_). Mass spectra are shown in Figure S2.

Starter Unit	Product	Product Mass [M + H]^+^	Kinetics		UV_Max_ (nm)
			*K*_m_ (μm)	*k_cat_* (min^−1^)	
4-methylthiazole-5-carboxyl-CoA	**1**	210.0218	3.82	0.16	310
2-methylthiazole-5-carboxyl-CoA	**2**	210.0225	3.87	0.01	310
2-choloro-1,3-thiazole-5-carboxyl-CoA	**3**	229.9681	12.77	0.19	310
5-(4-fluorophenyl) valeriyol-CoA	**4**	263.1084	4.15	0. 40	280
6-phenylhexanoyl-CoA	**5**	259.134	43.89	0. 40	285
7-phenylheptanoyl-CoA	**6**	243.1475	29.42	0.27	285
4-biphenylacetyol-CoA	**7**	279.1012	23.24	0.21	254

## Supplementary Materials

The following are available online at https://www.mdpi.com/2218-273X/10/5/738/s1, Table S1: ^1^H (600MHz) and ^13^C (150 MHz) NMR data of 4M5C-CoA, Table S2: ^1^H-NMR data of 1-7 in DMSO-d_6_^a^, Table 3: ^13^C-NMR data of 1-7a in DMSO-d_6_^a,^ Figure S1: LC trace of malonyl-CoA derivatives and cyclic ATP regenerating system, Figure S2: HPLC-PDA-HRQTOF-ESI/MS spectra of the newly synthesized polyketides products, Figure S3: Chemical structure of carboxylic acids used as a substrate for BadA mutant (H333A/I334A) to synthesized the CoA derivatives, Figure S4: Mass spectrum [M + H]^+^ of representative CoA derivatives, Figure S5: The COSY and HMBC correlation of 4M5C-CoA, Figure S6: The assignment, physic data, 2D-NMR (HMBC and COSY) correlations of 1‒7, Figure S7: ^1^H NMR of 4M5C-CoA in D_2_O (600 MHz), Figure S8: ^13^C NMR of 4M5C-CoA in D_2_O (150 MHz), Figure S9: COSY spectrum of 4M5C-CoA in D_2_O, Figure S10: HSQC spectrum of 4M5C-CoA in D_2_O, Figure S11: HMBC spectrum of 4M5C-CoA in D_2_O, Figure S12: ^1^H NMR of compound 1 in DMSO-d_6_ (600 MHz), Figure S13: ^13^C NMR of compound 1 in DMSO-d_6_ (150 MHz), Figure S14: HSQC spectrum of compound 1 in DMSO-d_6_, Figure S15: HMBC spectrum of compound 1 in DMSO-d_6_, Figure S16: ^1^H NMR of compound 2 in DMSO-d_6_ (600 MHz), Figure S17: ^13^C NMR of compound 2 in DMSO-d_6_ (150 MHz), Figure S18: HSQC spectrum of compound 2 in DMSO-d_6_, Figure S19: HMBC spectrum of compound 2 in DMSO-d_6_, Figure S20: ^1^H NMR of compound 3 in DMSO-d_6_ (600 MHz), Figure S21: ^13^C NMR of compound 3 in DMSO-d_6_ (150 MHz), Figure S22: HSQC spectrum of compound 3 in DMSO-d_6_, Figure S23: HMBC spectrum of compound 3 in DMSO-d_6_, Figure S24: ^1^H NMR of compound 4 in DMSO-d_6_ (600 MHz), Figure S25: ^13^C NMR of compound 4 in DMSO-d_6_ (150 MHz), Figure S26: COSY spectrum of compound 4 in DMSO-d_6_, Figure S27: HSQC spectrum of compound 4 in DMSO-d_6_, Figure S28: HMBC spectrum of compound 4 in DMSO-d_6_, Figure S29: ^1^H NMR of compound 5 in DMSO-d_6_ (600 MHz), Figure S30: ^13^C NMR of compound 5 in DMSO-d_6_ (150 MHz), Figure S31: COSY spectrum of compound 5 in DMSO-d_6_, Figure S32: HSQC spectrum of compound 5 in DMSO-d_6_, Figure S33: HMBC spectrum of compound 5 in DMSO-d_6_, Figure S34: ^1^H NMR of compound 6 in DMSO-d_6_ (600 MHz), Figure S35: ^13^C NMR of compound 6 in DMSO-d_6_ (150 MHz), Figure S36: COSY spectrum of compound 6 in DMSO-d_6_, Figure S37: HSQC spectrum of compound 6 in DMSO-d_6_, Figure S38: HMBC spectrum of compound 6 in DMSO-d_6_, Figure S39: ^1^H NMR of compound 7 in DMSO-d_6_ (600 MHz), Figure S40: ^13^C NMR of compound 7 in DMSO-d_6_ (150 MHz), Figure S41: COSY spectrum of compound 7 in DMSO-d_6_, Figure S42: HSQC spectrum of compound 7 in DMSO-d_6_, Figure S43: HMBC spectrum of compound 7 in DMSO-d_6._
