# Supplementary material for: Chemoenzymatic Synthesis and Biological Evaluation for Bioactive Molecules Derived from Bacterial Benzoyl Coenzyme A Ligase and Plant Type III Polyketide Synthase
Source: Biomolecules. 2020 May 9;10(5):738. doi: 10.3390/biom10050738 (PMC7277991; doi:10.3390/biom10050738)
Supplement: Supplementary file 1 [file biomolecules-10-00738-s001.pdf]

Supporting Information

Chemoenzymatic Synthesis and Biological Evaluation for Bioactive Molecules Derived from Bacterial Benzoyl Coenzyme A Ligase and Plant Type III Polyketide Synthase

Kamal Adhikari, I-Wen Lo, Chun-Liang Chen, Yung-Lin Wang, Kuan-Hung Lin, Saeid MalekZadeh, Rajesh Rattinam, Yi-Shan Li, Chang-Jer Wu and Tsung-Lin Li

Contents

Table S1: ^{1}H (600MHz) and ^{13}C (150 MHz) NMR data of **4M5C-CoA**......................4
Table S2: ^{1}H-NMR data of **1–7** in DMSO-d_6 ^{a}..5
Table S3: ^{13}C-NMR data of **1–7**^{a} in DMSO-d_6^{a}...6
Figure S1: LC trace of malonyl-CoA derivatives and cyclic ATP regenerating system. 7
Figure S2: HPLC-PDA-HRQTOF-ESI/MS spectra of the newly synthesized polyketides products..9
Figure S3: Chemical structure of carboxylic acids used as a substrate for BadA-mutant (H333A/I334A) to synthesized the CoA derivatives..9
Figure S4: Mass spectrum [M+H]+ of representative CoA derivatives.....................12
Figure S5: The COSY and HMBC correlation of **4M5C-CoA**................................13
Figure S6: The assignment, physic data, 2D-NMR (HMBC and COSY) correlations of **1–7**.. 13
Figure S7. ^{1}H NMR of **4M5C-CoA** in D_2O (600 MHz)..14
Figure S8: ^{13}C NMR of **4M5C-CoA** in D_2O (150 MHz)..14
Figure S9: COSY spectrum of **4M5C-CoA** in D_2O 15
Figure S10: HSQC spectrum of **4M5C-CoA** in D_2O 15
Figure S11. HMBC spectrum of **4M5C-CoA** in D_2O..16
Figure S12: ^{1}H NMR of compound **1** in DMSO-d_6 (600 MHz)...............................16
Figure S13: ^{13}C NMR of compound **1** in DMSO-d_6 (150 MHz)..............................17
Figure S14: HSQC spectrum of compound **1** in DMSO-d_6 17
Figure S15: HMBC spectrum of compound **1** in DMSO-d_6 18
Figure S16: ^{1}H NMR of compound **2** in DMSO-d_6 (600 MHz)...............................18
Figure S17: ^{13}C NMR of compound **2** in DMSO-d_6 (150 MHz)..............................19
Figure S18: HSQC spectrum of compound **2** in DMSO-d_6 19
Figure S19: HMBC spectrum of compound **2** in DMSO-d_6 20
Figure S20: ^{1}H NMR of compound **3** in DMSO-d_6 (600 MHz)...............................20
Figure S21:^{13}C NMR of compound **3** in DMSO-d_6 (150 MHz)...............................21
Figure S22: HSQC spectrum of compound **3** in DMSO-d_6 21
Figure S23: HMBC spectrum of compound **3** in DMSO-d_6 22
Figure S24: ^{1}H NMR of compound **4** in DMSO-d_6 (600 MHz)...............................22
Figure S25: ^{13}C NMR of compound **4** in DMSO-d_6 (150 MHz)..............................23
Figure S26: COSY spectrum of compound **4** in DMSO-d_6.......................................23
Figure S27: HSQC spectrum of compound **4** in DMSO-d_6 24
Figure S28: HMBC spectrum of compound **4** in DMSO-d_6 24
Figure S29: ^{1}H NMR of compound **5** in DMSO-d_6 (600 MHz)...............................25
Figure S30: ^{13}C NMR of compound **5** in DMSO-d_6 (150 MHz)..............................25

Figure S31: COSY spectrum of compound **5** in DMSO-d_6..26
Figure S32: HSQC spectrum of compound **5** in DMSO-d_6 26
Figure S33: HMBC spectrum of compound **5** in DMSO-d_6 27
Figure S34: ^{1}H NMR of compound **6** in DMSO-d_6 (600 MHz).................................28
Figure S35: ^{13}C NMR of compound **6** in DMSO-d_6 (150 MHz)................................28
Figure S36: COSY spectrum of compound **6** in DMSO-d_6..29
Figure S37: HSQC spectrum of compound **6** in DMSO-d_6 29
Figure S38: HMBC spectrum of compound **6** in DMSO-d_6 30
Figure S39: ^{1}H NMR of compound **7** in DMSO-d_6 (600 MHz).................................31
Figure S40: ^{13}C NMR of compound **7** in DMSO-d_6 (150 MHz)................................31
Figure S41: COSY spectrum of compound **7** in DMSO-d_6..32
Figure S42: HSQC spectrum of compound **7** in DMSO-d_6 32
Figure S43: HMBC spectrum of compound **7** in DMSO-d_6 33

Table S1: ^{1}H (600MHz) and ^{13}C (150 MHz) NMR data of **4M5C-CoA**

Position No.	δ_H (mult, J in Hz)[a]	δ_C (type)[b]	^{1}H-^{1}H COSY correlations	^{1}H-^{13}C HMBC correlations
1	6.16 (d, 5.6)	87.4 (CH)	2	2, 1′, 5′
2	4.85 (overlapping)	74.3 (CH)	1, 3	1
3	3.86 (m)	72.1 (CH)	2	4
4	4.60 (br s)	83.6 (CH)		1, 2, 5
5	4.02 (br d, 5.6)	65.2 (CH_2)		3, 4
	4.28 (br s)			
6	3.60 (m)	72.0 (CH_2)		7, 8, 15, 16
	3.86 (m)			
7		38.4 (C)		
8	4.03 (s)	74.1 (CH)		6, 7, 9, 15, 16
9		174.8 (C)		
10	3.47 (m)	35.4 (CH_2)	11	9, 11, 12
	3.47 (m)			
11	2.45 (t, 6.8)	35.5 (CH_2)	10	10, 12
	2.45 (t, 6.8)			
12		174.2 (C)		
13	3.44 (m)	38.6 (CH_2)	14	12, 14
	3.44 (m)			
14	3.20 (m)	29.4 (CH_2)	13	13, 1″
	3.20 (m)			
15	0.80 (s)	18.4 (CH_3)		6, 7, 8, 16
16	0.93 (s)	20.9 (CH_3)		6, 7, 8, 15
1′	8.64 (s)	142.2 (CH)		2′, 5′
2′		118.5 (C)		
3′		150.5 (C)		
4′	8.38 (s)	145.6 (CH)		3′, 5′
5′		148.6 (C)		
1″		185.8 (C)		
2″		130.3 (C)		
3″	8.98 (s)	157.7 (CH)		2″, 4″
4″		157.3 (C)		
5″	2.62 (s)	17.1(CH_3)		2″, 4″

[a] The δ_H values were measured in 600 MHz NMR.

[b] The δ_C values were measured in 150 MHz NMR.

Table S2: ^{1}H-NMR data of **1–7** in DMSO-d_6 [a]

No.	**1**	**2**	**3**	**4**	**5**	**6**	**7**
2	5.28 (s)	5.37 (s)	4.83 (s)	5.07 (s)	5.21 (s)	5.31 (s)	5.08 (s)
4	6.39 (s)	6.69 (s)	6.47 (s)	5.85 (s)	5.95 (s)	5.67 (s)	5.89 (s)
1′				2.41 (m)	2.40 (m)	1.98 (m)	3.78 (s)
				2.41 (m)	2.40 (m)	1.98 (m)	3.78 (s)
2′		8.03 (s)	7.96 (s)	1.24 (m)	1.23 (overlap)	1.23 (overlap)	
				1.24 (m)	1.23 (overlap)	1.23 (overlap)	
3′	9.15 (s)			1.55 (m)	1.29 (m)	1.27 (m)	7.34 (d, 7.8)
				1.55 (m)	1.29 (m)	1.27 (m)	
4′	2.59 (s)	2.71 (s)	--	2.62 (m)	1.57 (m)	1.23 (overlap)	7.59 (d, 7.8)
				2.62 (m)	1.57 (m)	1.23 (overlap)	
5′	--	--	--		2.56 (m)	1.54 (m)	
					2.56 (m)	1.54 (m)	
6′	--	--	--	7.08 (overlap)		2.53 (overlap)	7.59 (d, 7.8)
						2.53 (overlap)	
7′	--	--	--	7.22 (overlap)	7.17 (d, 7.5)		7.34 (d, 7.8)
8′	--	--	--		7.25 (d, 7.5)	7.16 (d, 7.5)	
9′	--	--	--	7.22 (overlap)	7.15 (t, 7.5)	7.24 (d, 7.5)	7.64 (d, 7.5)
10′	--	--	--	7.08 (overlap)	7.25 (d, 7.5)	7.15 (t, 7.5)	7.45 (dd, 7.5, 7.6)
11′	--	--	--	--	7.17 (d, 7.5)	7.24 (d, 7.5)	7.34 (t, 7.6)
12′	--	--	--	--	--	7.16 (d, 7.5)	7.45 (dd, 7.5, 7.6)
13′	--	--	--	--	--	--	7.64 (d, 7.5)

[a] The δ_H (mult, J in Hz) values were measured in 600 MHz NMR.

Table S3: ^{13}C-NMR data of **1–7**[a] in DMSO-d_6[a]

No.	**1**	**2**	**3**	**4**	**5**	**6**	**7**
1	173.6 (C)	173.3 (C)	164.3 (C)	undetected (C)	164.1 (C)	undetected (C)	163.6 (C)
2	89.3 (CH)	89.8 (CH)	88.5 (CH)	86.3 (CH)	88.3 (CH)	86.5 (CH)	87.8 (CH)
3	undetected [b] (C)	155.7 (C)	undetected (C)	undetected (C)	171.5 (C)	170.2 (C)	undetected (C)
4	100.8 (CH)	98.8 (CH)	107.1 (CH)	101.2 (CH)	99.9 (CH)	102.3 (CH)	101.9 (CH)
5	undetected (C)	undetected (C)	undetected (C)	165.1 (C)	166.3(C)	166.5 (C)	undetected (C)
1′	152.8 (C)	145.6 (C)	151.8 (C)	32.6 (CH_2)	32.8 (CH_2)	34.4 (CH_2)	38.5 (CH_2)
2′	123.7 (C)	120.4 (CH)	121.1 (CH)	20.7 (CH_2)	27.2 (CH_2)	20.6 (CH_2)	134.5 (C)
3′	154.8 (CH)	167.3 (C)	146.4 (C)	30.4 (CH_2)	27.9 (CH_2)	28.4 (CH_2)	129.7 (CH)
4′	17.4 (CH_3)	18.9 (CH_3)	--	33.8 (CH_2)	30.6 (CH_2)	29.0 (CH_2)	126.5 (CH)
5′	--	--	--	139.1 (C)	35.0 (CH_2)	30.9 (CH_2)	138.5 (C)
6′	--	--	--	130.0 (CH)	142.2 (C)	35.1 (CH_2)	126.5 (CH)
7′	--	--	--	114.9 (CH)	128.3 (CH)	142.4 (C)	129.7 (CH)
8′	--	--	--	159.7 (C)	128.3 (CH)	128.2 (CH)	140.0 (C)
9′	--	--	--	114.9 (CH)	125.7 (CH)	128.2 (CH)	126.6 (CH)
10′	--	--	--	130.0(CH)	128.3 (CH)	125.6 (CH)	128.8 (CH)
11′	--	--	--	--	128.3 (CH)	128.2 (CH)	127.3 (CH)
12′	--	--	--	--	--	128.2 (CH)	128.8 (CH)
13′	--	--	--	--	--	--	126.6 (CH)

[a] The δ_C (type) values were measured in 150 MHz NMR.

[b] The chemical shifts cannot be observed were noted "undetected"

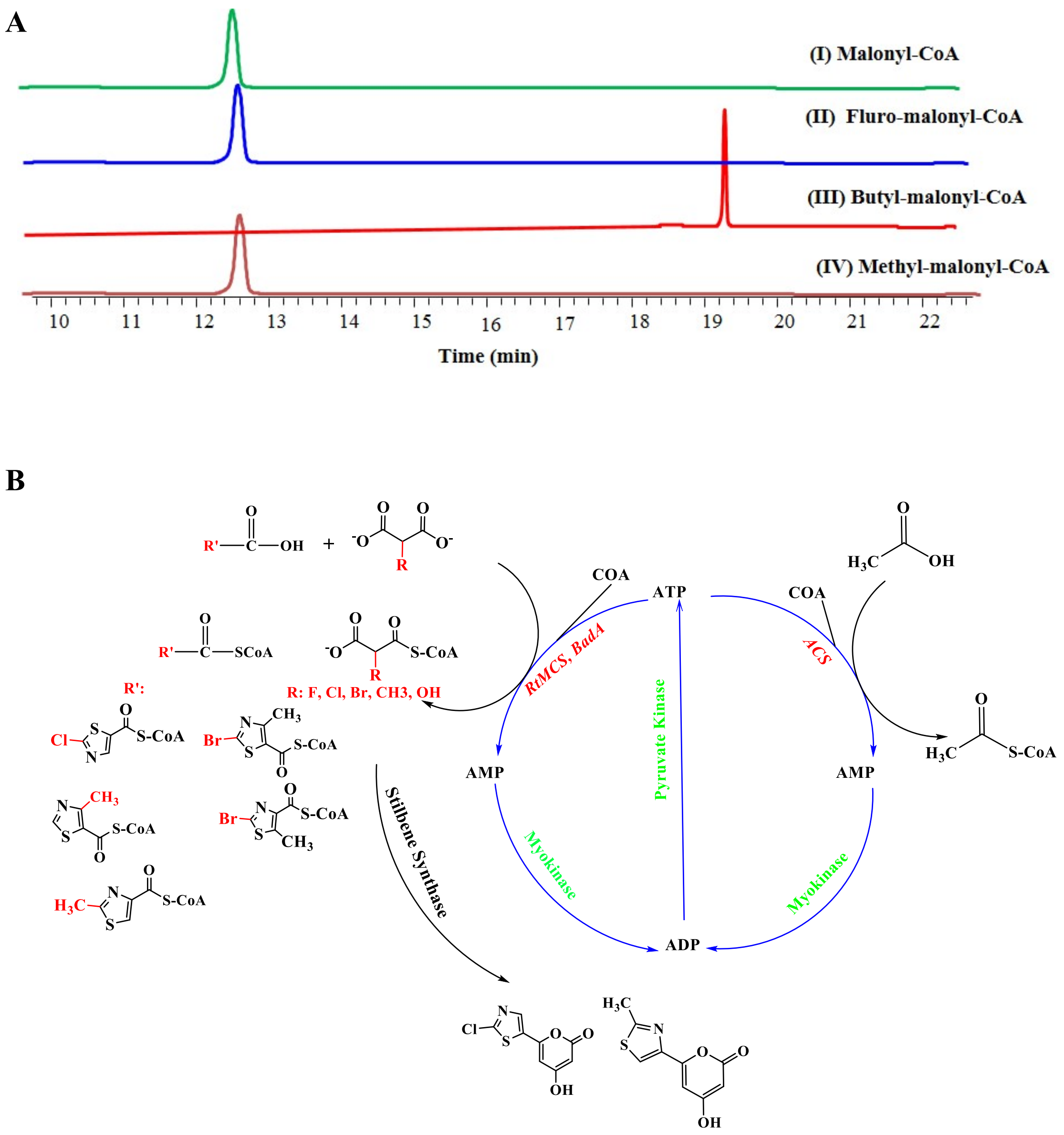

Figure S1: LC trace of malonyl-CoA derivatives and cyclic ATP regenerating system. (A) Chemo-enzymatic synthesis of malonyl-CoA and its derivatives (I) malonyl-CoA (II) fluoro-malonyl-CoA (III) butyl-malonyl-CoA (IV) methyl-malonyl-CoA CoA by RtMCS (B) Cyclic ATP regenerating myokinase/pyruvate kinase-PEP system.

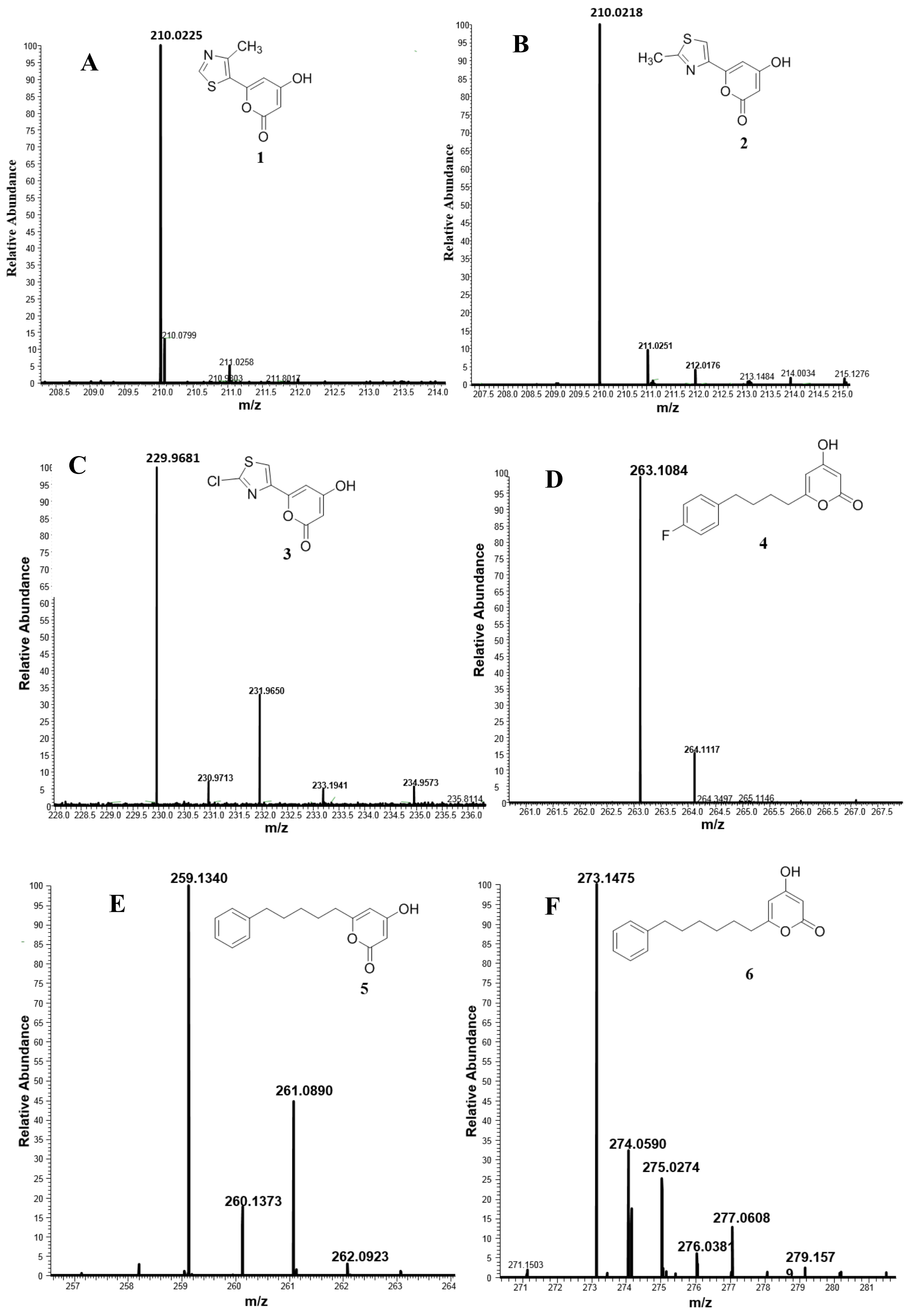
A
210.0225
210.0799
211.0258
210.9303
211.8017
1
B
210.0218
211.0251
212.0176
213.1484
214.0034
215.1276
2
C
229.9681
231.9650
230.9713
233.1941
234.9573
235.8114
3
D
263.1084
264.1117
264.3497
265.1146
4
E
259.1340
261.0890
260.1373
262.0923
5
F
273.1475
274.0590
275.0274
277.0608
276.038
279.157
271.1503
6
Relative Abundance
m/z

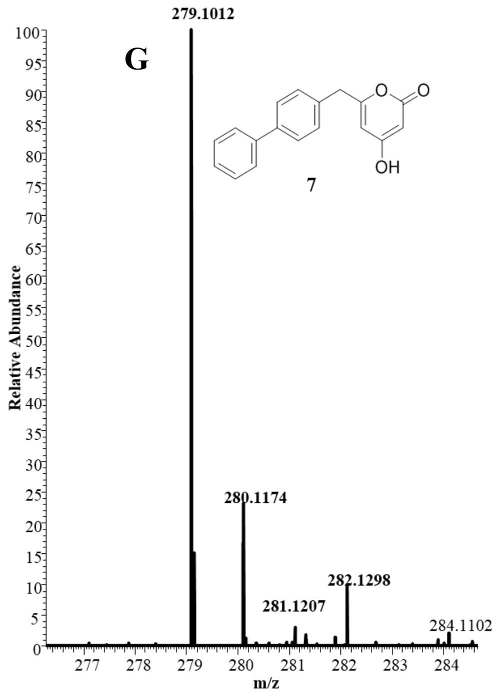

Figure S2: HPLC-PDA-HRQTOF-ESI/MS spectra of the newly synthesized polyketides products.

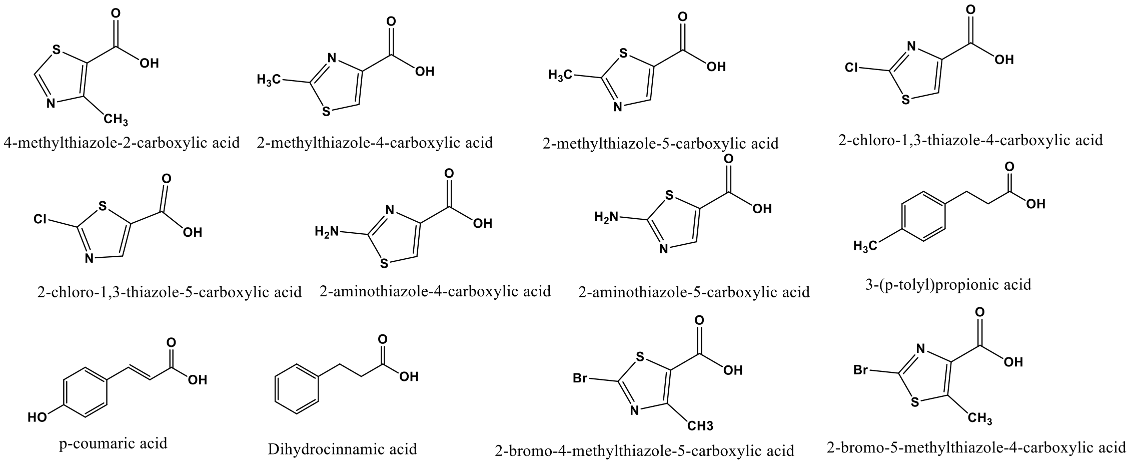

Figure S3: Chemical structure of carboxylic acids used as a substrate for BadA-mutant (H333A/I334A) to synthesized the CoA derivatives.

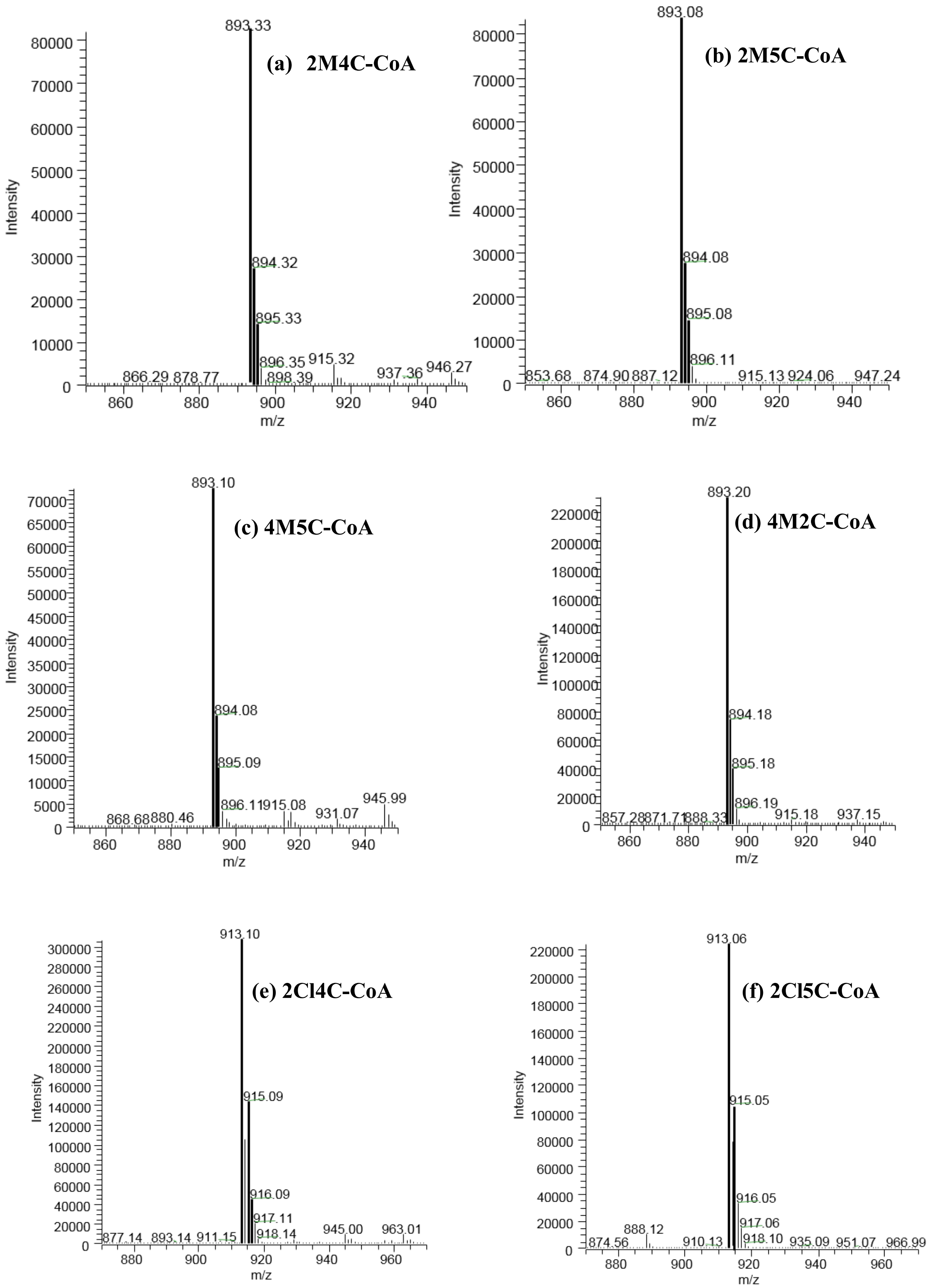
(a) 2M4C-CoA
893.33
894.32
895.33
896.35
898.39
915.32
866.29
878.77
937.36
946.27
(b) 2M5C-CoA
893.08
894.08
895.08
896.11
853.68
874.90
887.12
915.13
924.06
947.24
(c) 4M5C-CoA
893.10
894.08
895.09
896.11
915.08
868.68
880.46
931.07
945.99
(d) 4M2C-CoA
893.20
894.18
895.18
896.19
857.28
871.71
888.33
915.18
937.15
(e) 2Cl4C-CoA
913.10
915.09
916.09
917.11
918.14
877.14
893.14
911.15
945.00
963.01
(f) 2Cl5C-CoA
913.06
915.05
916.05
917.06
918.10
874.56
888.12
910.13
935.09
951.07
966.99
Intensity
m/z

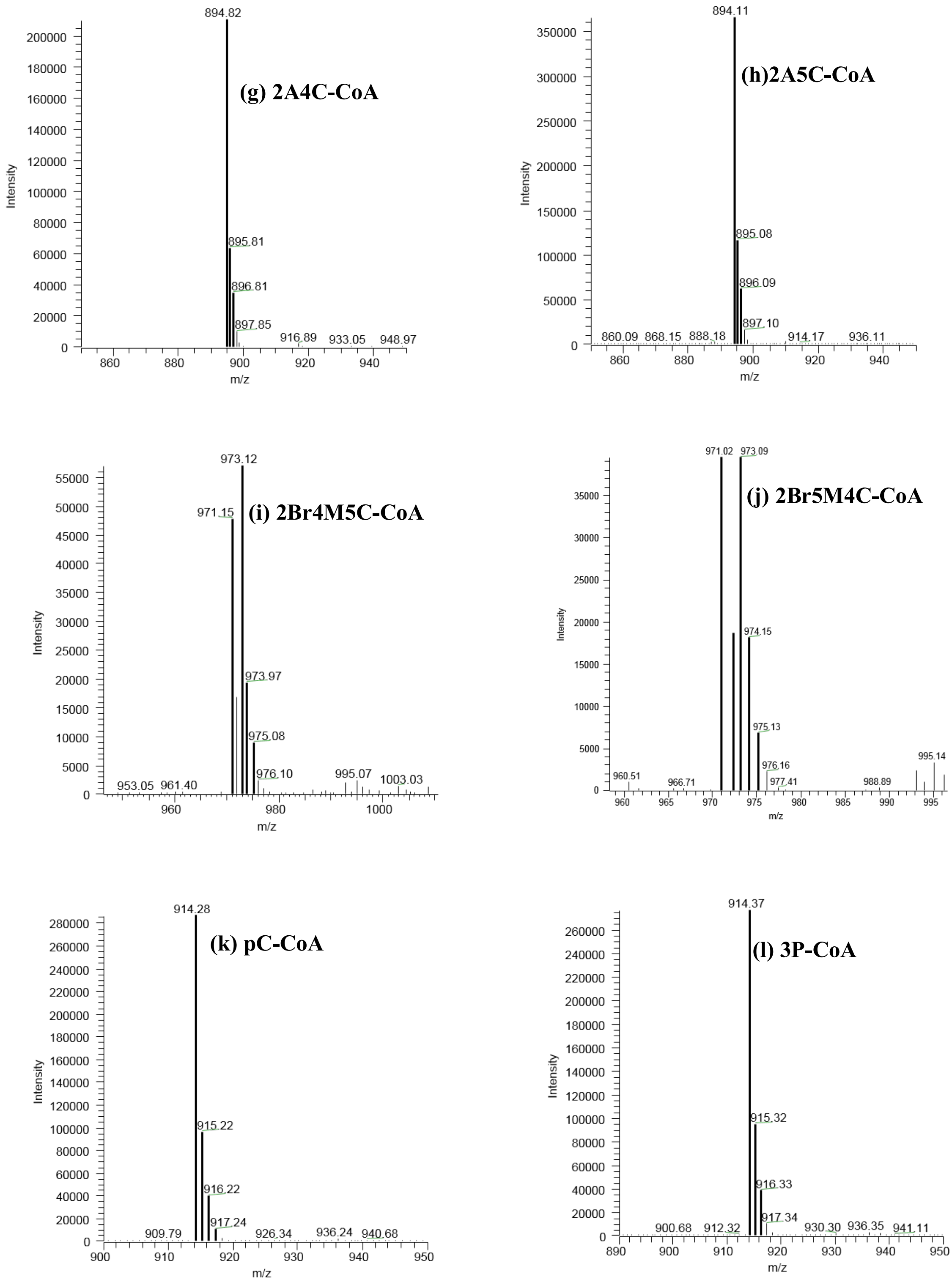
(g) 2A4C-CoA
894.82
895.81
896.81
897.85
916.89
933.05
948.97
Intensity
m/z
(h)2A5C-CoA
894.11
895.08
896.09
897.10
860.09
868.15
888.18
914.17
936.11
Intensity
m/z
(i) 2Br4M5C-CoA
973.12
971.15
973.97
975.08
976.10
953.05
961.40
995.07
1003.03
Intensity
m/z
(j) 2Br5M4C-CoA
971.02
973.09
974.15
975.13
976.16
977.41
960.51
966.71
988.89
995.14
Intensity
m/z
(k) pC-CoA
914.28
915.22
916.22
917.24
909.79
926.34
936.24
940.68
Intensity
m/z
(l) 3P-CoA
914.37
915.32
916.33
917.34
900.68
912.32
930.30
936.35
941.11
Intensity
m/z

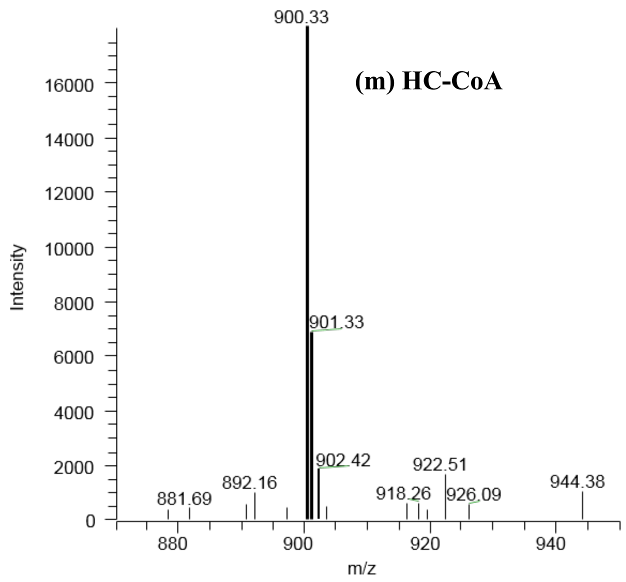

Figure S4: Mass spectrum [M+H]+ of representative CoA derivatives. (a) 2-methylthiazole-4-carboxyl-CoA (2M4C-CoA) (b) 2-methylthiazole-5-carboxyl-CoA (2M5C-CoA) (c) 4-methylthiazole-5-carboxyl-CoA (4M5C-CoA) (d) 4-methylthiazole-2-carboxyl-CoA (4M2C-CoA) (e) 2-chloro-1,3-thiazole-4-carboxyl-CoA (2Cl4C-CoA) (f) 2-chloro-1,3-thiazole-5-carboxyl-CoA 2Cl5C-CoA (g) 2-aminothiazole-4-carboxyl-CoA (2A4C-CoA) (h) 2-aminothiazole-5-carboxyl-CoA (2A5C-CoA) (i) 2-bromo-4-methylthiazole-5-carboxyl-CoA (2Br4M5C-CoA) (j) 2-bromo-5-methylthiazole-4-carboxyl-CoA (2Br5M4C-CoA) (k) p-coumaroyl-CoA (pC-CoA) (l) 3-(p-tolyl)propanol-CoA (3P-CoA) (m) dihydrocinnamoyl-CoA (HC-CoA)

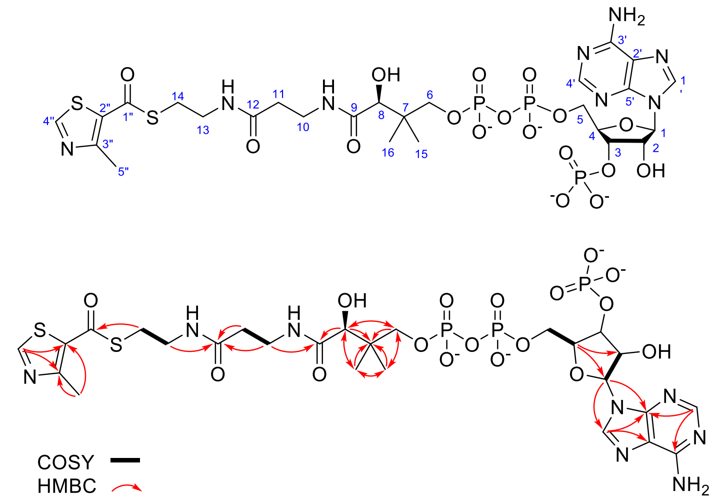

Figure S5: The COSY and HMBC correlation of **4M5C-CoA**

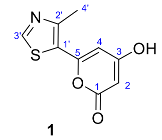

1

Chemical Formula: $C_9H_7NO_3S$
Exact Mass: 209.0147
Molecular Weight: 209.2190

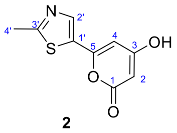

2

Chemical Formula: $C_9H_7NO_3S$
Exact Mass: 209.0147
Molecular Weight: 209.2190

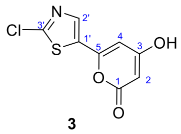

3

Chemical Formula: $C_8H_4ClNO_3S$
Exact Mass: 228.9600
Molecular Weight: 229.6340

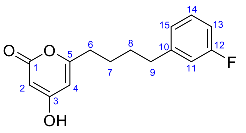

4

Chemical Formula: $C_{15}H_{15}FO_3$
Exact Mass: 262.1005
Molecular Weight: 262.2804

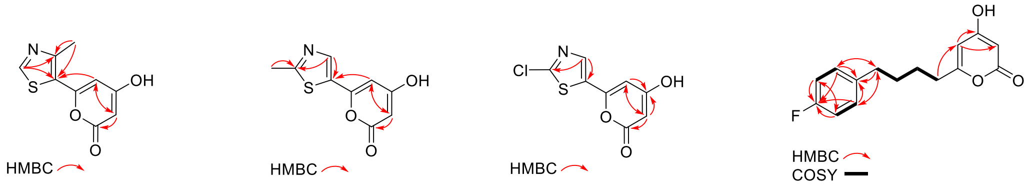

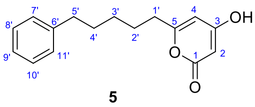

5

Chemical Formula: $C_{16}H_{18}O_3$
Exact Mass: 258.1256
Molecular Weight: 258.3170

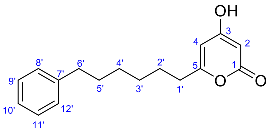

6

Chemical Formula: $C_{17}H_{20}O_3$
Exact Mass: 272.1412
Molecular Weight: 272.3440

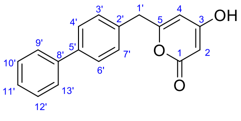

7

Chemical Formula: $C_{18}H_{14}O_3$
Exact Mass: 278.0943
Molecular Weight: 278.3070

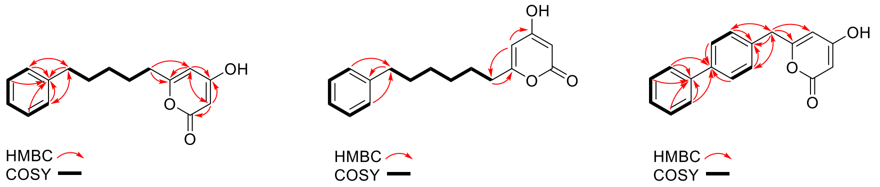

Figure S6: The assignment, physic data, 2D-NMR (HMBC and COSY) correlations of **1–7**. The COSY correlations were presented in bold lines while the HMBC correlations were shown red arrows.

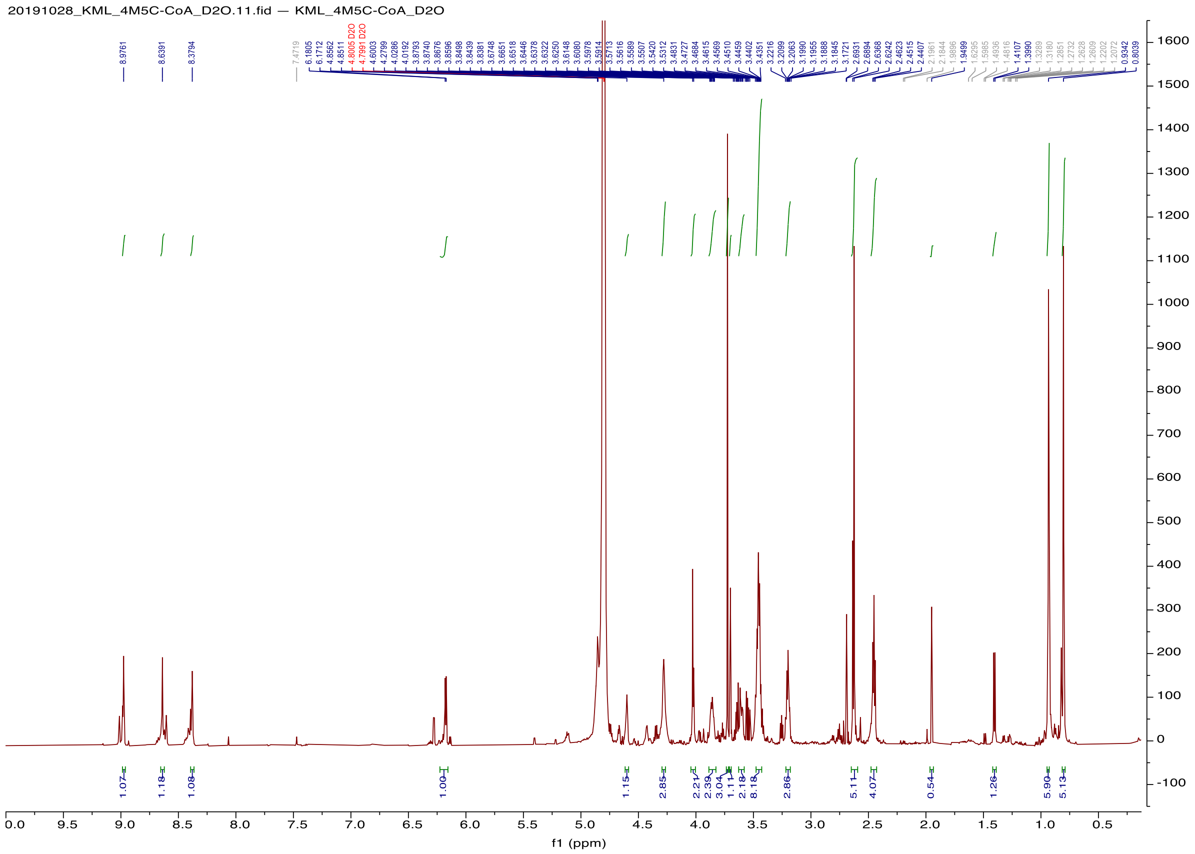

Figure S7. ^{1}H NMR of **4M5C-CoA** in D_2O (600 MHz)

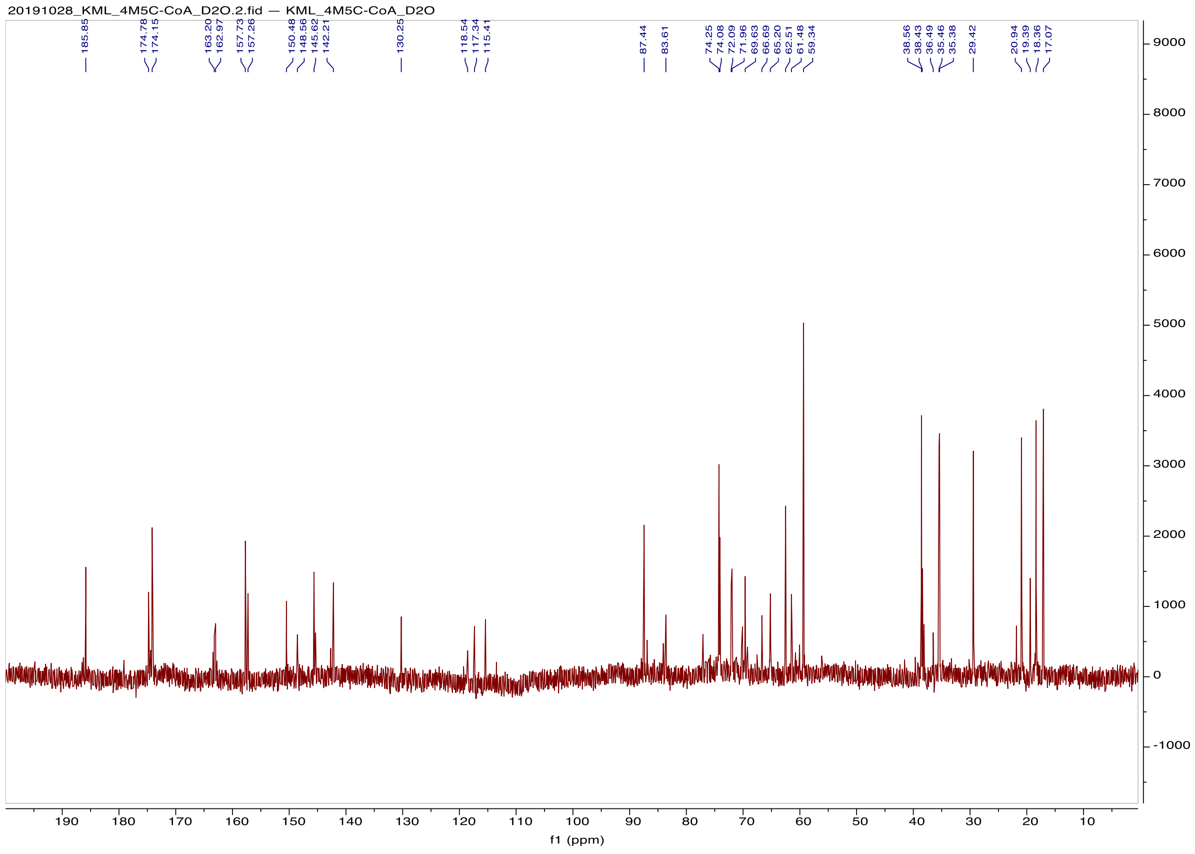

Figure S8: ^{13}C NMR of **4M5C-CoA** in D_2O (150 MHz)

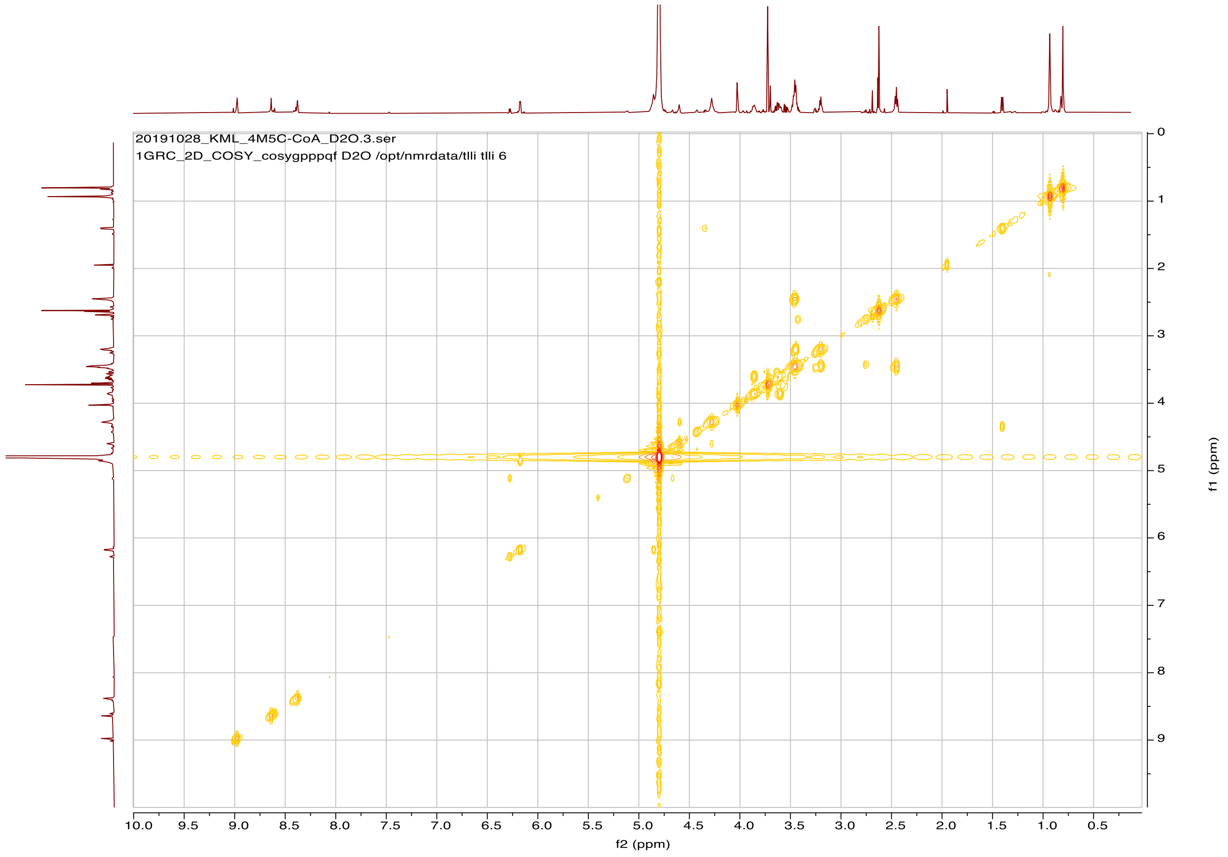

Figure S9: COSY spectrum of **4M5C-CoA** in D_2O

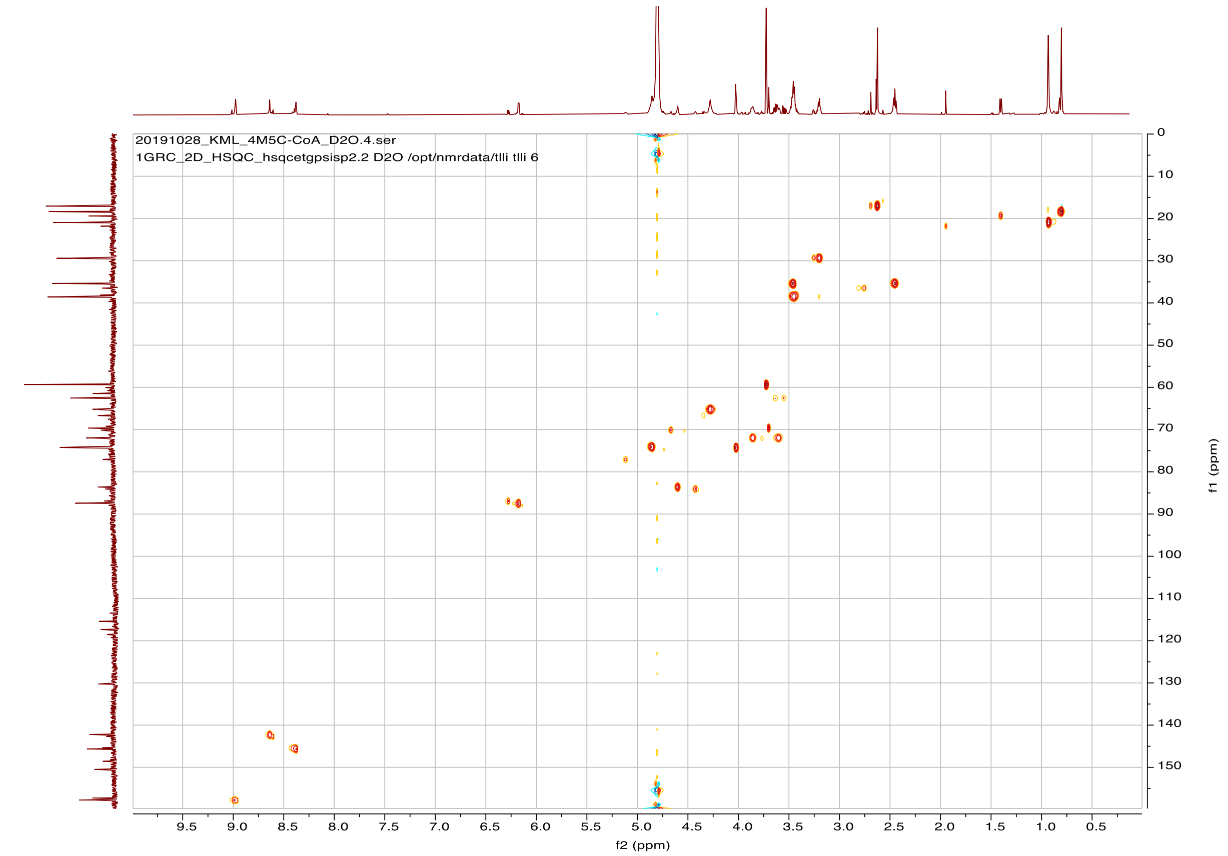

Figure S10: HSQC spectrum of **4M5C-CoA** in D_2O

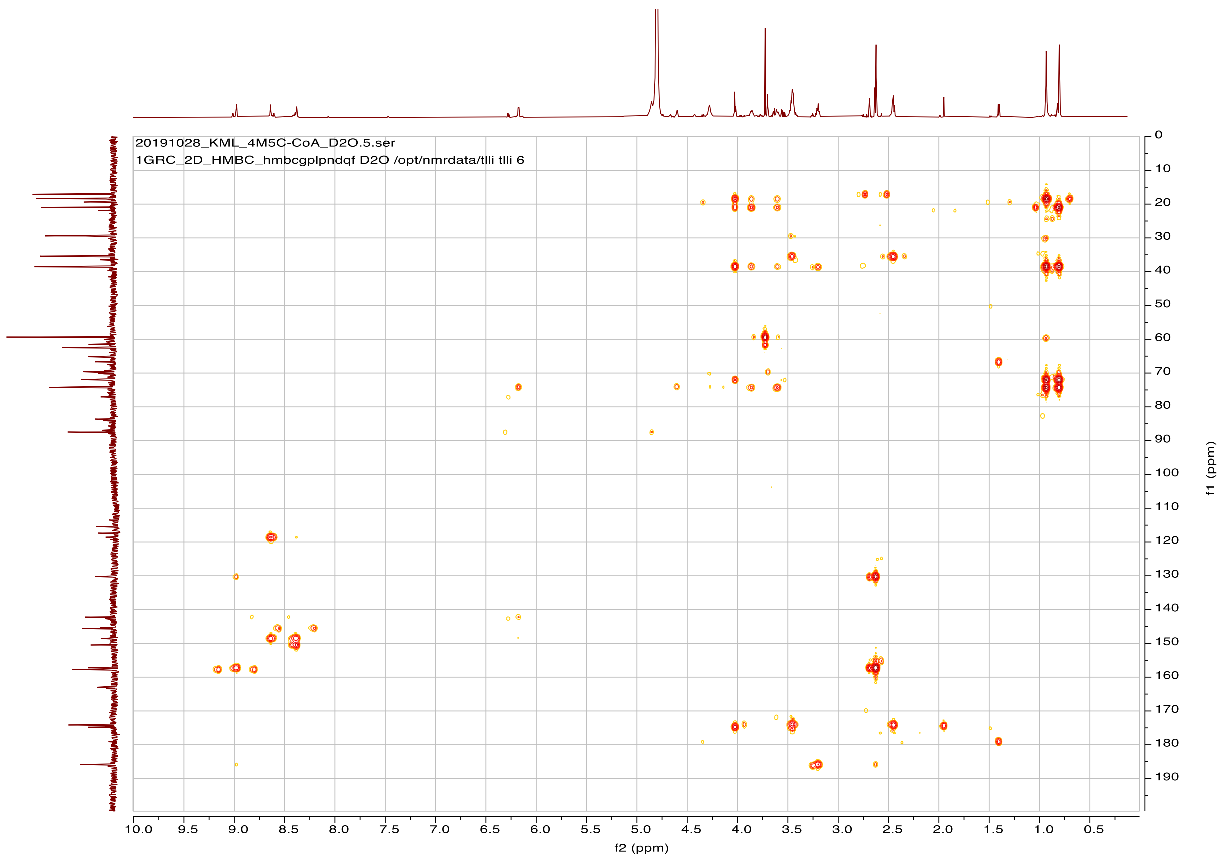

Figure S11. HMBC spectrum of **4M5C-CoA** in D_2O

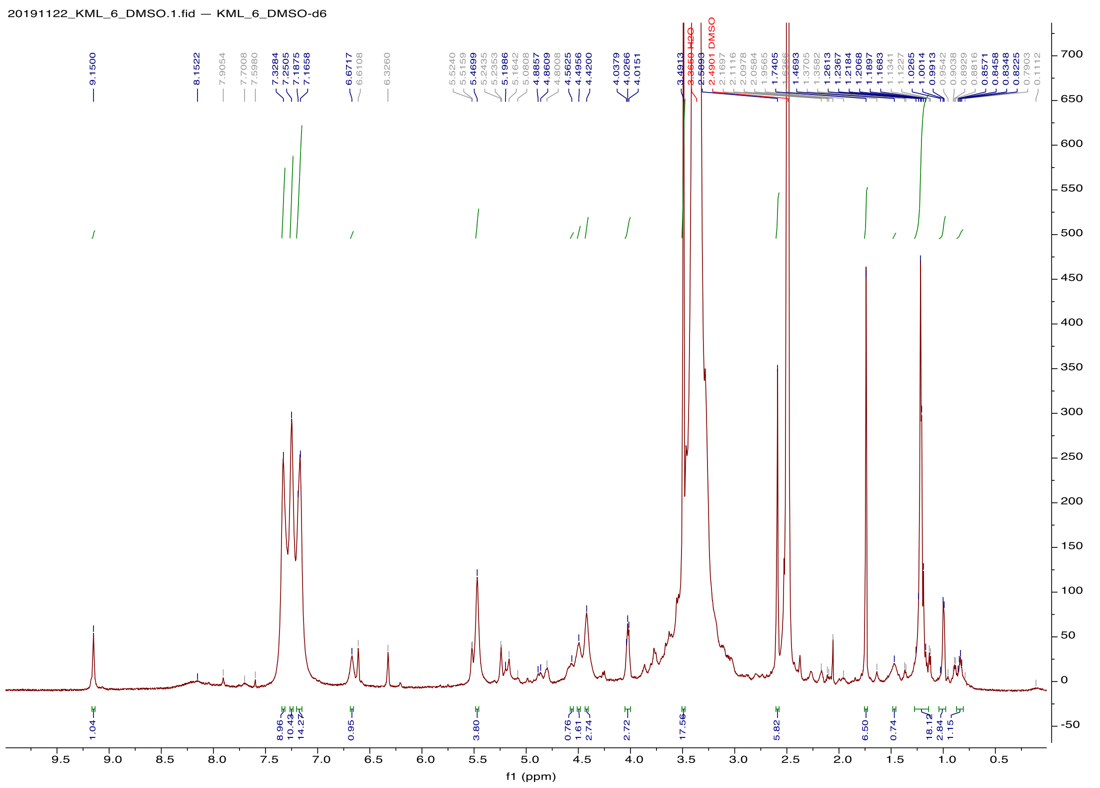

Figure S12: ^{1}H NMR of compound **1** in DMSO-d_6 (600 MHz)

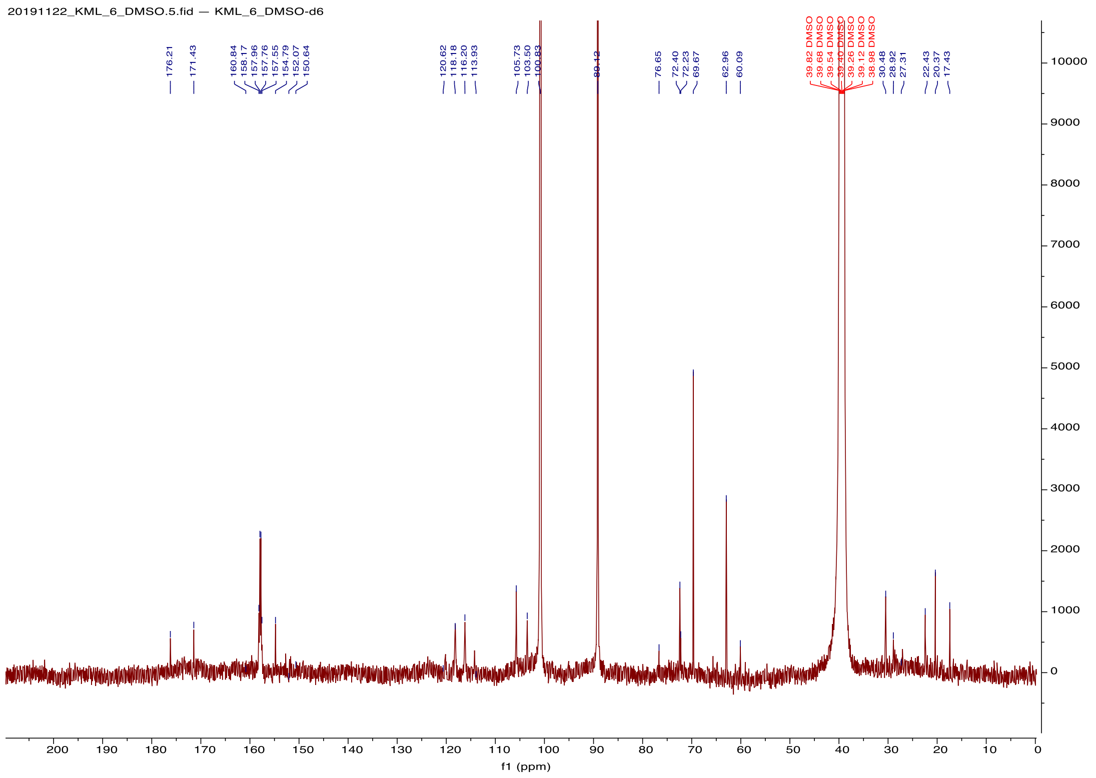

Figure S13: ^{13}C NMR of compound **1** in DMSO-d_6 (150 MHz)

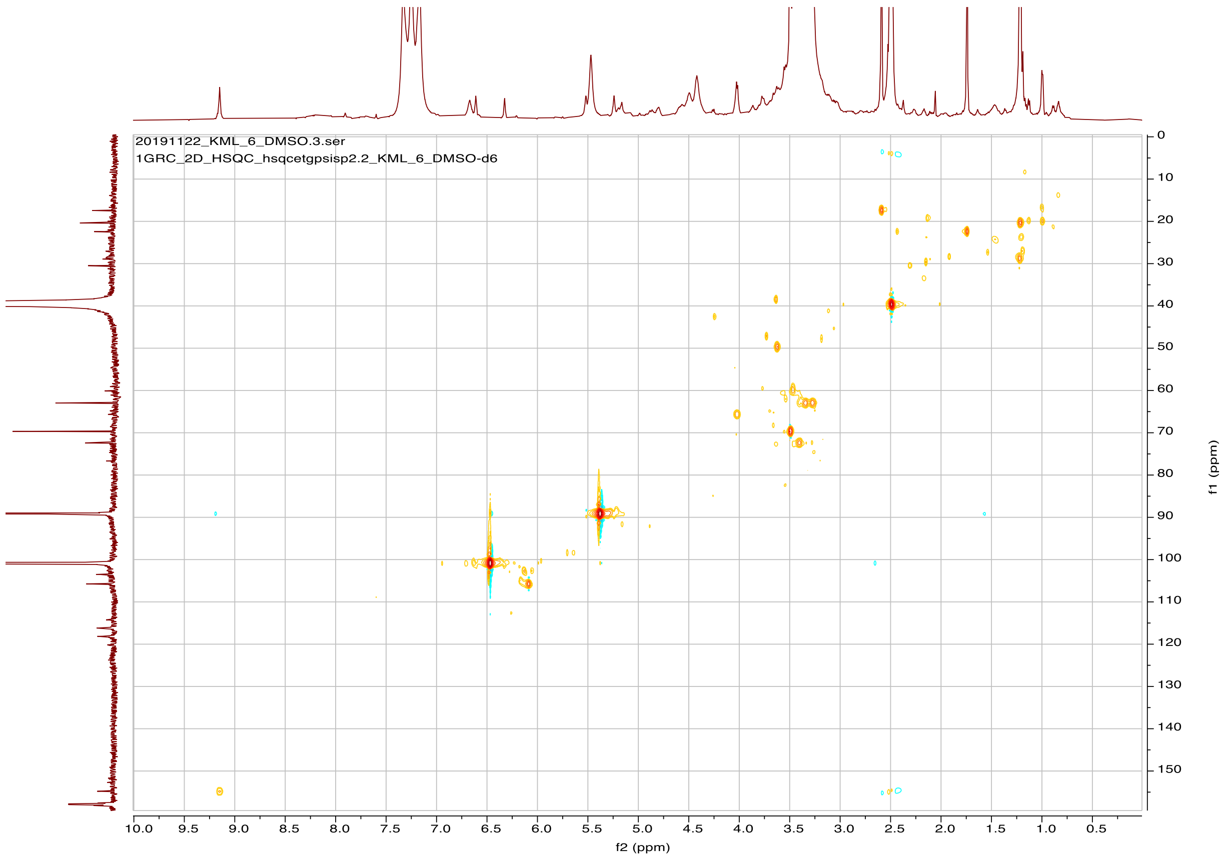

Figure S14: HSQC spectrum of compound **1** in DMSO-d_6

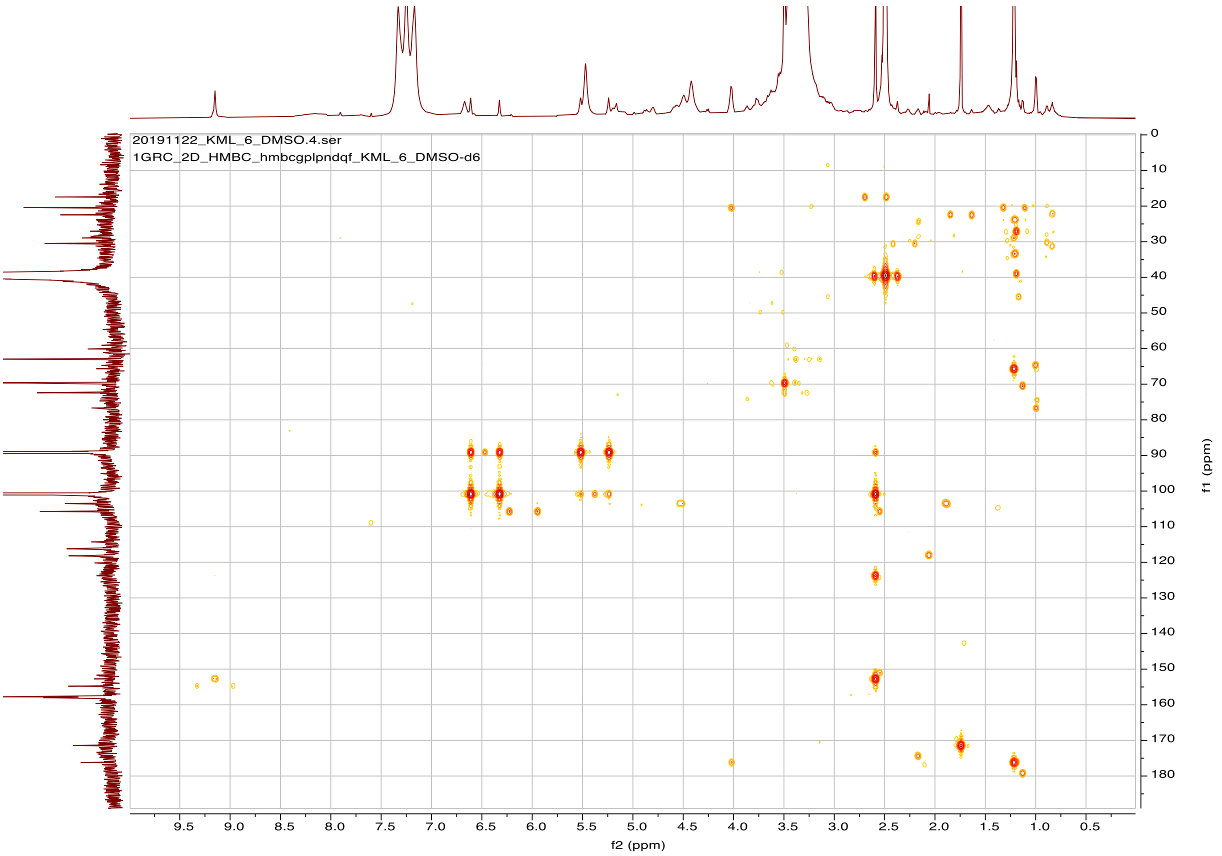

Figure S15: HMBC spectrum of compound **1** in DMSO-d_6

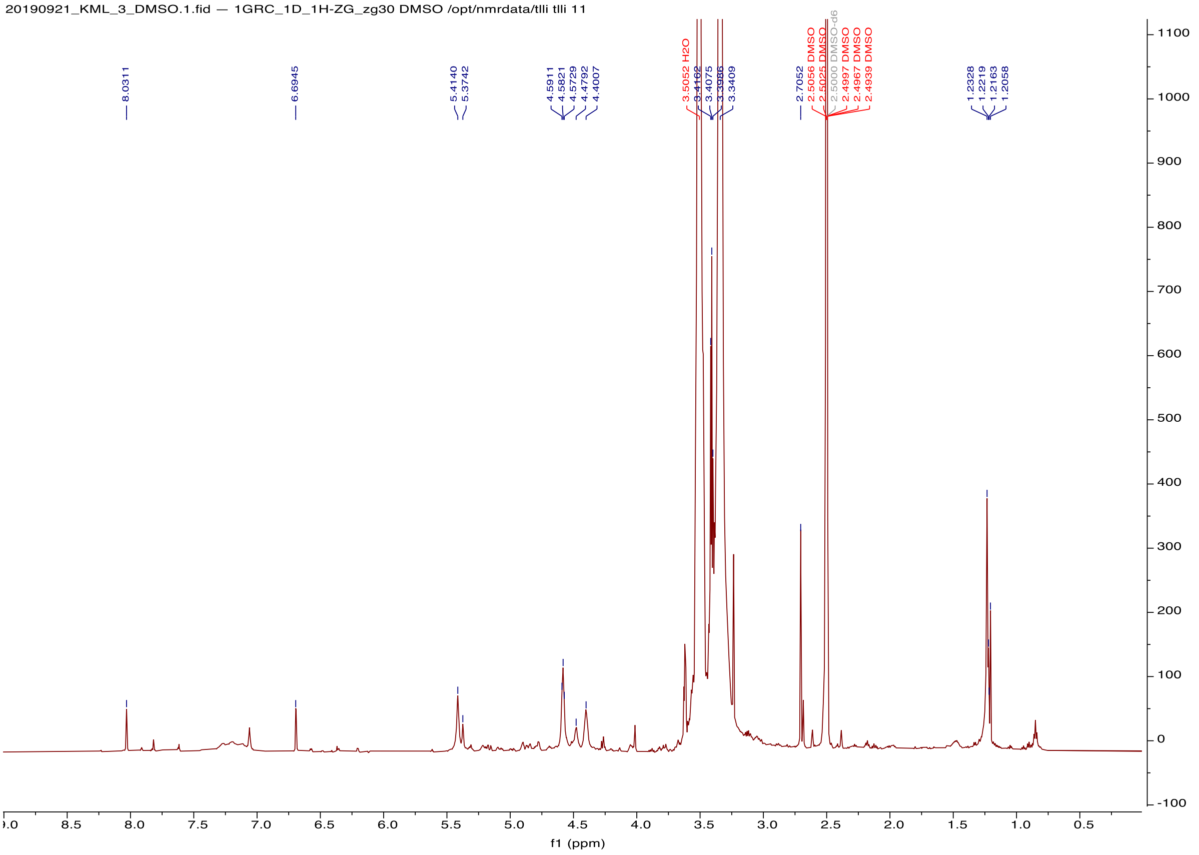

Figure S16: ^{1}H NMR of compound **2** in DMSO-d_6 (600 MHz)

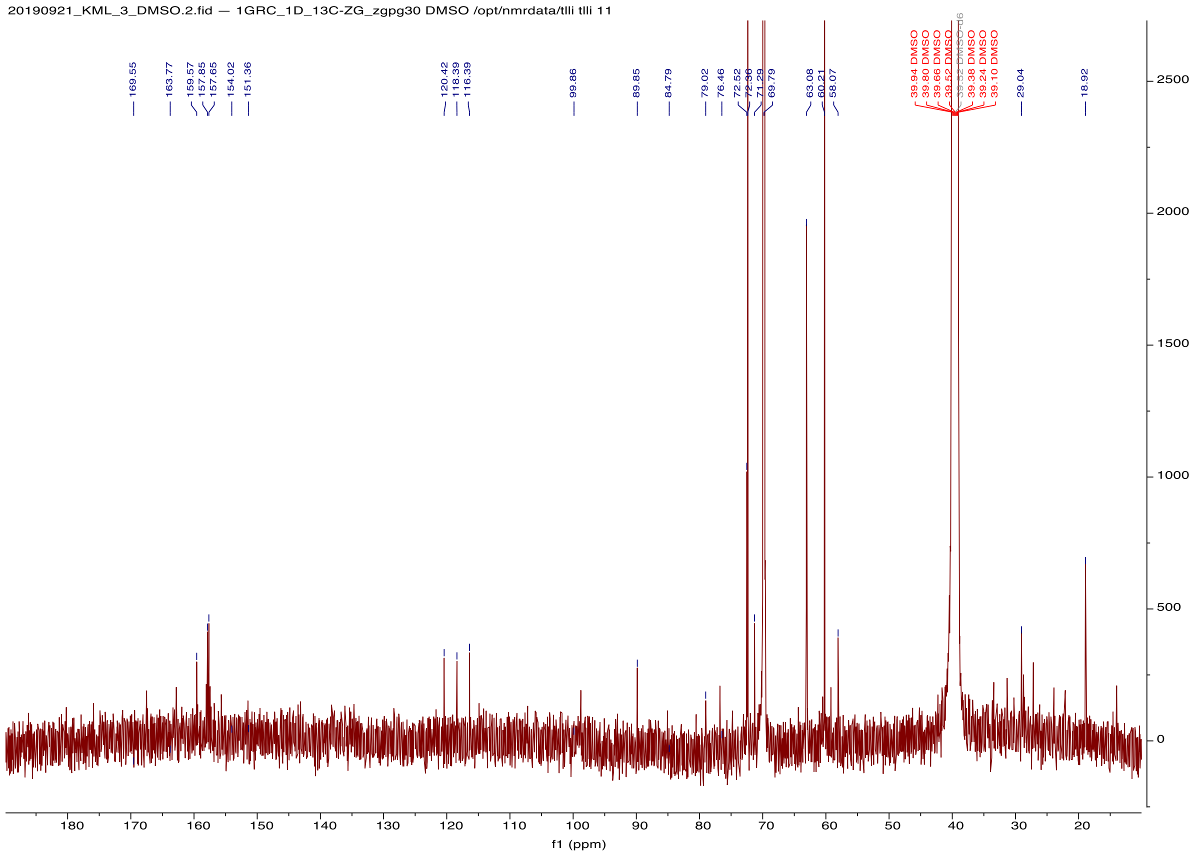

Figure S17: ^{13}C NMR of compound **2** in DMSO-d_6 (150 MHz)

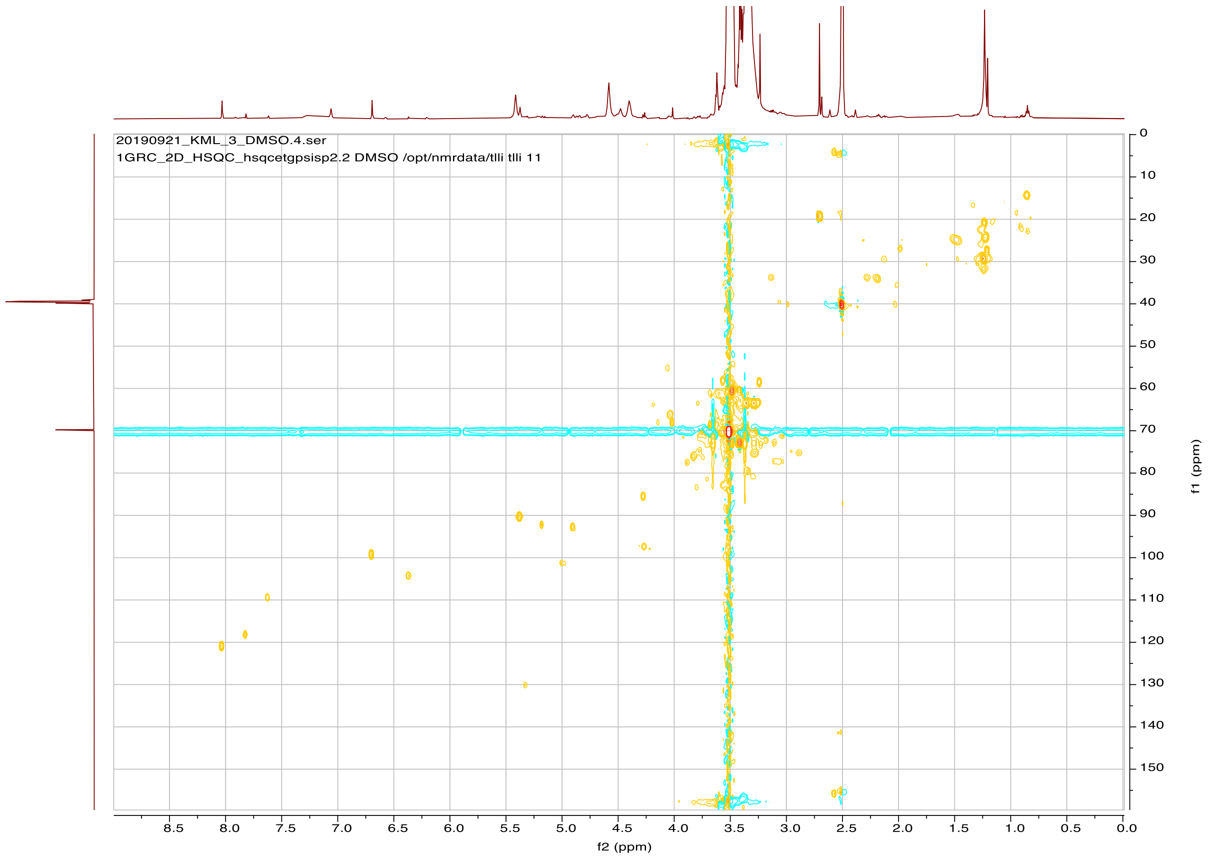

Figure S18: HSQC spectrum of compound **2** in DMSO-d_6

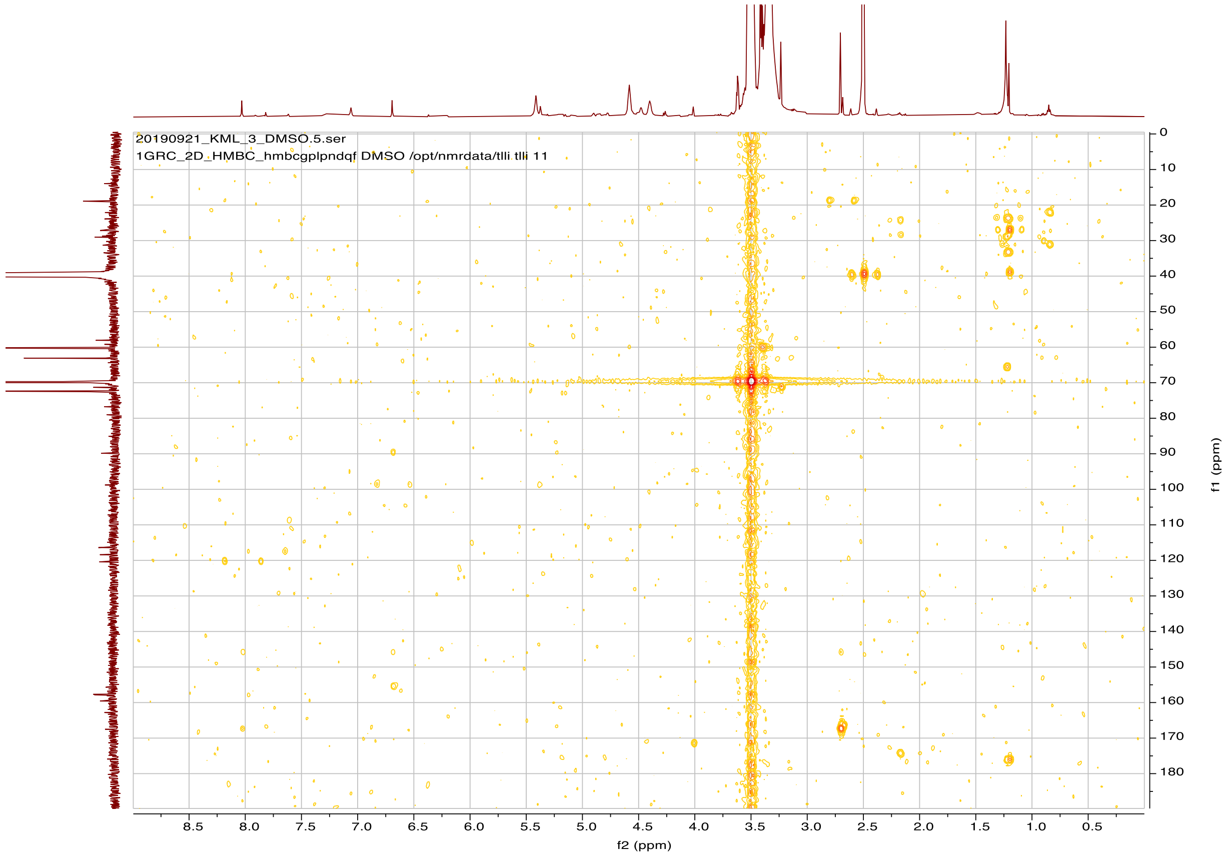

Figure S19: HMBC spectrum of compound **2** in DMSO-d_6

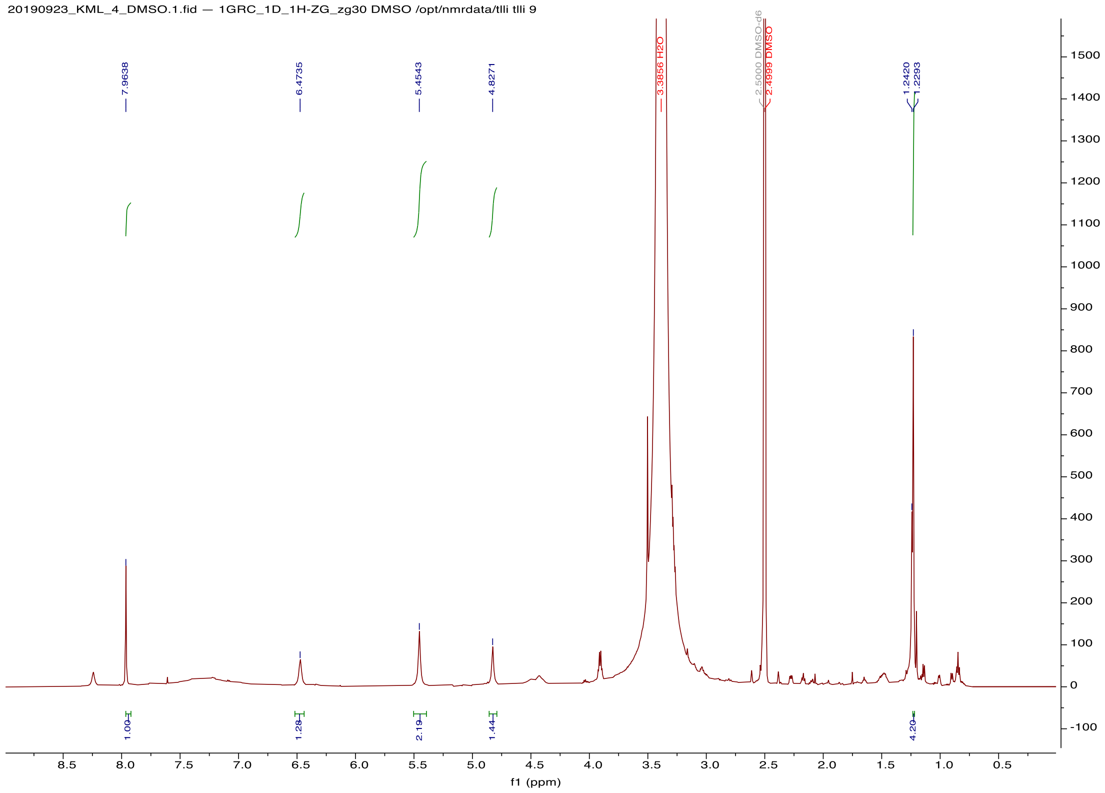

Figure S20: ^{1}H NMR of compound **3** in DMSO-d_6 (600 MHz)

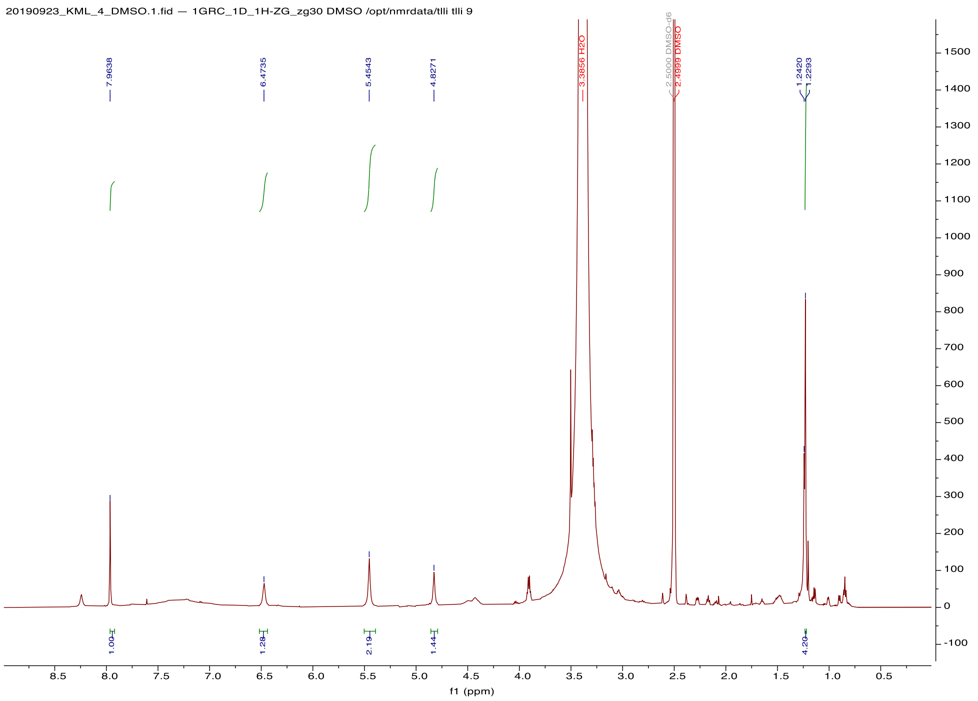

Figure S21:^{13}C NMR of compound **3** in DMSO-d_6 (150 MHz)

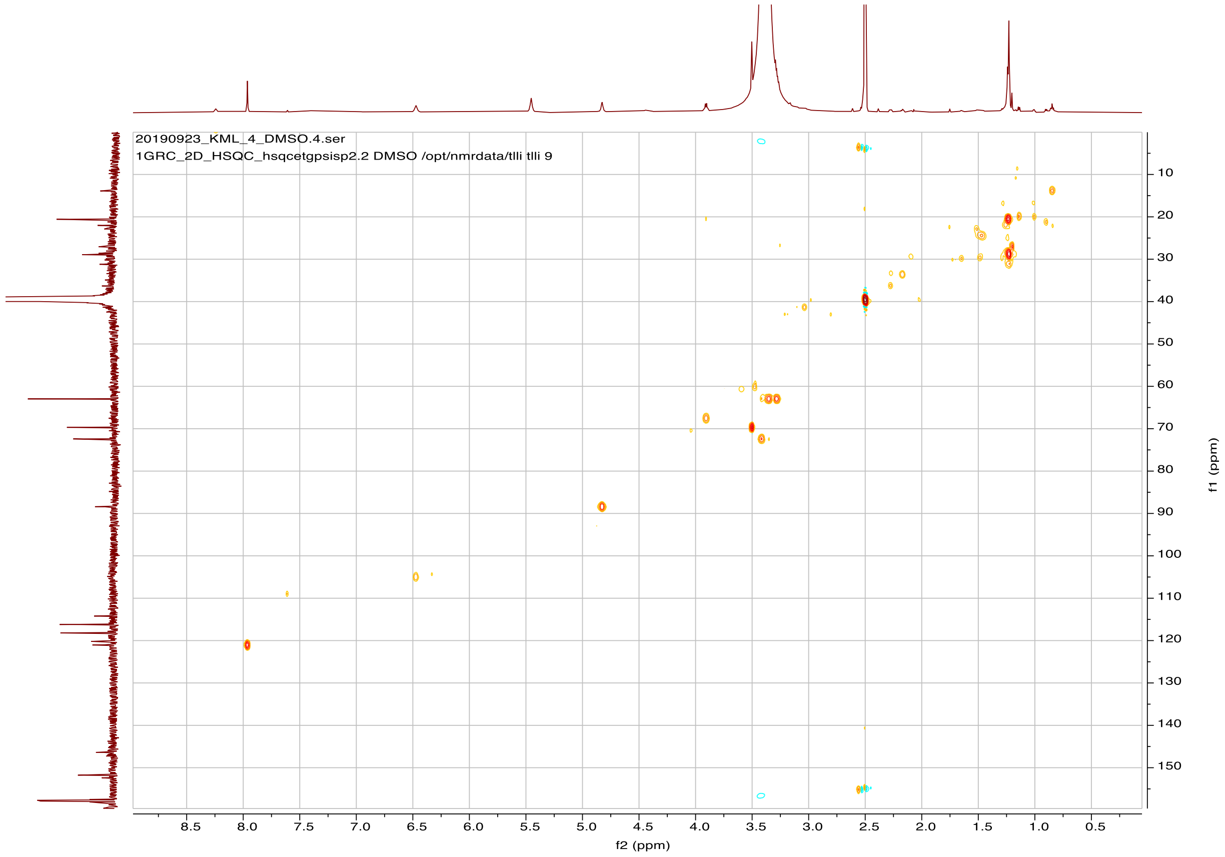

Figure S22: HSQC spectrum of compound **3** in DMSO-d_6

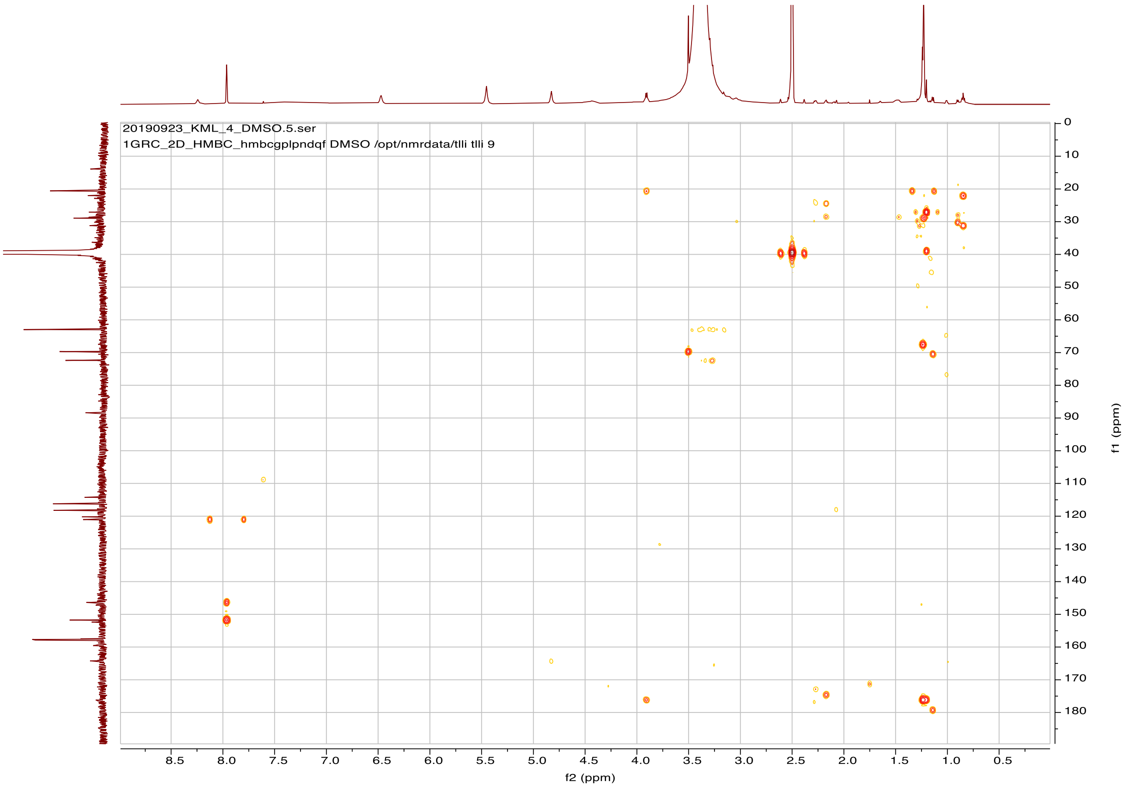

Figure S23: HMBC spectrum of compound **3** in DMSO-d_6

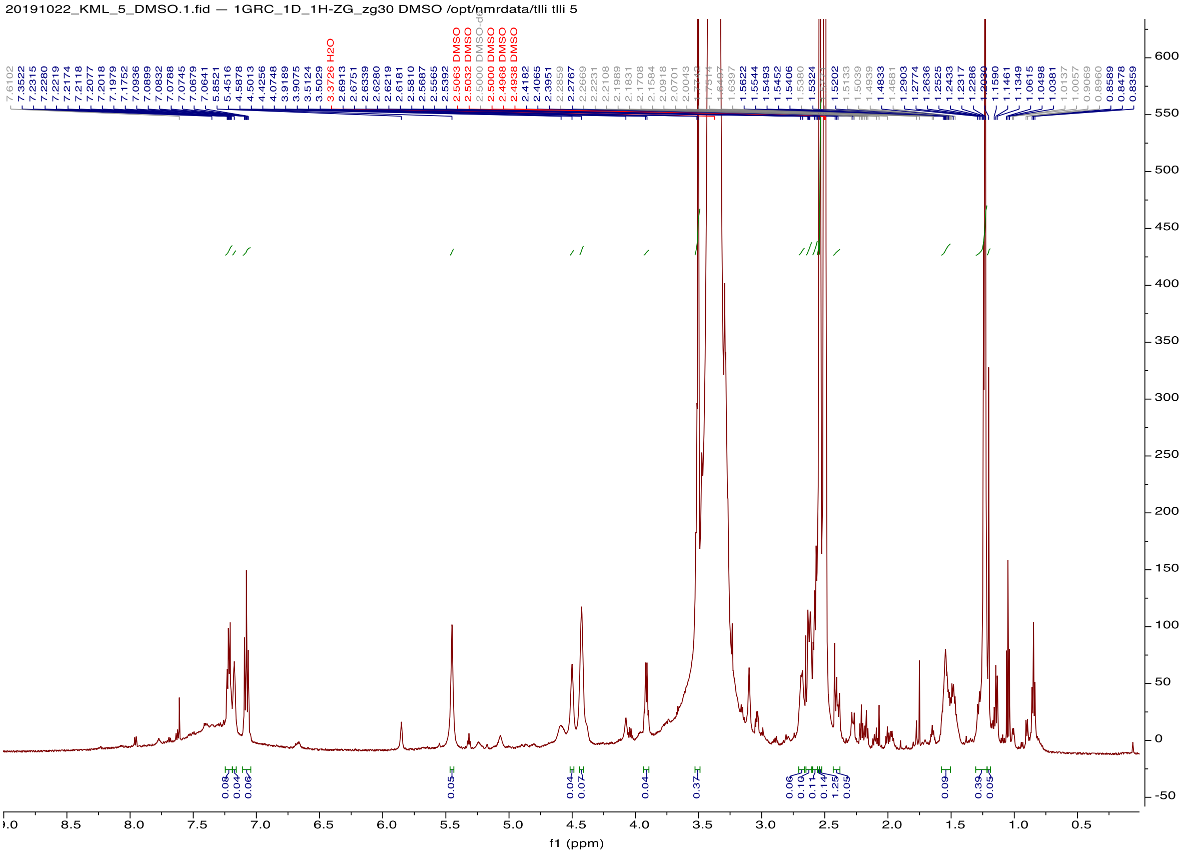

Figure S24: ^{1}H NMR of compound **4** in DMSO-d_6 (600 MHz)

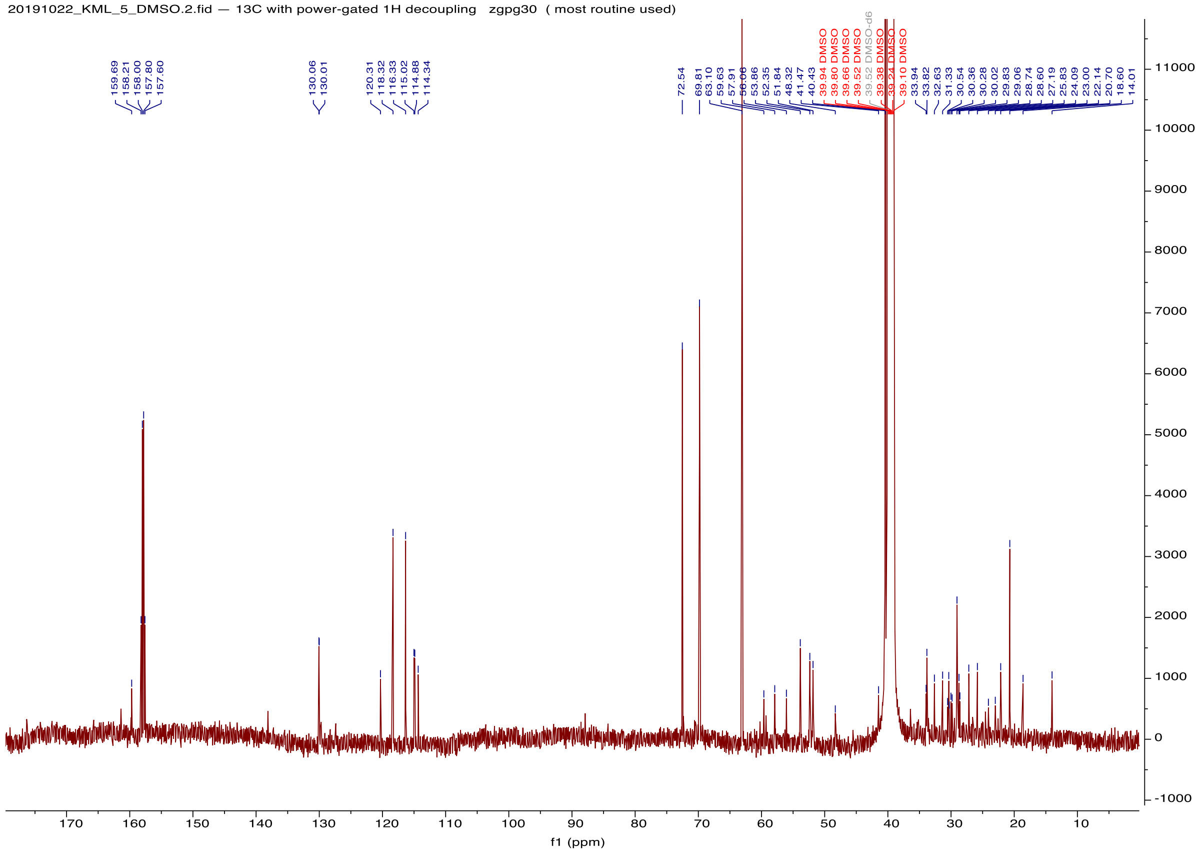

Figure S25: ^{13}C NMR of compound **4** in DMSO-d_6 (150 MHz)

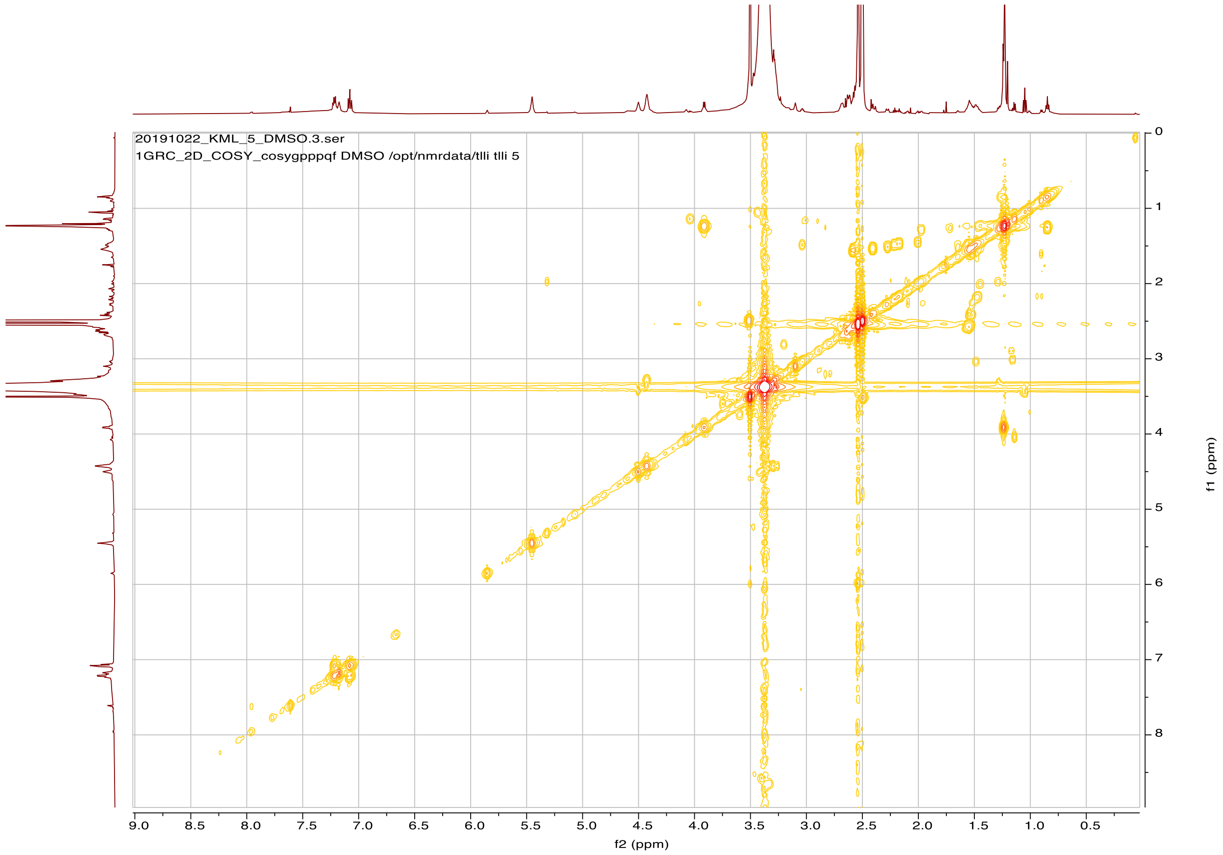

Figure S26: COSY spectrum of compound **4** in DMSO-d_6

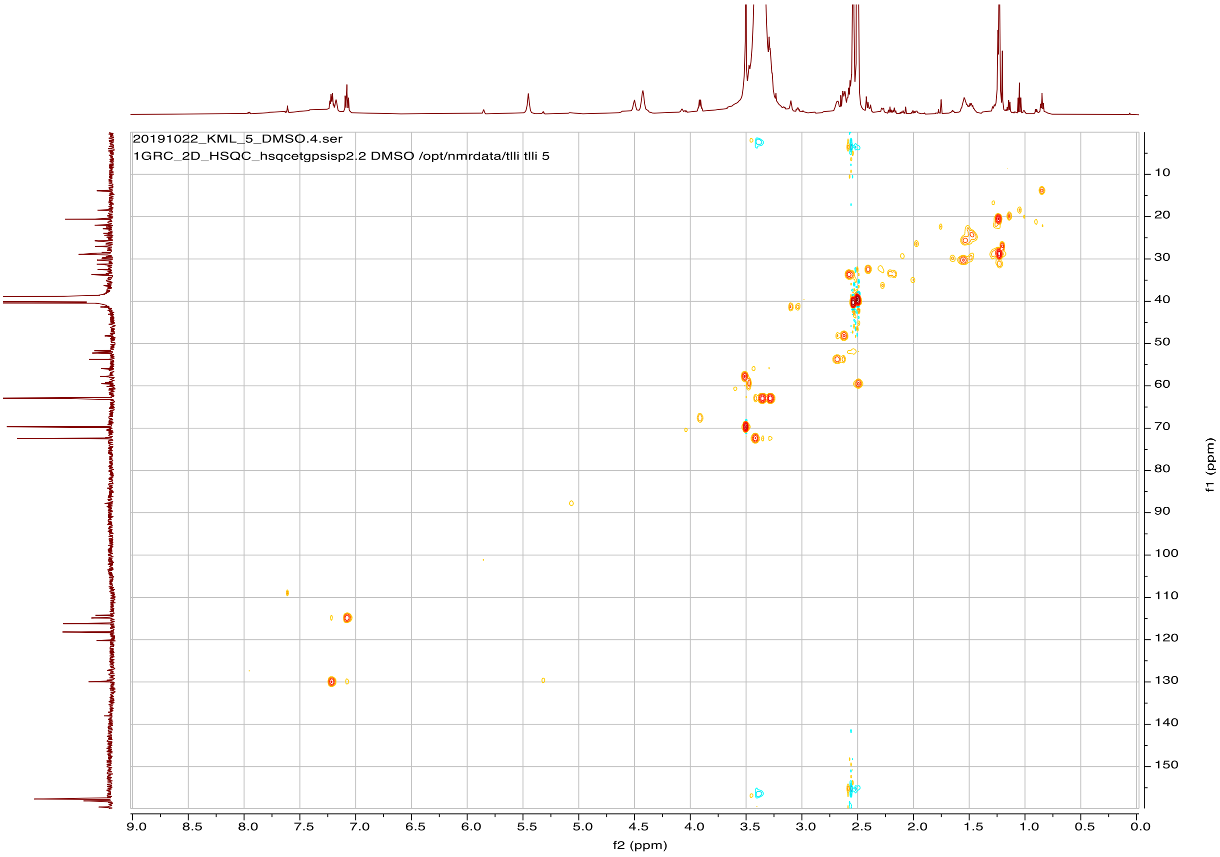

Figure S27: HSQC spectrum of compound **4** in DMSO-d_6

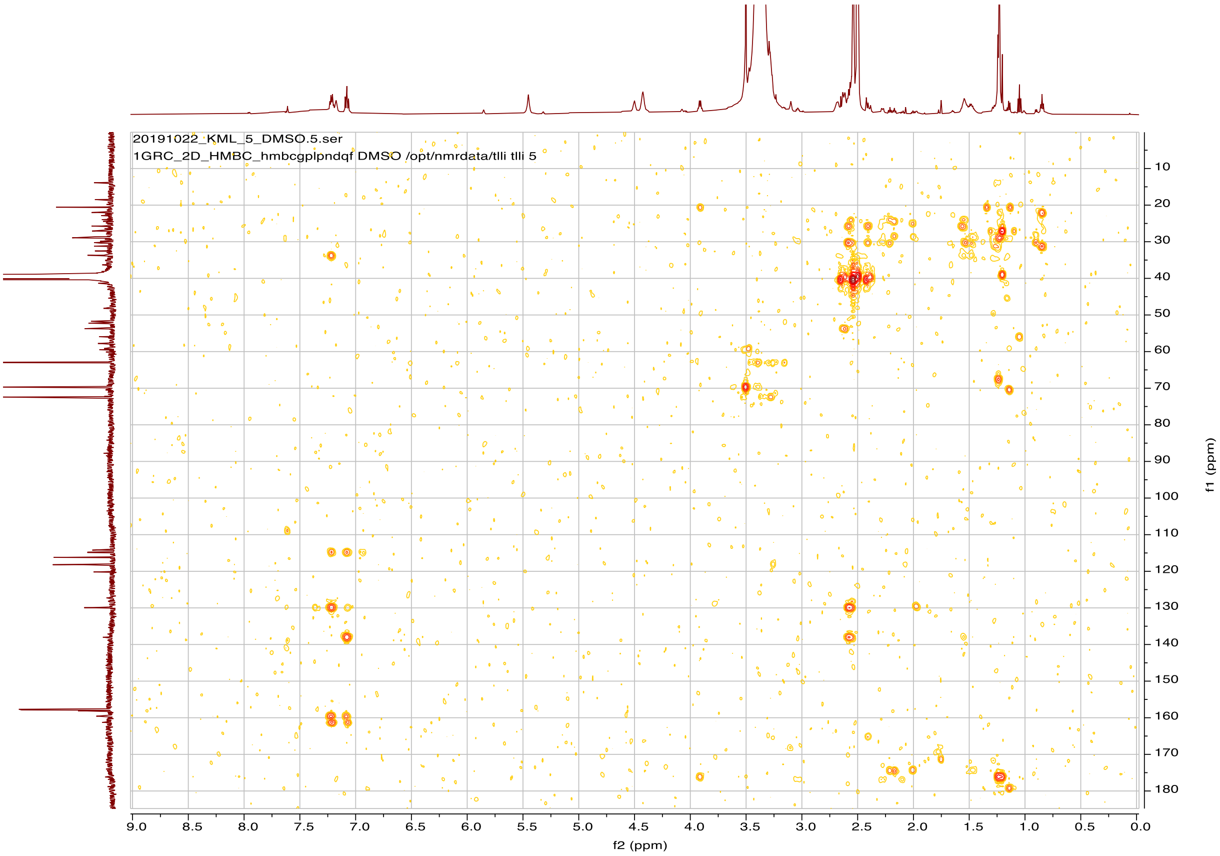

Figure S28: HMBC spectrum of compound **4** in DMSO-d_6

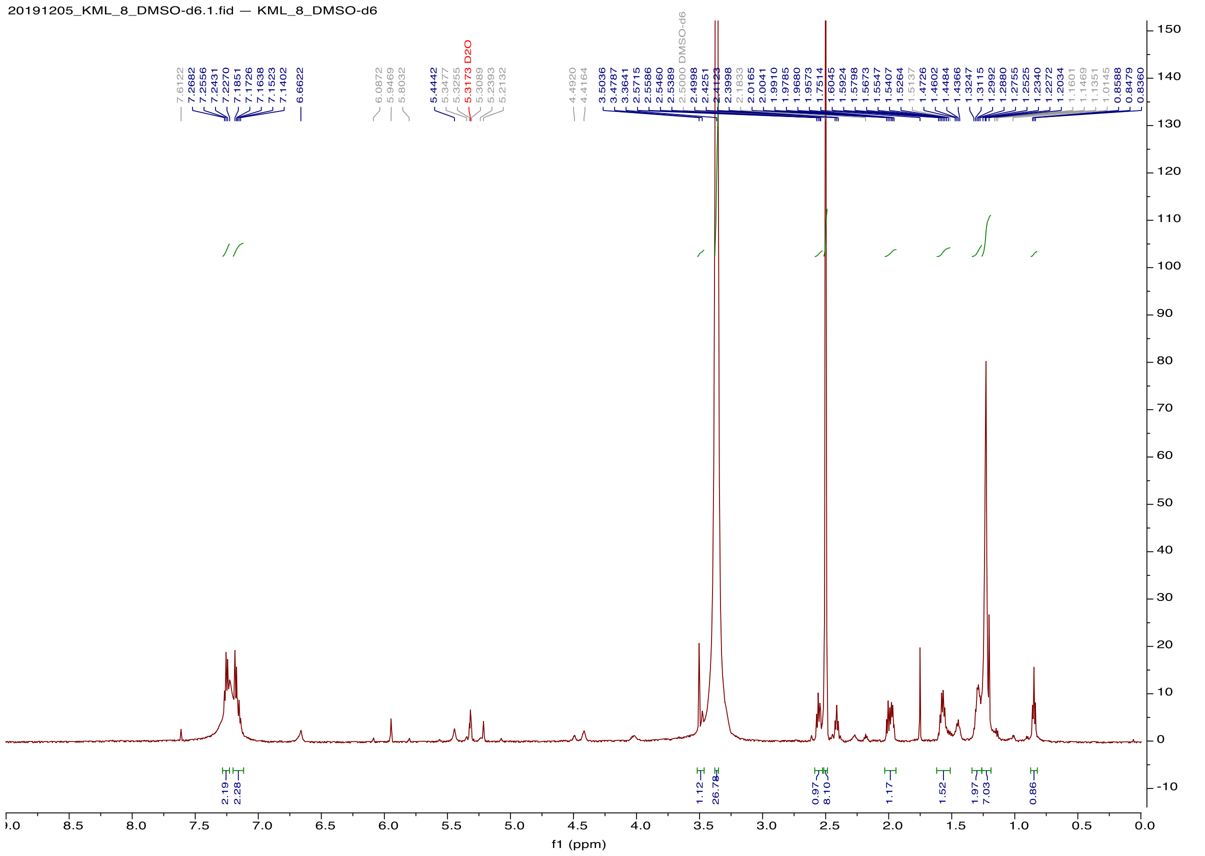

Figure S29: ^{1}H NMR of compound **5** in DMSO-d_6 (600 MHz)

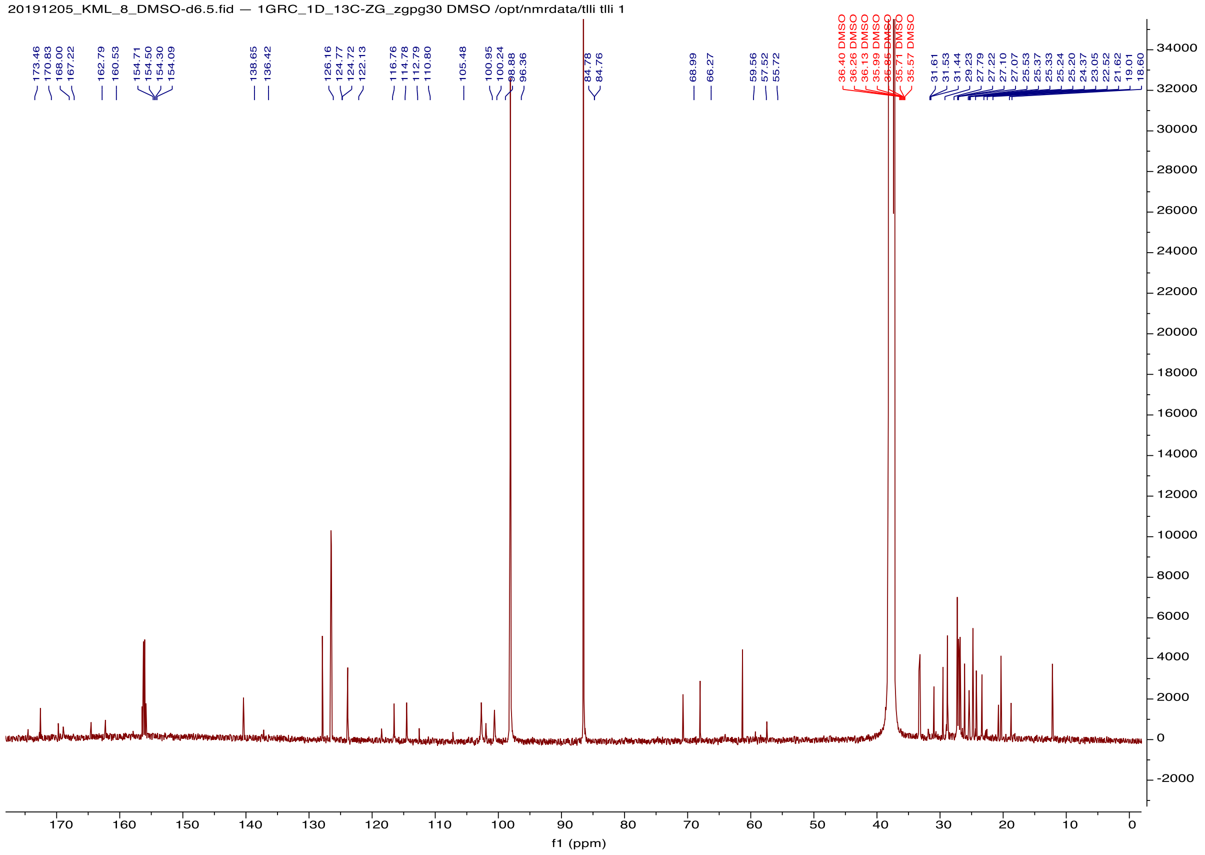

Figure S30: ^{13}C NMR of compound **5** in DMSO-d_6 (150 MHz)

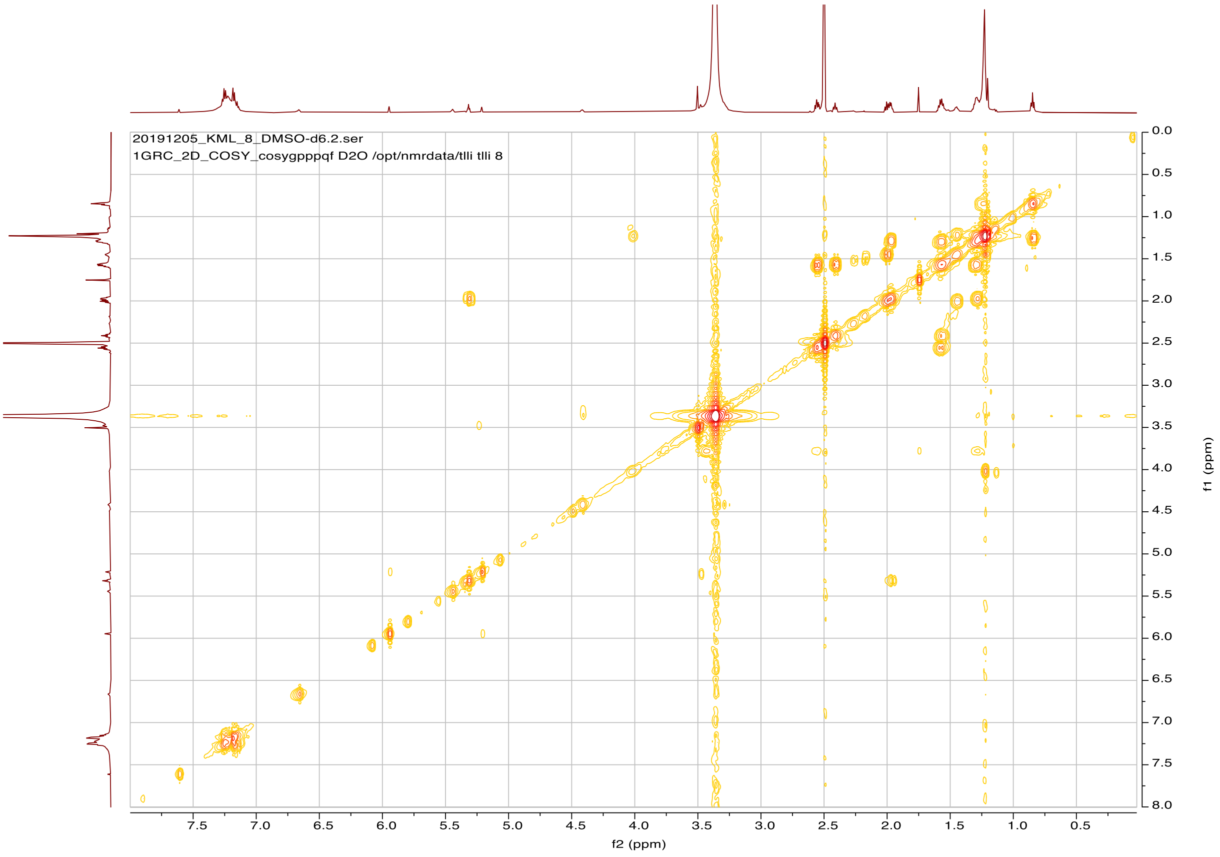

Figure S31: COSY spectrum of compound **5** in DMSO-d_6

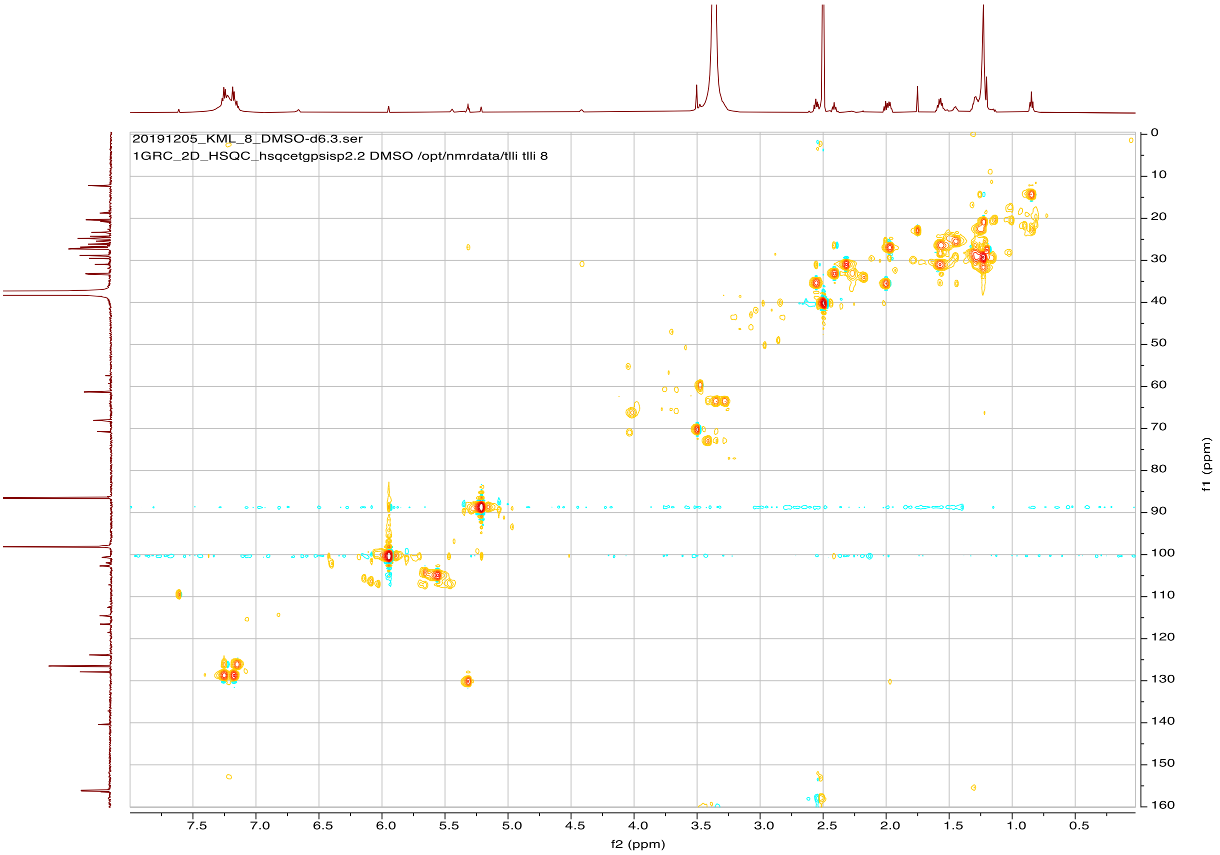

Figure S32: HSQC spectrum of compound **5** in DMSO-d_6

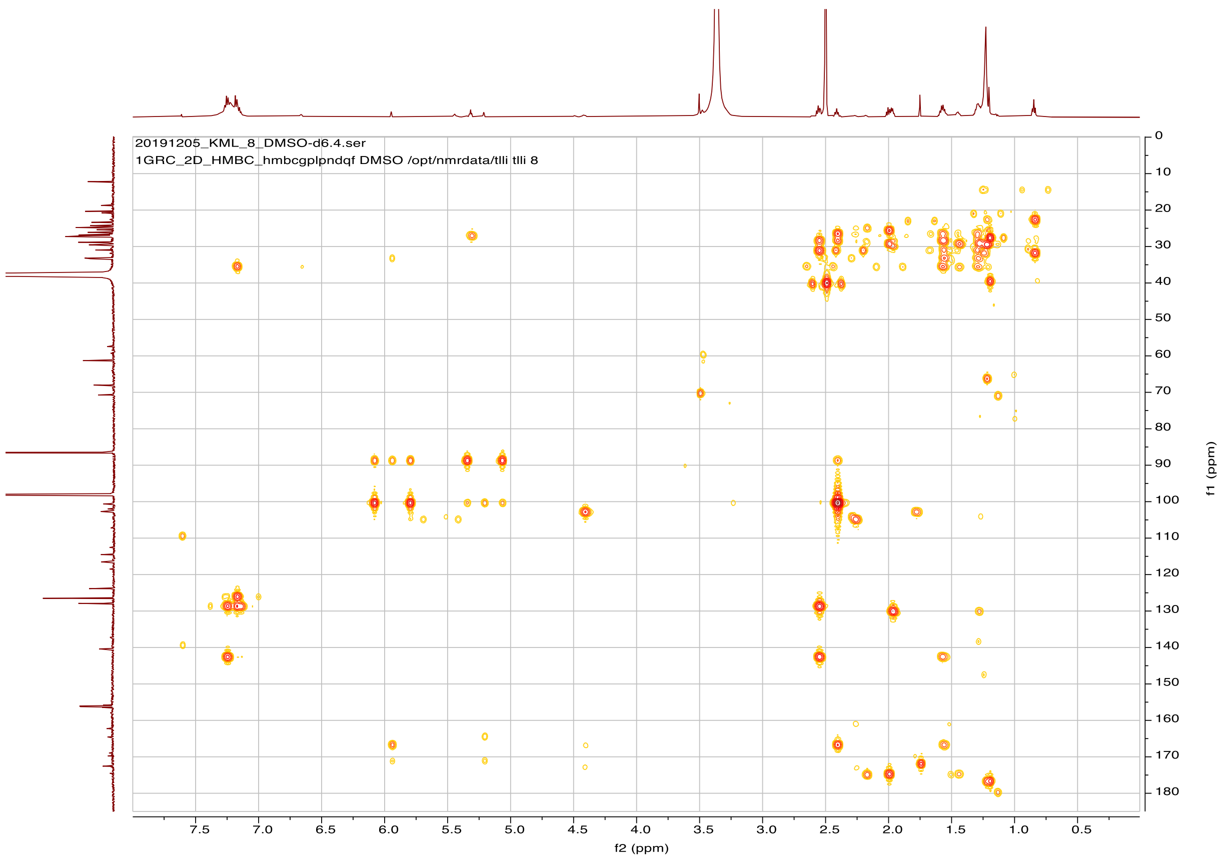

Figure S33: HMBC spectrum of compound **5** in DMSO-d_6

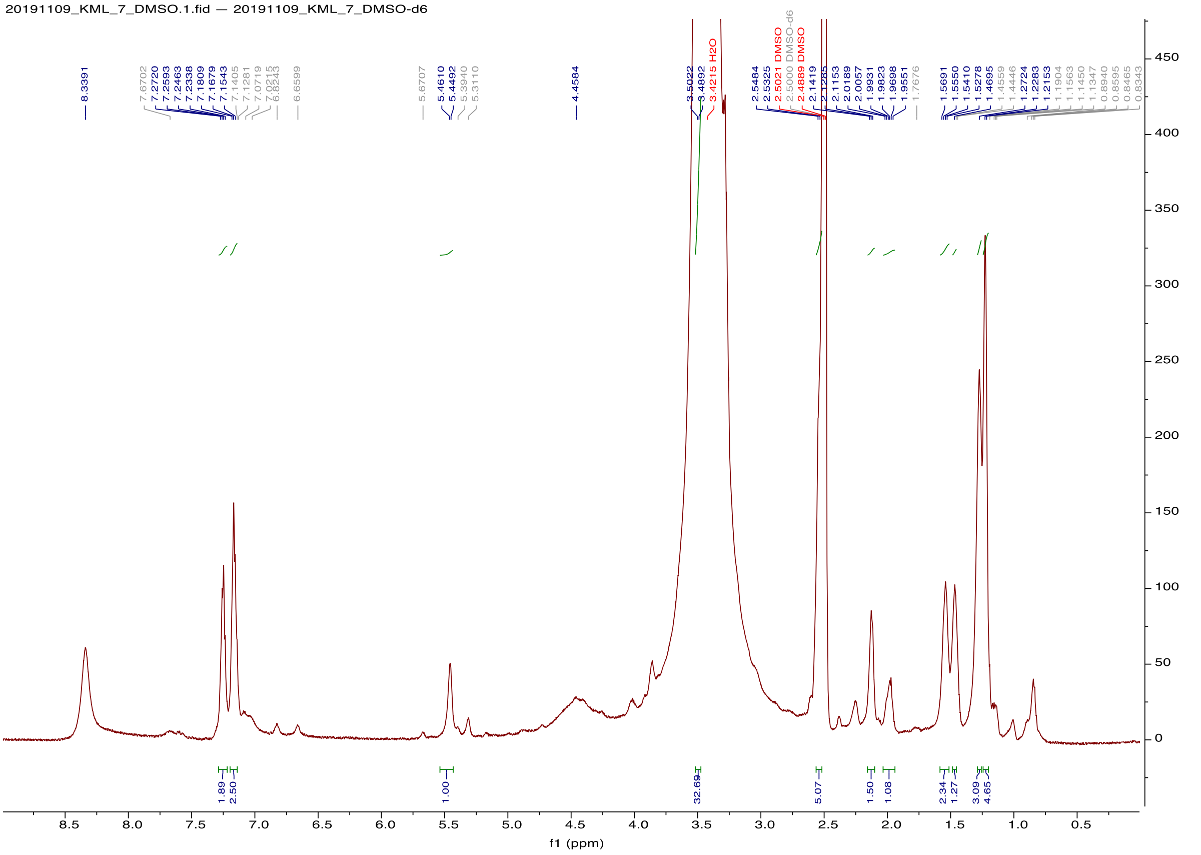

Figure S34: ^{1}H NMR of compound **6** in DMSO-d_6 (600 MHz)

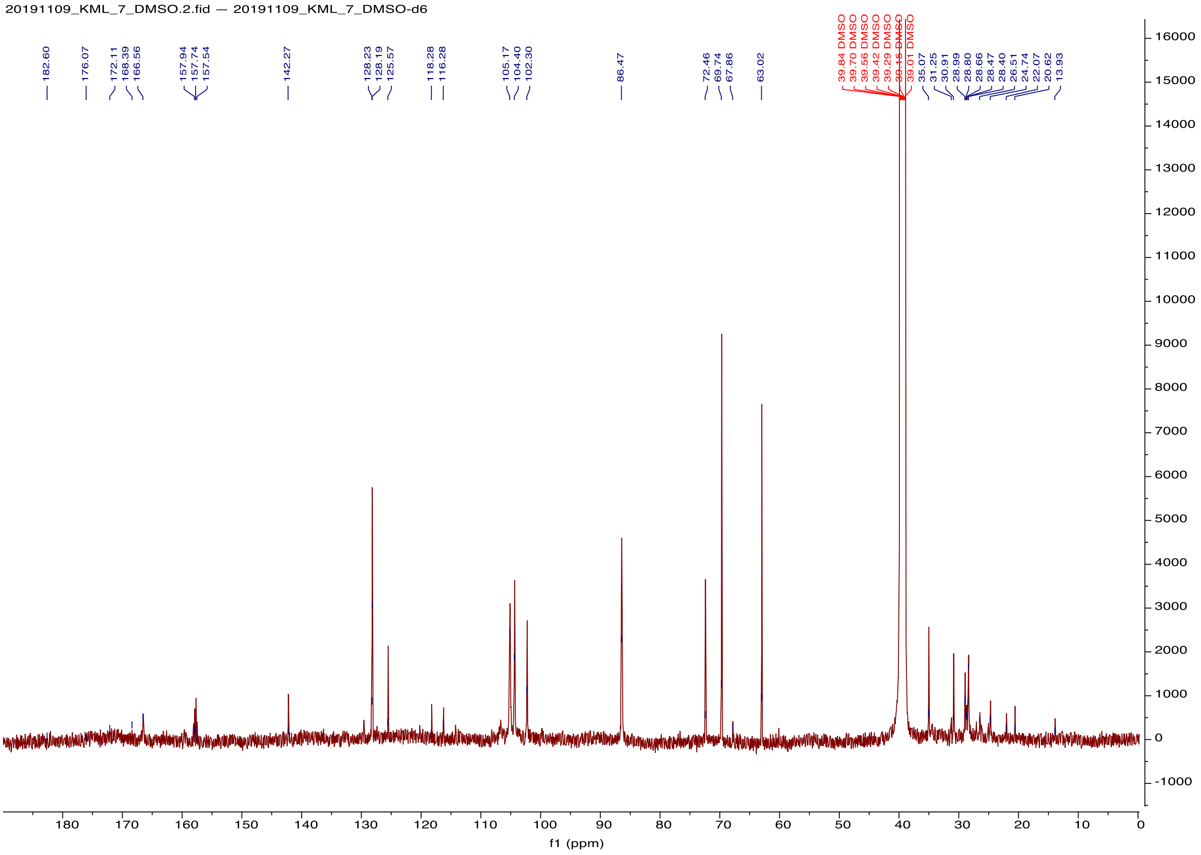

Figure S35: ^{13}C NMR of compound **6** in DMSO-d_6 (150 MHz)

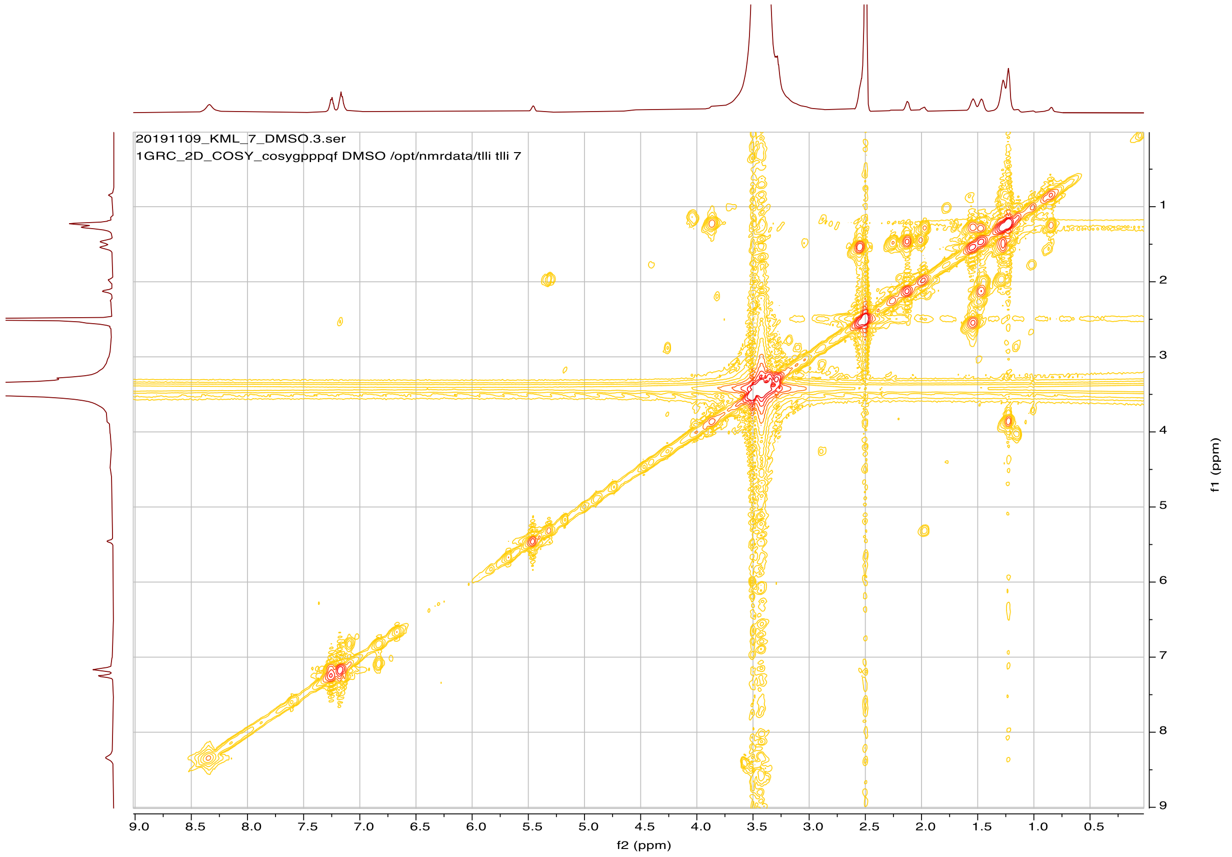

Figure S36: COSY spectrum of compound **6** in DMSO-d_6

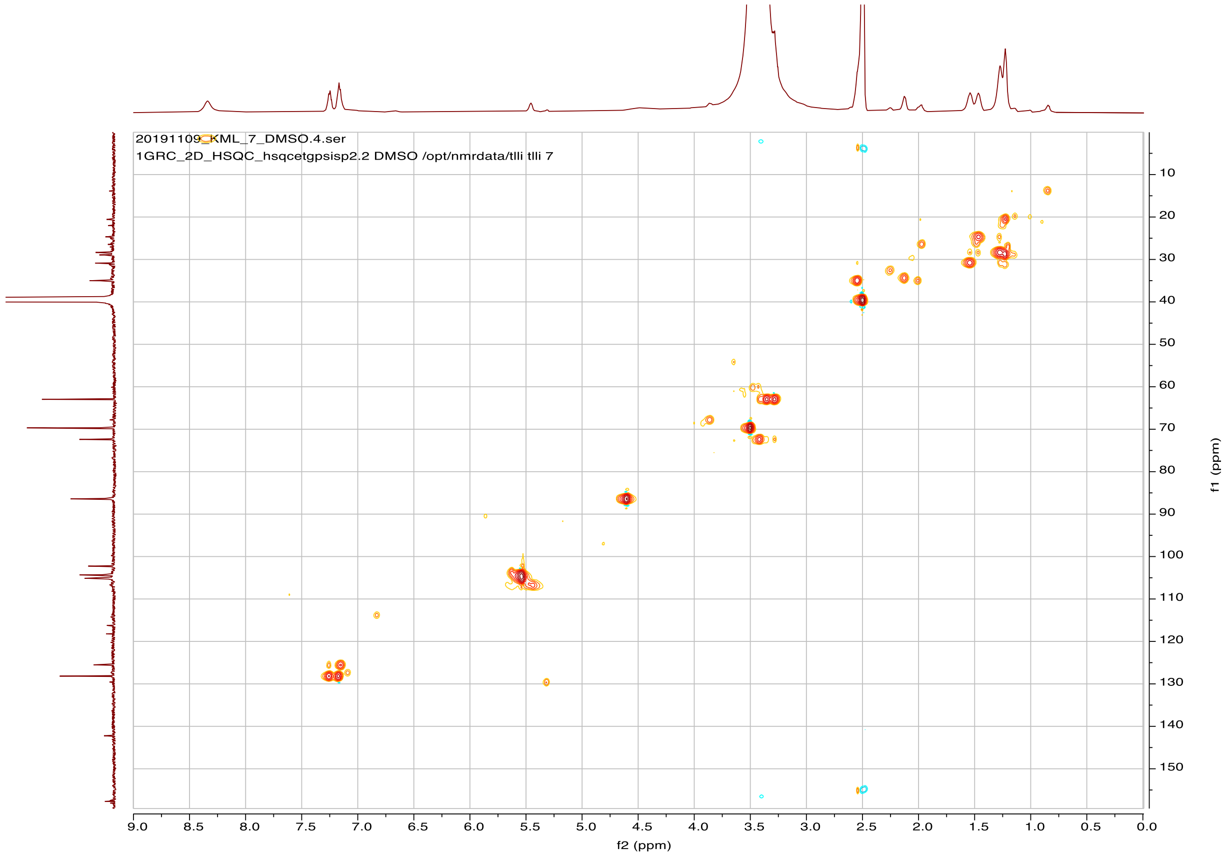

Figure S37: HSQC spectrum of compound **6** in DMSO-d_6

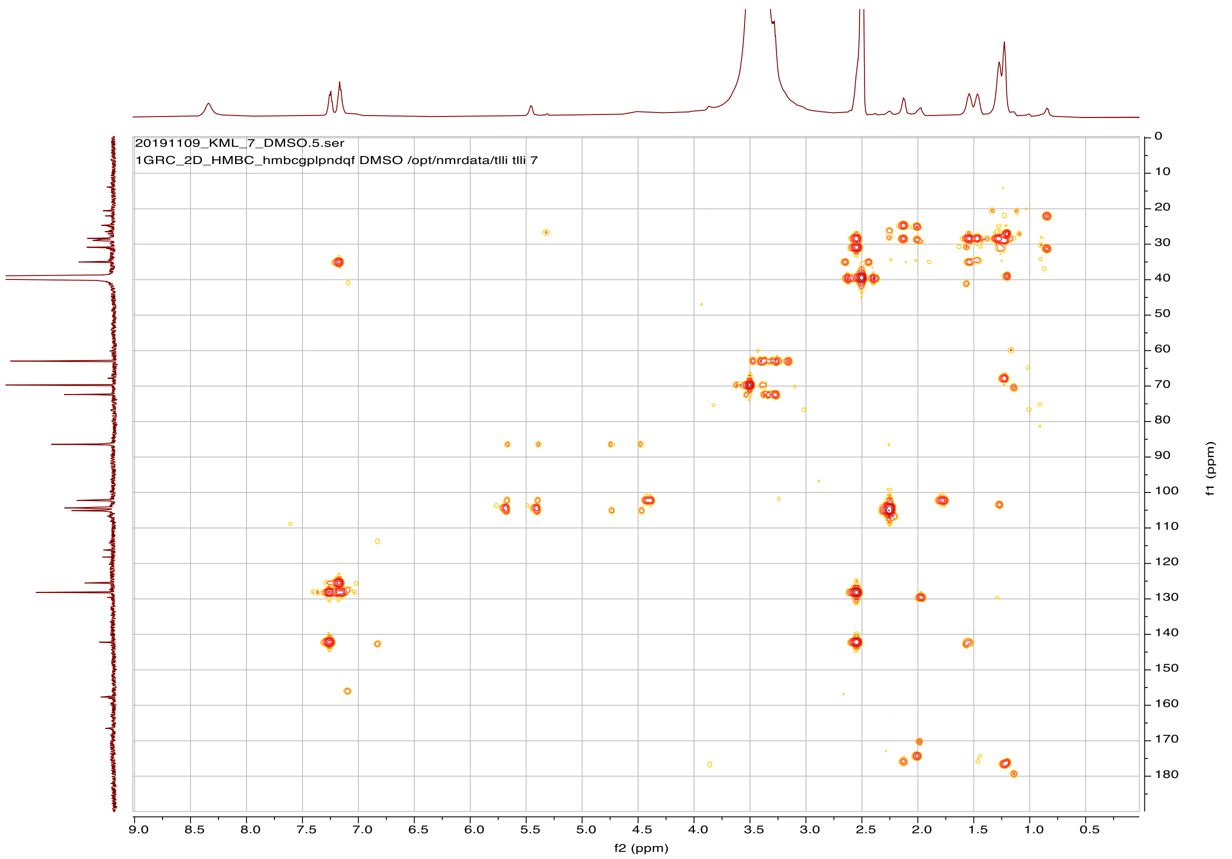

Figure S38: HMBC spectrum of compound **6** in DMSO-d_6

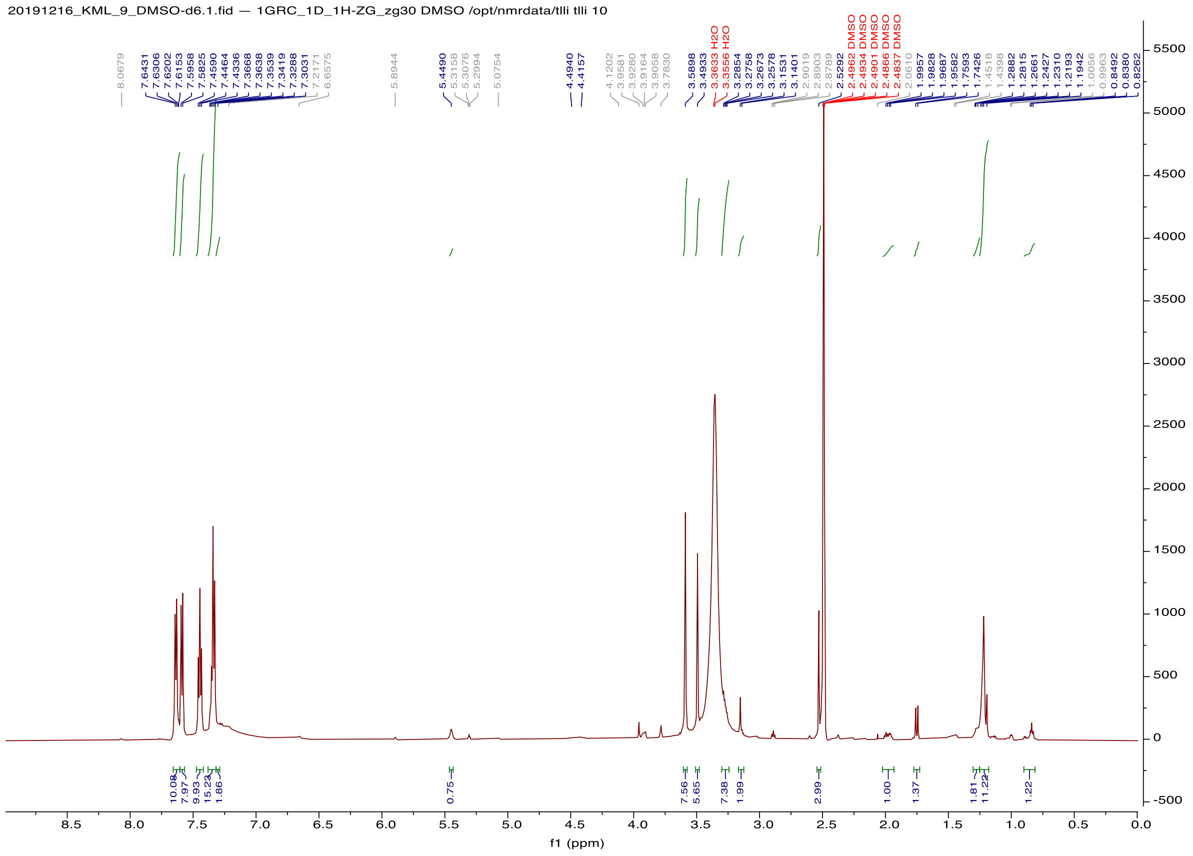

Figure S39: ^{1}H NMR of compound **7** in DMSO-d_6 (600 MHz)

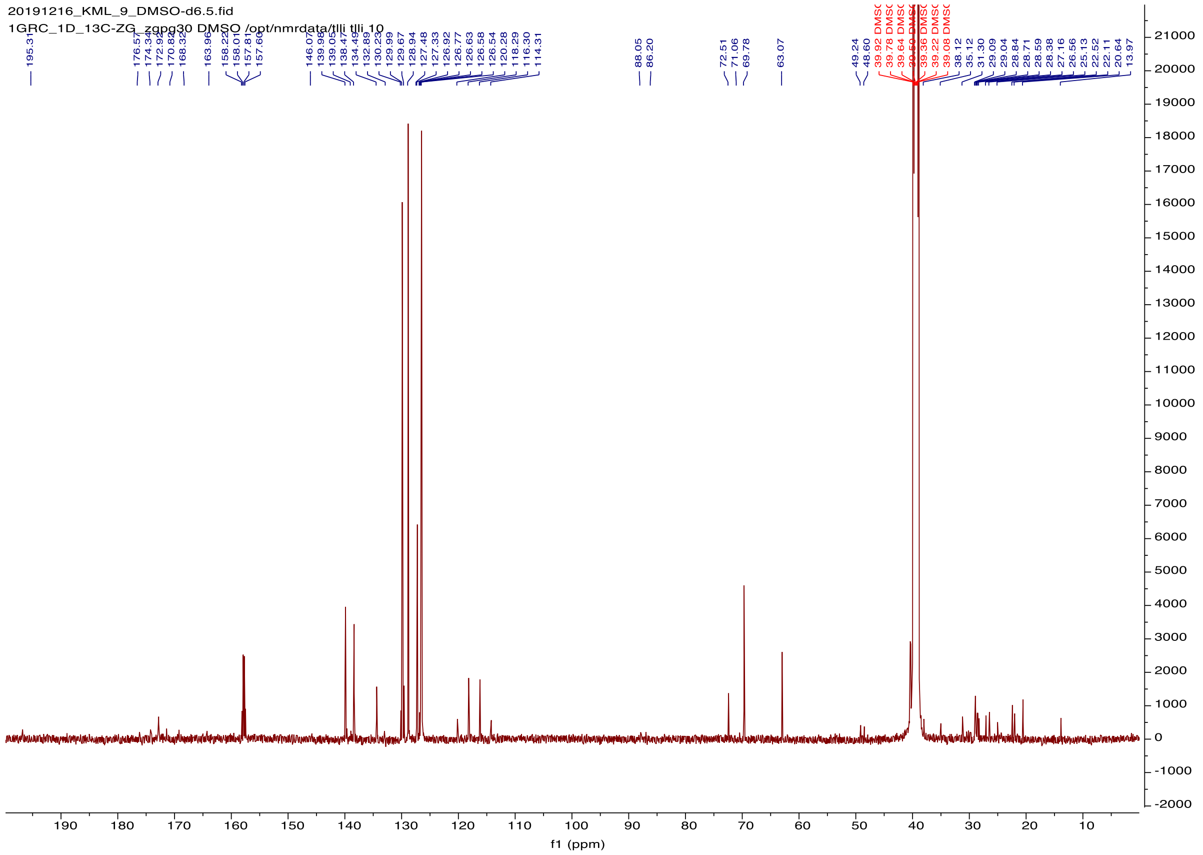

Figure S40: ^{13}C NMR of compound **7** in DMSO-d_6 (150 MHz)

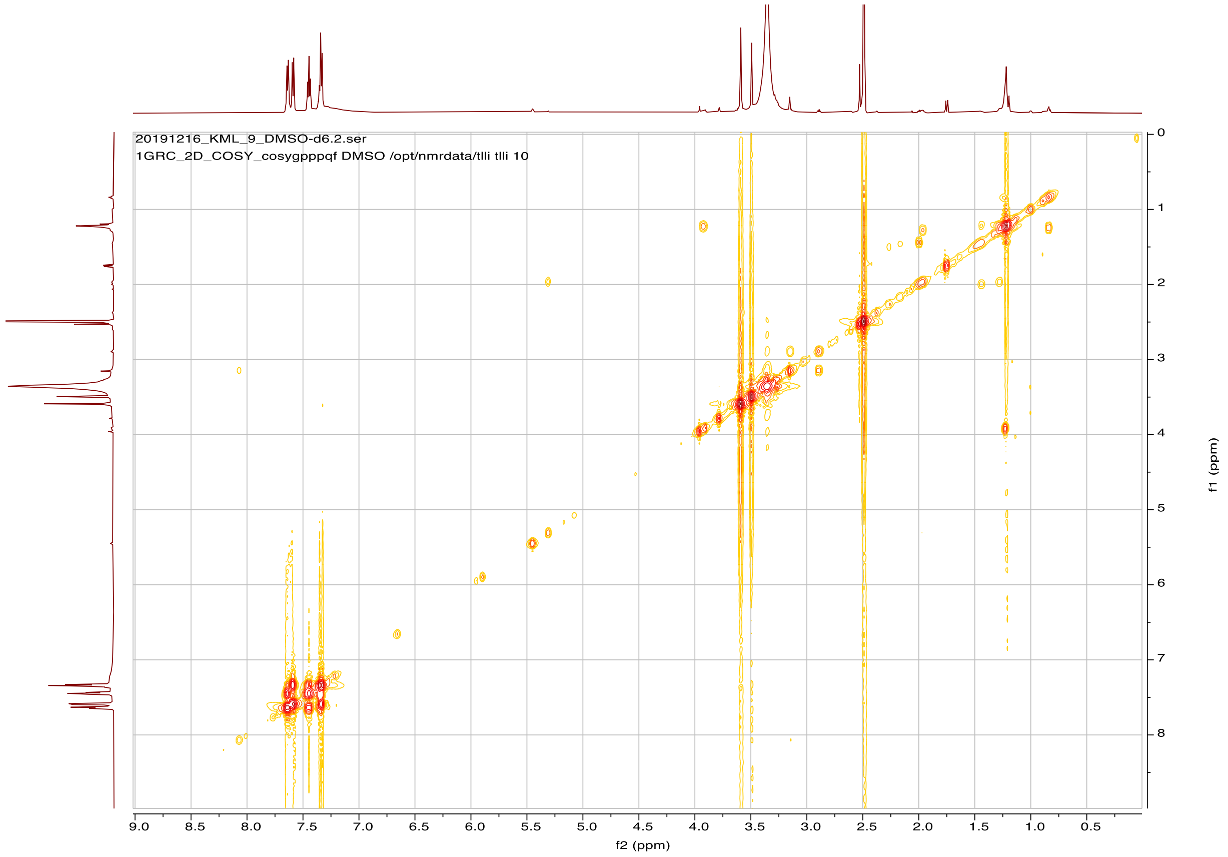

Figure S41: COSY spectrum of compound **7** in DMSO-d_6

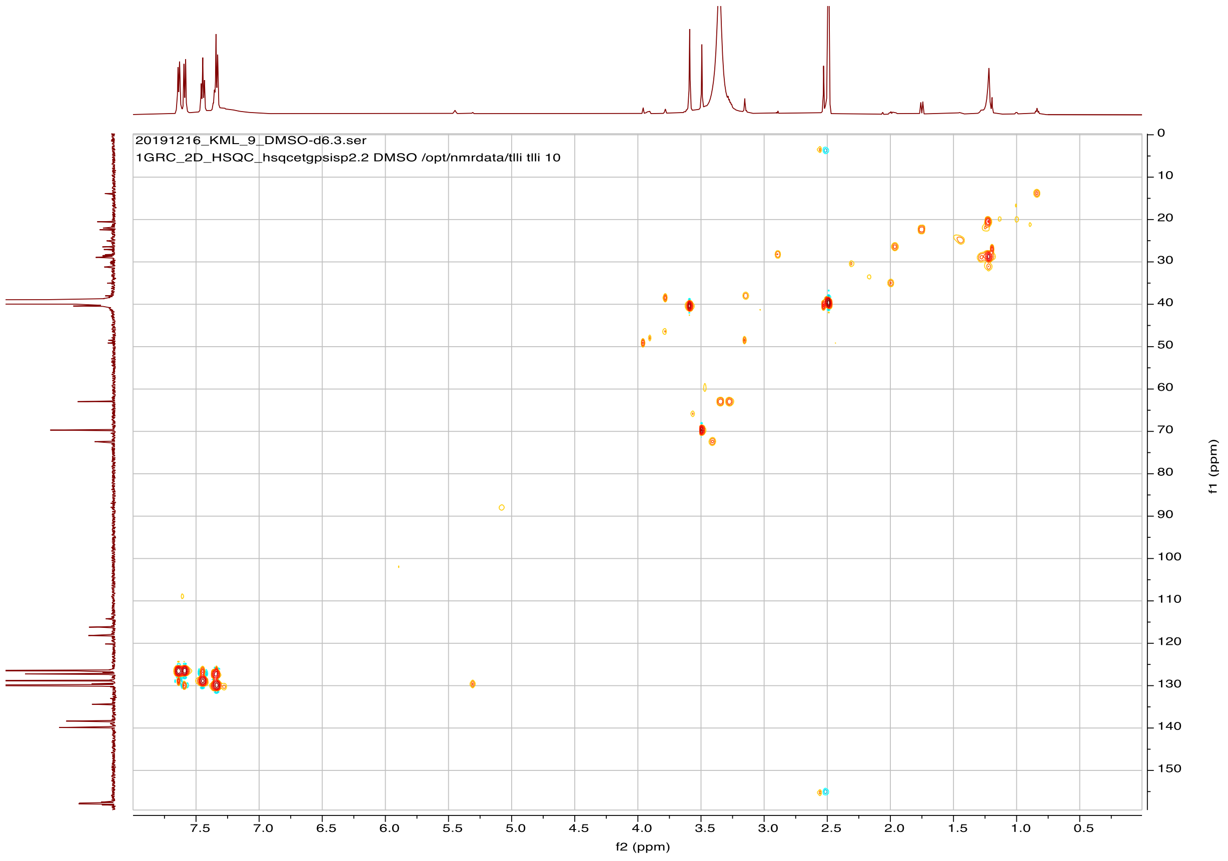

Figure S42: HSQC spectrum of compound **7** in DMSO-d_6

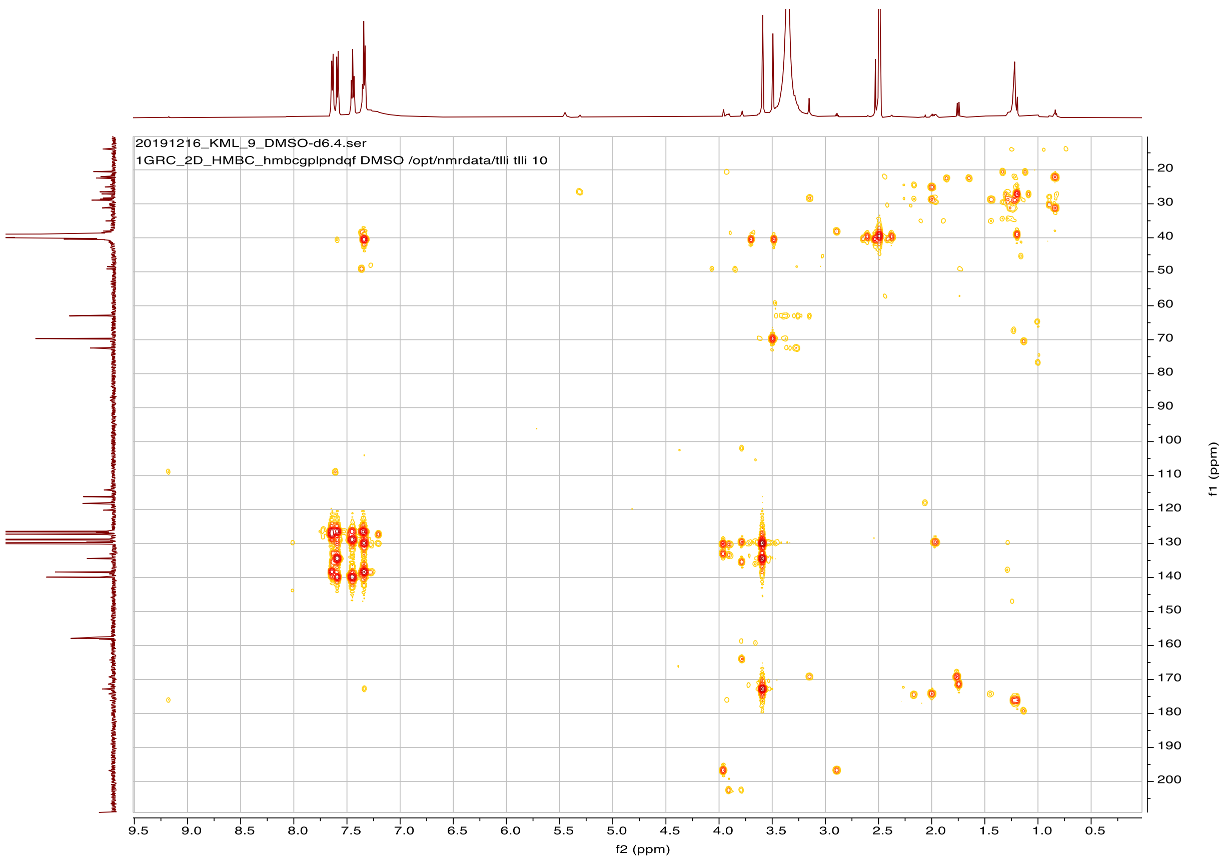

Figure S43: HMBC spectrum of compound **7** in DMSO-d_6
